# Evolution and Expression Analysis of PAO Gene Family in Cotton: Focusing on Fiber Development and Stress Response

**DOI:** 10.3390/plants15101429

**Published:** 2026-05-07

**Authors:** Huixin Gao, Xin Zhou, Fei Wang, Shandang Shi, Manhong Wang, Liping Zhu, Hongbin Li

**Affiliations:** 1 Key Laboratory of Xinjiang Phytomedicine Resource and Utilization of Ministry of Education, Key Laboratory of Oasis Town and Mountain-Basin System Ecology of Bingtuan, College of Life Sciences, Shihezi University, Shihezi 832000, China; hilary_ghx@163.com (H.G.); feiw@shzu.edu.cn (F.W.); shi_shandang@163.com (S.S.); 2 College of Life Sciences, Shaanxi Normal University, Xi’an 710119, China; zhouxin8852@163.com; 3 Department of Poultry Science, Mississippi State University, Mississippi State, MS 39762, USA; mw2911@msstate.edu; 4 National Key Laboratory of Cotton Biological Breeding and Comprehensive Utilization, Henan University, Kaifeng 475004, China

**Keywords:** cotton, *PAO* gene family, fiber development, stress response

## Abstract

Polyamines, a class of low-molecular-weight nitrogen-containing bases with high biological activity, are ubiquitous in organisms and play protective roles in plants under stress. Polyamine oxidase (PAO), a typical flavoprotein characterized as a glycoprotein, is a key enzyme in polyamine catabolism that directly mediates polyamine breakdown and maintains intracellular polyamine homeostasis. However, the specific functions of PAOs in cotton fiber development remain largely unclear. In this study, we identified 23 *GhPAO* genes from the upland cotton (*Gossypium hirsutum* L.) genome via comprehensive bioinformatics approaches. We systematically analyzed their physicochemical properties, phylogenetic relationships, gene structures, chromosomal locations, conserved motifs, *cis*-acting elements, and expression patterns. Quantitative real-time PCR (qPCR) analysis confirmed that *GhPAO10* and *GhPAO21* exhibited the most pronounced transcript accumulation during both fiber development and stress response processes. Further yeast one-hybrid (Y1H) and dual-luciferase reporter assays indicated that the *GhPAO21* promoter was directly regulated by the transcription factor *GhTGA1*. Our findings provide a foundation for elucidating the functional roles of the PAO gene family in upland cotton and underscore potential candidate genes associated with fiber development and stress responses.

## 1. Introduction

Polyamines (PAs), a group of compounds encompassing putrescine (Put), spermidine (Spd), and spermine (Spm), are polycationic alkylamines produced via the decarboxylation of L-ornithine or arginine [1]. These biologically active compounds are crucial for cellular development and have been implicated in responses to pathogenic infections and abiotic stress in plants as well as cancer progression in animals [2]. In plants, PAs are distributed in the cytoplasm and various organelles. Two primary biosynthetic pathways contribute to putrescine production. The first pathway uses ornithine as a precursor, which undergoes a decarboxylation reaction catalyzed by ornithine decarboxylase (ODC) to generate putrescine and carbon dioxide [3]. ODC acts as the rate-limiting enzyme in the broader polyamine biosynthesis pathway. The alternative pathway, mediated by arginine decarboxylase (ADC), constitutes the primary route for putrescine synthesis in plants and certain microorganisms [4]. The subsequent conversion of Put to higher-order PAs, namely Spd and Spm, requires the donation of aminopropyl groups derived from decarboxylated S-adenosylmethionine (dcSAM), a reaction catalyzed by Spd synthase and Spm synthase, respectively [5,6].

Polyamine oxidase (PAO) plays a central role in polyamine catabolism, primarily facilitating the retro-conversion of Spm and Spd to Put [7]. PAO is typically characterized as a homodimeric glycoprotein composed of two identical subunits, each with an apparent molecular weight of approximately 70 kDa. Within the copper-containing amine oxidase (CuAO) subfamily, each subunit contains a single Cu^2+^; consequently, the functional homodimer harbors two copper ions per molecule [8]. Plant PAOs are classified into two distinct subfamilies: copper-containing amine oxidases (*CuAOs*) and flavin adenine dinucleotide (FAD)-dependent PAOs. While *CuAOs* primarily catalyze the oxidation of Put, FAD-dependent PAOs exhibit substrate specificity toward Spd and Spm [9,10]. A conserved lysine residue within the active site has been crucial for the catalytic mechanism of FAD-dependent PAOs [11]. The reaction catalyzed by PAO yields hydrogen peroxide (H_2_O_2_), a key signaling molecule, along with corresponding aldehydes that serve as precursors for further metabolism into compounds such as γ-aminobutyric acid (GABA), thereby modulating plant growth and stress responses [5,12,13]. As a key enzyme directly regulating polyamine catabolism, PAO is essential for maintaining the intracellular polyamine homeostasis. Consequently, through the precise regulation of polyamine homeostasis, PAOs are integral to normal plant development and adaptive responses to environmental stimuli [14,15].

Cotton, a primary source of natural fiber, is a vital global economic crop. Upland cotton (*Gossypium hirsutum* L.), an allotetraploid species, accounts for over 90% of the world’s cotton production [16,17]. Cotton fiber development involves five overlapping stages: initiation, elongation, transition, secondary cell wall thickening, and maturation [18,19]. Understanding the molecular mechanisms that regulate fiber development is critical for improving fiber quality. In recent years, significant progress has been made in characterizing the *GhPAO* gene family in cotton, albeit with a focus on their roles in stress responses. Multiple studies have cloned and functionally characterized individual *GhPAO* genes, confirming their critical contributions to both abiotic and biotic stress adaptation. For example, *GhPAO2* and *GhPAO3* exhibit rapid and distinct transcriptional induction patterns under cold, drought, salinity, and ABA treatments, indicating their involvement in abiotic stress tolerance [20,21,22]. In the context of biotic stress, *GhPAO* members have been shown to actively modulate cotton resistance to *Verticillium wilt* by regulating the dynamics of key defense signals, including H_2_O_2_, salicylic acid, and camalexin [23,24]. Additionally, *PAO* genes are thought to be involved in cotton somatic embryogenesis [25,26], suggesting potential functional roles in plant growth and developmental processes that extend beyond canonical stress response mechanisms.

Collectively, these findings underscore the multifunctional roles of *GhPAO* family members in cotton stress adaptation. Despite these advances, current understanding of the *PAO* family in cotton is constrained by two notable gaps. First, most investigations have focused on individual genes or limited subsets of the family; a comprehensive, updated genome-wide analysis encompassing both cultivated tetraploid cotton and its diploid progenitors have yet to be performed. Second, existing investigations have been largely confined to abiotic and biotic stress responses; notably, few studies, to date, have explored the functional significance of the *PAO* family in cotton fiber development—a trait of paramount agronomic importance. In this study, we conducted a comprehensive genome-wide identification of the *PAO* gene family in *G. hirsutum* and its two diploid progenitors (*G. arboreum* and *G. raimondii*). We systematically characterized their phylogenetic relationships, gene structures, chromosomal distributions, predicted subcellular localizations, and spatiotemporal expression profiles across various tissues and development stages. Compared with previous reports, our work provides a refined and updated identification of the *PAO* family leveraging the latest high-quality cotton genome assemblies. Furthermore, this study represents the first systematic investigation into the potential involvement of *PAO* genes in fiber developmental processes. This study aims to elucidate the potential functional roles of *GhPAO* genes during fiber growth and to identify promising candidate genes for genetic improvement in cotton fiber quality through molecular breeding strategies.

## 2. Results

### 2.1. Identification and Characterization of the GhPAO Family

By performing homology searches using TBtools BLASTp (version 2.390) against the *G. hirsutum* genome database and utilizing *Arabidopsis* PAO protein sequences as queries, we identified 23 putative PAO proteins in upland cotton. The hidden Markov model (HMM) profile for PAO (accession number PF01593) was retrieved from the Pfam database. Subsequently, an HMM-based search of the *G. hirsutum* genome yielded 124 candidate *PAO* genes. The candidate lists generated by two complementary screening approaches—BLASTp and HMM—were subsequently integrated. Ultimately, a final set of 23 non-redundant *GhPAO* genes was identified in the upland cotton genome, and their physicochemical properties were subsequently analyzed (Table 1). The complete nucleotide sequences for the 23 *GhPAO* genes are available as FASTA format in File S1 and detailed in Table S1. In addition, sequence alignment was performed against the latest telomere-to-telomere (T2T) cotton genome assembly, and the corresponding results presented in Table S2. Table 1 summarizes the gene identifier, protein length, molecular weight, isoelectric point (pI), instability index, and grand average of hydrophobicity (GRAVY) for each of the 23 identified GhPAO proteins. Substantial variation was observed in the physicochemical properties of the identified PAO members. Notably, four genes were predicted to encode unusually large proteins (length > 1500 aa, MW: 175–200 kDa), whereas the remaining proteins ranged in length from 488 to 910 aa, corresponding to MWs of 54.02 to 99.02 kDa. The theoretical isoelectric point (pI) varied from 5.31 to 8.90. Of these, five proteins (GhPAO4, GhPAO11, GhPAO16, GhPAO22, and GhPAO23) were predicted to be basic (pI > 7), whereas the remaining 18 were predicted to be acidic (pI < 7). The instability index ranged from 21.16 to 43.73. Ten members (GhPAO1, GhPAO3, GhPAO5, GhPAO10, GhPAO11, GhPAO12, GhPAO13, GhPAO15, GhPAO21, and GhPAO22) were predicted to be unstable, as indicated by an instability index exceeding the threshold of 40. Analysis of the grand average of hydropathicity (GRAVY) values revealed that GhPAO7 and GhPAO18 were hydrophobic in character, while the remaining 21 proteins are predicted to be hydrophilic. Collectively, the observed diversity in key physicochemical parameters suggests potential functional divergence among the PAO family members in upland cotton. Furthermore, subcellular localization prediction revealed diverse targeting patterns among the PAO members. Specifically, two proteins (GhPAO2 and GhPAO6) were predicted to be localized to the chloroplast, three (GhPAO1, GhPAO5, and GhPAO12) to the nuclear, and an additional three (GhPAO10, GhPAO13, and GhPAO21) to the plasma membrane. Four members (GhPAO7, GhPAO18, GhPAO19, and GhPAO20) were predicted to localize to the endoplasmic reticulum, whereas GhPAO8 and GhPAO9 were predicted to be secreted into the extracellular space. The remaining nine proteins were predicated to localize primarily to the cytoplasm. Such compartmentalized distribution strongly suggests that PAO family members may fulfill specialized roles in metabolic regulation within distinct subcellular niches.

### 2.2. Chromosomal Localization and Collinearity

**Figure 1:**
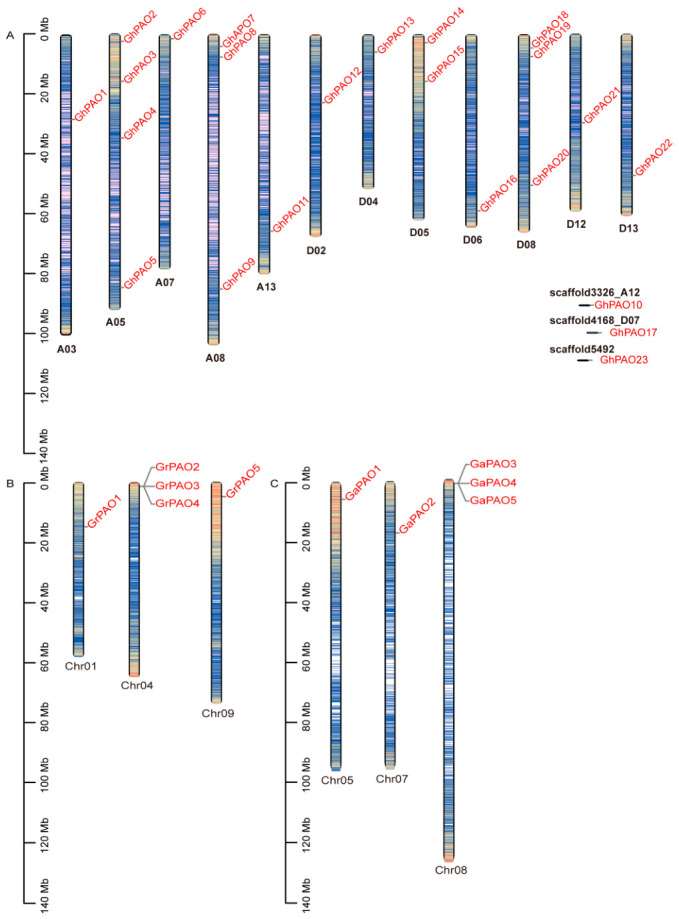
The chromosomal locations of *PAO* in the *G. hirsutum* (**A**), *G. raimondii* (**B**) and *G. arboreum* (**C**) species. The gene ID is displayed on the right, while the gene locus and chromosome length are shown in a vertical layout on the left. Gene density is displayed as a heatmap on the chromosomes, with red and blue representing high and low density regions, respectively. Density was calculated as the number of genes in 1 Mb sliding windows. Mb, megabase.

To elucidate the evolutionary history of *GhPAO* genes across the three cotton species, we analyzed genomic duplication events, with a particular focus on whole-genome duplication (WGD), segmental duplication, and tandem duplication. Gene nomenclature was assigned based on chromosomal localization. The 23 identified *GhPAO* genes were unequally distributed across 12 of the chromosomes in the *G. hirsutum* genome (Figure 1A). Specifically, 11 genes were located on the A sub-genome, distributed across chromosomes A03, A05, A07, A08, A12, and A13. At the chromosome level, the distribution was markedly uneven: chromosomes A05 and A08 carried four and three *PAO* genes, respectively, whereas each of the remaining chromosomes contained only a single copy. The remaining 12 genes were mapped to the D sub-genome on chromosomes D02, D04, D05, D06, D07, D08, D12, and D13, thereby complementing the 11 genes identified on the A sub-genome. Notably, three genes (*GhPAO10*, *GhPAO17*, and *GhPAO23*) were unanchored to unplaced genomic scaffolds rather than to assembled chromosomes. In addition, ten *GhPAO* genes (*GhPAO2*, *GhPAO6*, *GhPAO7*, *GhPAO8*, *GhPAO11*, *GhPAO13*, *GhPAO14*, *GhPAO16*, *GhPAO18*, and *GhPAO19*) exhibited a pronounced clustering near the chromosomal ends (telomeric regions). This telomere-proximal localization suggests a potential role in maintaining chromosomal integrity and modulating telomere-associated cellular processes. Furthermore, to trace the evolutionary trajectory of the PAO families in the two diploid progenitor species, five *PAO* genes were identified in *G. raimondii* (designated *GrPAO1*–*GrPAO5*) and five *PAO* genes in *G. arboreum* (designated *GaPAO1*–*GaPAO5*). Chromosomal localization analysis revealed that *GrPAO* genes are distributed across three chromosomes (Chr01, Chr04, Chr09), with *GrPAO2*, *GrPAO3*, and *GrPAO4* forming a tandem gene cluster on Chr04 (Figure 1B). Similarly, *GaPAO* genes were distributed across three chromosomes (Chr05, Chr07, Chr08), with *GaPAO3*, *GaPAO4*, and *GaPAO5* clustered on Chr08 (Figure 1C). Taken together, this clustered genomic distribution is consistent with segmental duplication events that likely contributed to the expansion of the *PAO* family in diploid cotton progenitors.

**Figure 2:**
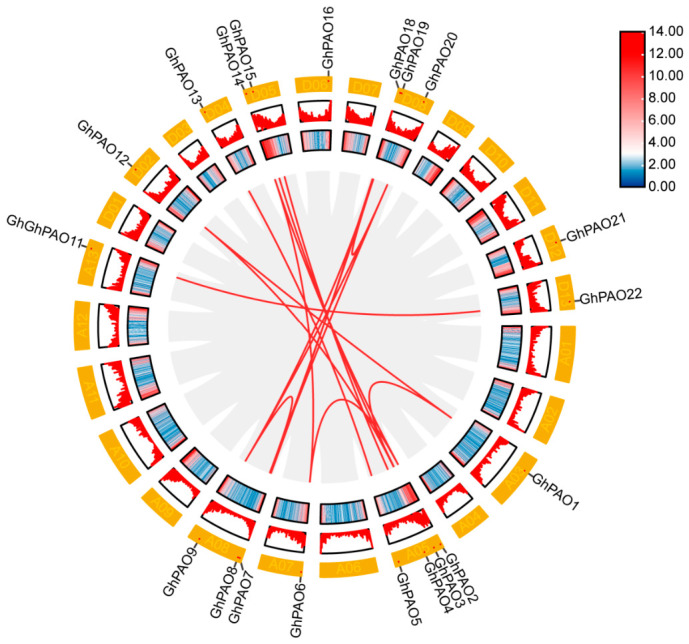
Distribution and duplication of *GhPAO* genes. Note: The outermost ring represents the distribution of *GhPAO* genes on the 12 chromosomes of the *G. hirsutum* genome. Duplicated genes are linked by red lines in the innermost ring.

**Figure 3:**
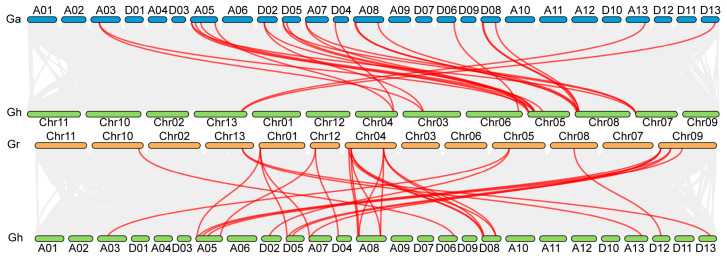
Synteny of *PAO* genes across different cotton species. Chromosomes of *G. arboreum* is blue color on show, and *G. hirsutum* (green), and *G. raimondii* (orange). Red lines represent syntenic relationships among PAO genes.

Collinearity analysis revealed that 20 *GhPAO* genes likely arose through duplication events and were distributed across 12 chromosomes (A03, A05, A07, A08, A13, D02, D04, D05, D06, D08, D12, and D13), whereas *GhPAO10*, *GhPAO17*, and *GhPAO23* were anchored to unplaced genomic scaffolds (Figure 2). Synteny analysis indicated that both tandem and segmental duplication events contributed to the expansion of the *GhPAO* gene family in *G. hirsutum*. We further characterized the *PAO* gene family into three cotton species: *G. hirsutum*, *G. arboreum*, and *G. raimondii*. Comparative genomic analysis between allotetraploid and diploid cotton species suggested that lineage-specific expansion of the *PAO* gene family occurred following polyploidization (Figure 3).

### 2.3. Phylogenetic and Motif Analysis

**Figure 4:**
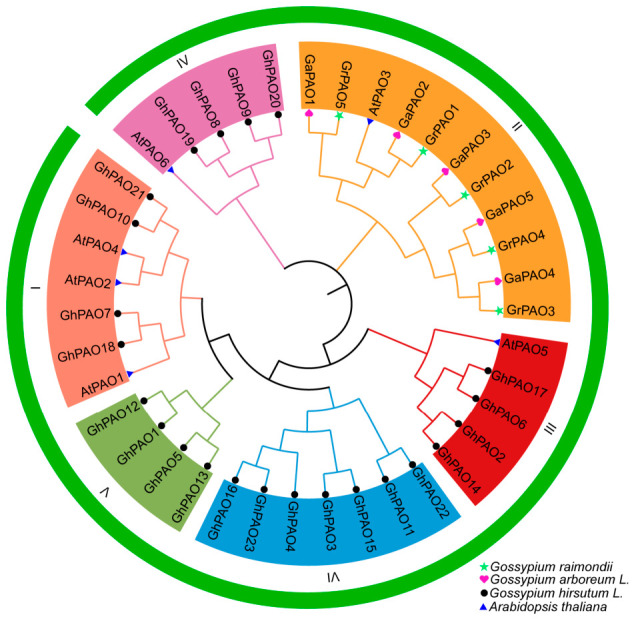
Phylogenetic analysis of PAO proteins. Note: The tree was constructed using protein sequences from *G. raimondii*, *G. arboreum*, *A. thaliana*, and *G. hirsutum*. Construction was performed using the Maximum Likelihood (ML) method. The phylogenetic tree was divided into six subfamilies (I–VI), each marked with a different background color.

In this study, the *GhPAO*, *GaPAO*, and *GrPAO* genes were systematically designated based on their respective chromosomal locations. Phylogenetic analysis of PAO protein sequences from *Arabidopsis thaliana* L. and three cotton species resolved the *GhPAO* family into six distinct clades (Figure 4). Notably, the allotetraploid *G. hirsutum* harbored 23 *PAO* genes, a number approximately four-fold greater than that observed in either of its diploid progenitor species, *G. arboreum* (n = 5) and *G. raimondii* (n = 5).

**Figure 5:**
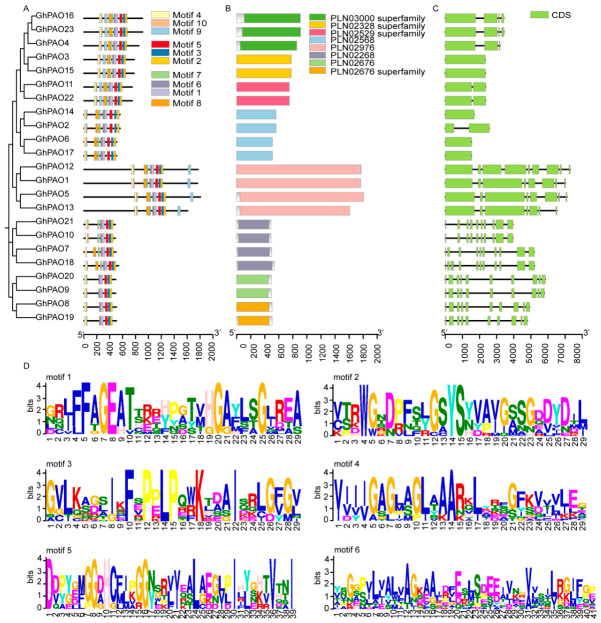
The conserved motif of GhPAO proteins. Distinct motifs are denoted by variant colors to ensure easy recognition. (**A**) A phylogenetic tree constructed based on GhPAO protein sequences. The distribution of conserved motifs identified by MEME is depicted, with each distinct conserved motif marked by a colored box for clear visualization. (**B**) The figure illustrates the spatial distribution of conserved domains, with each distinct color block serving as a visual identifier for the corresponding conserved domain. (**C**) The gene structure of *GhPAO* is delineated, wherein exons are visually represented as green boxes. (**D**) Consensus motif structure of GhPAO. Each amino acid abbreviation is represented by a distinct color.

To investigate the sequence conservation, divergence, and potential roles in abiotic stress response of PAO proteins from *G. hirsutum. G. raimondii* and *G. arboreum*, we subsequently analyzed their conserved motifs using the MEME suite. A total of ten distinct conserved motifs were identified. The structural analysis of PAO encoding genes and conserved domain mapping for *G. raimondii* and *G. arboreum* are presented in Figure S1 and Figure S2, respectively. Although the gene structures were not fully conserved across the *G. hirsutum PAO* gene family, proteins clustering within the same phylogenetic clade tended to share an identical complement of conserved motifs. However, the conserved motif composition exhibited notable variation among different clades. One group contained (GhPAO1, GhPAO5, GhPAO12, and GhPAO13) 10 motifs. A second group (GhPAO2, GhPAO6, GhPAO14, and GhPAO17) shared a set of nine identical motifs. Notably, this group differed from the first solely in the absence of motif 9. A third group (GhPAO3, GhPAO4, GhPAO11, GhPAO15, GhPAO16, GhPAO22, and GhPAO23) possessed a common set of 10 motifs (Figure 5A, Table S4). Membership of the identified proteins in the GhPAO family was further validated by conserved domain analysis. All GhPAO proteins harbored the typical conserved domains of plant polyamine oxidases, particularly the FAD-binding domain (PLN02328/PLN02676 superfamily) and amine oxidase catalytic domain (PLN02529 superfamily), which represent the core functional regions of the PAO family. Taken together, these definitive structural features firmly confirm that all identified GhPAO genes are authentic members of the plant PAO family. Additionally, some GhPAO members harbor plant-specific conserved domains (PLN03000, PLN02568, PLN02976, PLN02268), suggesting potential functional differentiation within the family. For instance, GhPAO21, GhPAO7, GhPAO10, and GhPAO18 shared a common domain of the PLN02268 family. This domain has been implicated in plant immunity and cell wall biosynthesis, thus potentially constituting a primary defense barrier against pathogens. A distinct group comprising GhPAO14, GhPAO2, GhPAO6, and GhPAO17 was found to harbor PLN02568 domain. This domain typifies transporters implicated in mobilization of biosynthetic precursors of the stress-related hormones abscisic acid (ABA) and jasmonic acid (JA), thereby putatively facilitating the transport and spatial distribution of these critical signaling molecules. Thus, these particular genes represent likely candidates as key modulators of plant stress adaptation and developmental processes (Figure 5B). In addition, gene structure and conserved domain analyses of the *PAO* family in *G. raimondii* and *G. arboreum* revealed that all GrPAO and GaPAO proteins possess a core PLN02518 superfamily domain, confirming their assignment as functional members of the *PAO* family (Figures S1B and S2B). The majority of genes exhibited highly conserved motif compositions (motif 1–10) and similar exon–intron architectures, indicating substantial evolutionary conservation within the *PAO* family. Analysis of the *G. hirsutum* genome annotation demonstrated substantial variations in exon numbers among *GhPAO* genes, ranging from a single exon (e.g., *GhPAO3* and *GhPAO5*) to ten exons (e.g., *GhPAO7*, *GhPAO8*, *GhPAO9*, *GhPAO10*, *GhPAO18*, *GhPAO19*, *GhPAO20*, and *GhPAO21*) (Figure 5C). This observation highlights the distinct exon–intron structural patterns among *GhPAO* family members, which have likely contributed to functional diversification (Figure 5C). Although subtle differences in gene structure were detected between *GrPAOs* and *GaPAOs* with respect to intron number, domain length, and untranslated region (UTRs) (Figures S1C and S2C), such variations likely reflect species-specific evolutionary divergence and functional adaptation during cotton polyploidization. Furthermore, we observed that all GhPAOs proteins shared a core set of six conserved motifs (motifs 1–6). Notably, these motifs exhibited an invariant 5′ to 3′ sequential arrangement: motif 4→6→1→5→3→2 (Figure 5D).

Collectively, although the *PAO* gene families across the three cotton species exhibit clade-specific and species-specific structural variations, their core structural features are highly conserved. The presence of both conserved core motifs and canonical PAO-related domains ensure the fundamental catalytic functions of these proteins. Conversely, structural differences in gene architecture, motif organization, and the presence of unique auxiliary domains likely reflect the functional diversification that occurred in cotton during evolutionary processes and polyploidization. Taken together, these structural characteristics provide a robust foundation for future efforts to elucidate the mechanisms underlying functional specialization of *GhPAO* genes in plant development and stress responses.

### 2.4. Cis-Regulatory Element Analysis

**Figure 6:**
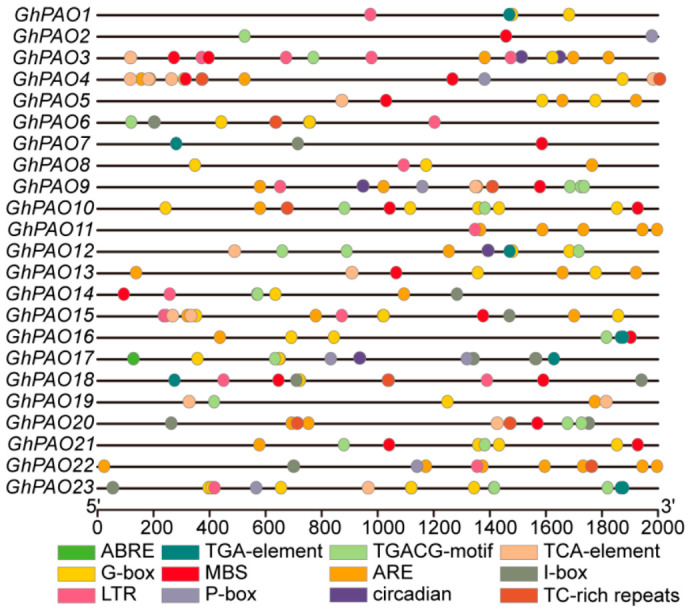
Perform a comprehensive examination of the *cis*-acting elements within the upstream region of the *GhPAO* genes promoter. Distinctively colored boxes serve as visual indicators for the *cis*-acting elements that are uniquely identified.

To investigate the potential involvement of *GhPAO* genes in abiotic stress responses, we conducted a comprehensive analysis of *cis*-acting regulatory elements. Specifically, we examined the 2000 bp promoter regions upstream of the translation start site for GhPAO gene. Based on functional annotation, we identified and curated 12 *cis*-acting regulatory elements (Figure 6) with known roles in plant developmental processes, hormone responsiveness, and responses to abiotic and biotic stresses. These identified *cis*-acting regulatory elements were broadly categorized into two major functional groups. The first group, stress-responsive elements, encompassed those associated with low-temperature responsiveness (LTR), drought inducibility (MBS), light responsiveness (G-box, I-box), anaerobic induction (ARE), and defense signaling (TC-rich repeats). The second group, developmental regulation, comprised a single circadian control element. Promoter analysis of the *GhPAO* genes revealed the presence of multiple regulatory elements associated with hormone signaling pathways. These included the methyl jasmonate-responsive (MeJA) TGACG-motif, the gibberellin-responsive P-box, and the salicylic acid-responsive TCA-element. *GhPAO3*, *GhPAO15*, and *GhPAO23* were predicted to harbor the highest number of *cis*-acting regulatory elements, whereas *GhPAO2* and *GhPAO7* contained the fewest, with only three each. Furthermore, promoter analysis revealed that the G-box motif was the most prevalent *cis*-element among the *GhPAO* genes, representing 58 total occurrences. Collectively, light-responsive elements (G-box and I-box) accounted for 72 occurrences, whereas hormone-responsive elements (TGACG-motif, P-box, TCA-element) totaled 49. These findings suggest a potential role for *GhPAO* genes in both light perception and hormone signaling pathways. Moreover, the abundance and diversity of hormone-responsive elements identified herein strongly suggest that *GhPAO* expression is subject to intricate multifactorial hormonal regulation, potentially implicating these genes in hormone-mediated fiber development.

### 2.5. Expression Patterns of GhPAO Genes

**Figure 7:**
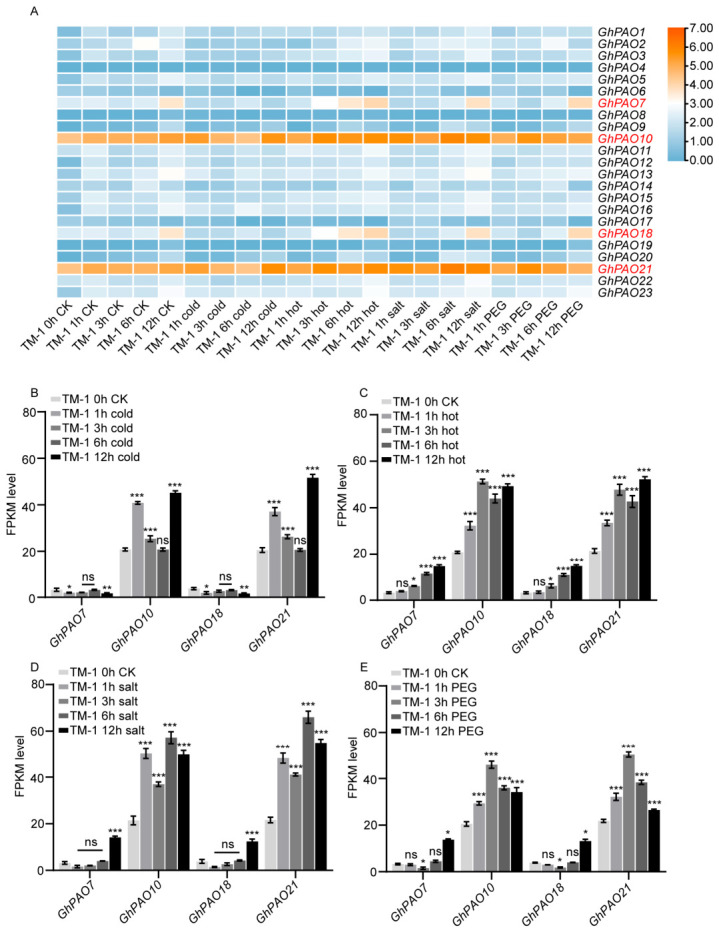
Expression analysis of *GhPAO*s under different treatment conditions. (**A**) Expression of *GhPAO*s under cold, heat, PEG, and salt treatments. Genes highlighted in red were selected for further analysis. The heatmap shows Log_2_(FPKM + 1) values of *GhPAO*s, with blue indicating low expression and orange means high. (**B**) Expression of *GhPAO7*, *GhPAO10*, *GhPAO18* and *GhPAO21* under cold treatment in different time points. (**C**) Expression of *GhPAO7*, *GhPAO10*, *GhPAO18* and *GhPAO21* under hot treatment at different time points. (**D**) Expression of *GhPAO7*, *GhPAO10*, *GhPAO18* and *GhPAO21* under salt stress treatment at different time points. (**E**) Expression of *GhPAO7*, *GhPAO10*, *GhPAO18* and *GhPAO21* under PEG drought treatment at different time points. FPKM (Fragments Per Kilobase of exon model per Million mapped fragments) is a relative quantification metric in transcriptome sequencing used to normalize for gene length and sequencing depth. Error bars are ± SD (n = 3), and asterisks indicate significant differences by *t*-test: * *p* ≤ 0.05, ** *p* ≤ 0.01, *** *p* ≤ 0.001, and ns, not significant.

To investigate the functional roles of the *GhPAO* gene, we analyzed publicly available RNA sequencing data to characterize the expression profiles across different tissues of upland cotton, including roots, stems, leaves, flowers, ovules at 0 days post-anthesis (DPA), and fibers at various developmental stages, as well as transcriptomic responses under cold, heat, drought, and salt stress treatments. Overall, the 23 *GhPAO* family members showed divergent expressions under cold, heat, salt and PEG-simulated drought stress. Among them, *GhPAO7*, *GhPAO10*, *GhPAO18* and *GhPAO21* displayed obvious stress-responsive changes (Figure 7A). We further analyzed their dynamic FPKM levels across sequential time points under each treatment. Under cold stress (Figure 7B), *GhPAO21* was rapidly induced at 1 h, temporarily decreased afterward, and gradually rose to its peak at 12 h with the highest expression level. *GhPAO10* presented a similar trend and also peaked at 12 h, with consistently lower abundance and milder expression fluctuations than *GhPAO21*. In contrast, *GhPAO7* and *GhPAO18* maintained low basal expression and showed no significant alterations relative to the control, with negligible response to cold stress. Under heat stress (Figure 7C), expression patterns were largely consistent with cold stress. *GhPAO21* increased at 3 h, slightly declined, and peaked again at 12 h. *GhPAO10* followed an analogous trend, remaining less expressed than *GhPAO21*. *GhPAO7* and *GhPAO18* still showed constitutively low expression without notable stress induction. Under salt stress (Figure 7D), *GhPAO10* and *GhPAO21* exhibited highly synchronous dynamics. Both were rapidly upregulated at 1 h, slightly reduced at 3 h, peaked at 6 h, and moderately declined at 12 h. *GhPAO21* remained marginally higher than *GhPAO10* from 3 to 12 h. *GhPAO7* stayed stably low-expressing, while *GhPAO18* only showed weak, non-significant late induction. Under PEG drought stress (Figure 7E), *GhPAO10* and *GhPAO21* presented divergent inductive patterns. *GhPAO21* peaked at 3 h followed by pronounced decline, whereas *GhPAO10* reached its peak at 6 h and only underwent mild reduction afterward, eventually surpassing *GhPAO21* at 12 h. As with the other three stresses, *GhPAO7* and *GhPAO18* remained constitutively low-expressing with almost no obvious stress response under drought treatment. Collectively, *GhPAO7* and *GhPAO18* maintained persistently low basal expression with negligible stress responses across all treatments. By contrast, *GhPAO10* and *GhPAO21* possessed markedly high transcript abundance and robust stress-inducible profiles. Therefore, these two genes were selected for subsequent qPCR validation and functional investigation. These results reveal that the A/D sub-genome homeologs *GhPAO10* and *GhPAO21* exhibit slightly different expression patterns under abiotic stresses, which support the adaptive response of cotton to adverse environments.

**Figure 8:**
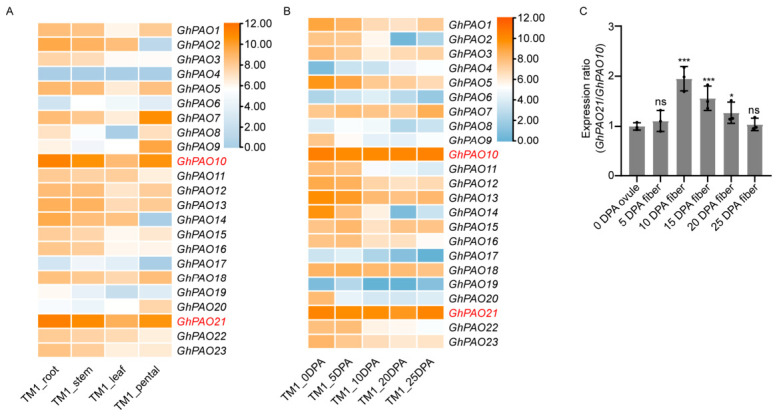
Expression profiling of *GhPAO* genes in various tissue sites of cotton. (**A**) Expression of *GhPAO*s in root, stem, petal, and leaf. (**B**) *GhPAO*s expression in cotton at different developmental stages. The heatmap shows Log_2_(FPKM + 1) values of *GhPAO*s, with blue indicating low expression and orange means high. *GhPAO10* and *GhPAO21* (marked in red) were selected for subsequent functional validation (**C**) Expression ratio of *GhPAO21* relative and *GhPAO10* genes at different stages of cotton development. The expression of *GhPAO*s was normalized to the expression of *GhUBQ7*. Error bars are ± SD (n = 3), and asterisks indicate significant differences by *t*-test: * *p* ≤ 0.05, *** *p* ≤ 0.001, and ns, not significant.

Tissue-specific expression analysis (Figure 8A) revealed that most *GhPAO* genes exhibited distinct expression patterns across roots, stems, leaves, and petals of upland cotton (cv. TM-1). Among them, *GhPAO10* and *GhPAO21* exhibited constitutively high expression levels across all tested tissues (the color scale ranging from blue to orange denotes expression levels from low to high), suggesting their fundamental role in the regulation of cotton growth and development. Conversely, other genes, including *GhPAO4* and *GhPAO17* exhibited lower expression levels in most tissues, underscoring the functional diversification within this gene family.

Analysis of the fiber development stages (Figure 8B) revealed that *GhPAO10* and *GhPAO21* maintained consistently high expression levels across all developmental stages examined, implicating these genes in fiber elongation and secondary cell wall biosynthesis. In contrast, several *GhPAO* genes (e.g., *GhPAO2*, *GhPAO14*) exhibited stage-specific expression patterns characterized by peak expression at discrete fiber development time points, indicating their stage-specific regulatory processes during fiber development.

To further delineate the functional contributions of *GhPAO10* and *GhPAO21*, we quantified their respective expression levels across different fiber developmental stages by qPCR (Figure S3). Furthermore, to more clearly discern the relative expression differences between *GhPAO10* and *GhPAO21*, their expression ratios were calculated for ovules and fibers (Figure 8C). The *GhPAO21* and *GhPAO10* expression ratio was relatively low at 0 DPA and 5 DPA but exhibited a marked increase at 10 DPA and 20 DPA (*p* < 0.001), indicating that *GhPAO21* may play a predominant role relative to *GhPAO10* during the rapid fiber elongation and secondary wall thickening stages. In contrast, no significant difference in the expression ratio was observed at 25 DPA, suggesting comparable functional contributions of the two genes during late fiber maturation. Taken together, compared to its homolog *GhPAO10*, *GhPAO21* exhibited significantly higher expression levels during critical developmental stages, including rapid fiber elongation (10 DPA) and secondary wall thickening (20 DPA), underscoring its potentially more central role in cotton fiber development. Furthermore, the homologous genes in the A and D sub-genomes exhibited coordinated expression patterns, suggesting that these genes likely play critical and evolutionarily conserved roles in fiber development. Based on these comprehensive expression pattern analyses, *GhPAO21* was selected as the primary candidate for subsequent functional characterization and regulatory studies.

### 2.6. TGACG-Motif Analysis in GhPAO Promoters

Previous transcriptomic analysis revealed that the MeJA signaling pathway is associated with fiber initiation and may also contribute to the regulation of fiber elongation [27]. Furthermore, exogenous application of suitable MeJA concentrations promotes cotton fiber initiation and elongation [28,29]. To identify TFs capable of binding to the MeJA-responsive *cis*-element (TGACG-motif) in the promoter regions of *PAO* genes and regulating their expression. Based on our comprehensive expression analysis, *GhPAO21* exhibited higher overall transcript abundance and a more distinct expression advantage during key fiber developmental stages. As shown in Figure 8C, *GhPAO21* expressed significantly higher expression levels than *GhPAO10* across all fiber developmental stages examined, and its expression profile closely coincides with the rapid fiber elongation phase. Notably the promoter of *GhPAO21* harbors MeJA-responsive *cis*-regulatory elements, which have been closely associated with the regulation of fiber development. Therefore, we selected *GhPAO21* as the primary candidate, for further investigation, aiming to identify TFs potentially regulated its expression through the MeJA-responsive *cis*-acting element (TGACG-motif) located within its promoter region. This analysis identified eight candidate TFs.

**Figure 9:**
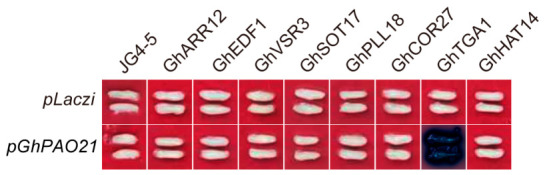
Yeast one-hybrid (Y1H) assay assessing the interaction between the *GhPAO21* promoter and eight candidate TFs. Note: pLacZi-*GhPAO21* denotes the bait vector containing the TGACG-motif; pJG4-5 presents the empty prey vector into which all candidate TFs coding sequences were individually cloned. Blue yeast colonies indicate a positive interaction between the cognate TF and the *GhPAO21* promoter, whereas the white colonies reflect the absence of detectable binding activity. Co-transformation with the empty pJG4-5 prey vector and the pLacZi-*GhPAO21* bait vector served as the negative control.

To experimentally validate the predicted interaction between the candidate TFs and the *GhPAO21* promoter, we employed an integrated approach combining bioinformatic analysis and yeast one-hybrid (Y1H) assay. Initially, potential upstream regulators were identified through screening of predicted binding motifs against the JASPAR database (https://jaspar.elixir.no) and subsequent retrieval of their corresponding gene sequences from the CottonMD database, which yielded eight candidate TFs (Table S5). Subsequently, Y1H assays were then performed to individually assess the specific binding capacity of each candidate TF fused to the GAL4 activation domain to the MeJA-responsive *cis*-element (TGACG-motif) within the *GhPAO21* promoter. For the Y1H assay, the pLacZi reporter (bait) vector was constructed by inserting a *GhPAO21* promoter fragment containing the TGACG motif, whereas the pJG4-5 effector (prey) vector was constructed using the complete coding sequences of eight candidate TFs. The resulting constructs were co-transformed into yeast reporter strain. Following selection on synthetic defective medium (SD/-Trp/-Ura) and subsequent screening on plates supplemented with X-gal, only the yeast clones co-transformed with pJG4-5-*GhTGA1* and pLacZi-*GhPAO21* exhibited a distinct blue phenotype and robust growth, whereas clones harboring the remaining seven candidate TFs, as well as the empty vector controls, appeared white with no detectable color reaction (Figure 9). Collectively, these results demonstrate that of the eight candidate TFs tested, only *GhTGA1* exhibited direct and specific binding to the TGACG motif (MeJA response element) within the *GhPAO21* promoter, whereas the remaining seven displayed no detectable binding activity. Therefore, we hypothesize that *GhTGA1*, as a key component of the MeJA signaling pathway, mediates the regulatory effects of MeJA on cotton fiber development, at least in part, through the transcriptional expression of *GhPAO21*.

To further validate the regulatory relationship between the *GhPAO21* promoter and *GhTGA1*, we performed a dual-luciferase reporter assay. Briefly, the *GhPAO21* promoter was cloned into the pGreenII 0800-LUC reporter vector, and full-length CDS of *GhTGA1* was cloned into the pGreenII 62-SK effector vector, after which both constructs were introduced into *Agrobacterium transformation*. For transient expression assays in *Nicotiana benthamiana* leaves, the following four effector/reporter combinations were prepared: pGreenII 62-SK and pGreenII 0800-LUC, pGreenII 62-SK and pGreenII 0800-LUC-*pGhPAO21*, pGreenII 62-SK-*GhTGA1* and pGreenII 0800-LUC, and pGreenII 62-SK-*GhTGA1* and pGreenII 0800-LUC-*pGhPAO21*. Each combination was infiltrated into four distinct locations on the same leaf to minimize biological variation. Following infiltration and an appropriate incubation period, dual-luciferase (LUC/REN) activity assays were performed.

**Figure 10:**
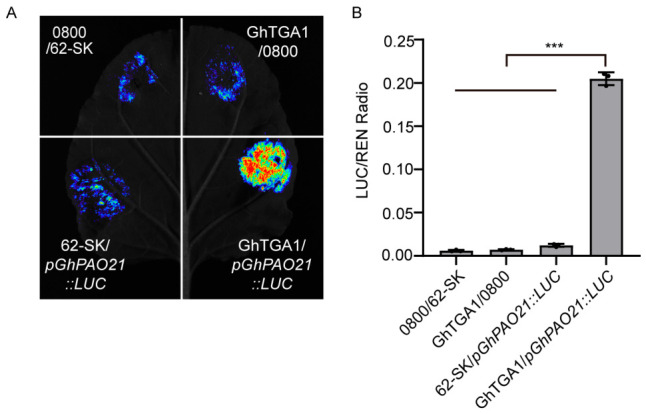
Experimental validation of the interaction between *GhPAO21* and *GhTGA1* through tobacco transient transformation. (**A**) Tobacco transient transformation experiments demonstrated that *GhTGA1* drives transcriptional activation of *GhPAO21*. The bioluminescence signal intensity is represented by color: blue indicates low transcriptional activation activity, while red indicates high activity. (**B**) Quantitative analysis of dual-luciferin activity assay results. Error bars represent standard deviations (n = 3). Statistical significance (*t*-test): *** *p* < 0.001.

As shown in Figure 10, co-expression of *GhTGA1* with *GhPAO21* promoter-driven LUC reporter resulted in a significant increase in the LUC/REN activity compared to all control groups, demonstrating that *GhTGA1* strongly activates the transcription of *GhPAO21*.

## 3. Discussion

The *PAO* gene family plays essential roles in plant growth, development and abiotic stress tolerance, and their biological functions have been systematically summarized in previous studies [30]. However, comprehensive genome-wide analyses of the *PAO* gene family in *G. hirsutum*, *G. arboreum* and *G. raimondii*—encompassing their evolutionary diversification and transcriptional regulatory mechanisms—have yet to be reported. Furthermore, the specific functional roles of these genes in cotton fiber development and their associated regulatory networks remain entirely unexplored.

This study presents a comprehensive genome identification of the *PAO* gene family in *G. hirsutum*, *G. arboreum* and *G. raimondii*, revealing its species-specific evolutionary characteristics by significant gene family expansion. Specifically, we identified 23 *PAO* family members in upland cotton, a number approximately four-fold greater than that observed in its diploid progenitors (five members each in *G. arboreum* and *G. raimondii*) (Figure 1 and Figure 4). The lineage-specific expansion, likely driven by tetraploidization, has established an evolutionary framework for the functional diversification of the *GhPAO* gene family.

Functionally, our expression profiling revealed that the transcriptional patterns of *GhPAO* genes are intimately associated with fiber development in striking contrast to the predominantly stress-responsive functions of *PAO* genes reported in other plant species. Notably, *GhPAO10* and *GhPAO21* exhibited pronounced transcript accumulation during the rapid fiber elongation phase (10 to 15 DPA), suggesting their critical involvement in regulating this developmental process (Figure 8). These genes are likely to exert positive regulatory effects during critical stages of fiber development by modulating polyamine catabolism and H_2_O_2_ signaling levels, thereby influencing turgor pressure maintenance, cytoskeletal dynamics, and cell wall extensibility within elongating fiber cells. Furthermore, the coordinated expression patterns observed between homologous gene pairs residing in the A and D sub-genomes of cotton species underscore the functional conservation and sub-functionalization that have accompanied polyploidization—a hallmark characteristic that distinguishes the *GhPAO* gene family from its counterparts in diploid plant species.

Under drought stress, *PAOs* are known to maintain intracellular polyamine homeostasis through the degradation of excess polyamines. In the present study, analysis of PEG-induced drought stress treatment revealed distinct expression kinetics of *GhPAO10* and *GhPAO21* (Figure 7). *GhPAO21* exhibited rapid induction at the early stress stage (3 h), followed by a subsequent decline, whereas *GhPAO10* displayed a relatively stable sustained expression profile (Figure 7). The pivotal role of PAOs in maintaining intracellular polyamine homeostasis and redox balance under drought conditions—achieved through the precise degradation of excess polyamines and dynamic regulation of H_2_O_2_ signaling [31,32]—provides a mechanistic framework for interpreting these distinct expression kinetics. Preliminary analysis of drought-responsive transcriptomic data further revealed functional differentiation characteristics among *GhPAO* family members under abiotic stress. Specifically, *GhPAO21* exhibited a rapid induction followed by rapid decline in expression pattern, which closely mirrors the drought-responsive behavior of PAO genes reported in alfalfa (*Medicago sativa*). Similar expression dynamics have been documented for *OsPAO2* and *OsPAO6* in rice (*Oryza sativa*) as well as *SbPAO5* and *SbPAO6* in sorghum (*Sorghum bicolor*), suggesting that early rapid induction of *PAO* genes may represent a conserved evolutionary strategy underlying plant adaptation to drought stress.

In contrast, *GhPAO10* exhibited a relatively stable expression profile, a pattern clearly distinct from the stress-induced, late-stage transcriptional activation reported for maize *ZmPAO6* and tomato *SlPAO4* [33,34]. Notably, within the tomato PAO family, individual members display highly divergent expression dynamics under stress conditions [35], suggesting that the distinct regulatory behaviors of *GhPAO10* and *GhPAO21* may reflect a broader evolutionary trend of functional specialization within the *GhPAO* gene family. We hypothesize that *GhPAO10* contributes to the drought stress response via sustained, basal-level polyamine catabolism—a mechanism that not only mitigates the cytotoxic effects of excessive polyamine accumulation but also preserves the spatiotemporal homeostasis of local H_2_O_2_ signaling. This model of regulation is particularly well suited to the physiological demands of sustaining fiber development and growth in cotton under prolonged drought conditions.

H_2_O_2_ generated via PAO-mediated polyamine catabolism serves as a pivotal signaling molecule that bridges primary metabolism and intracellular signal transduction [25,36,37]. For instance, *FsPAO1* and *FsPAO4* in forsythia (*Forsythia suspensa*) [38] are induced under drought stress, and their physiological functions rely on H_2_O_2_-mediated signaling cascades that activate downstream defense-related genes. In this study, we demonstrated that the promoter regions of *GhPAO* genes are enriched with stress response *cis*-elements, including LTR and MBS (Figure 6), further supporting their precise transcriptional regulation by drought-associated signals. We propose that members of the *GhPAO* family constitute a complex metabolic-signaling network through their spatiotemporally distinct expression patterns, thereby exerting differentiated regulatory functions across the various stages of drought response. Collectively, these findings substantially advance our understanding of the functional complexity inherent to the *GhPAO* gene family, offer novel mechanistic insights into the molecular basis of drought adaptation in cotton, and identify promising candidate genes for drought-resistant molecular breeding programs.

Our analysis revealed that the promoter regions of the *GhPAO* genes are enriched with hormone-responsive cis-elements, among which the methyl jasmonate (MeJA)-responsive element was particularly prominent. This enrichment is highly consistent with the well-established central regulatory role of the jasmonic acid (JA) signaling pathway in cotton fiber development [27,28]. Recent studies have further elucidated that the JA signaling pathway functions as a critical regulatory mechanism governing cotton fiber elongation. The JA signaling pathway is rapidly activated both under stress conditions and in response to exogenous MeJA treatment. Notably, low-concentration MeJA treatment of cotton ovules has been shown to significantly promote fiber elongation and increase final fiber length [39]. Previous work has also demonstrated the precise hormonal regulation of cotton fiber elongation [40,41]. Furthermore, key transcription factors within the JA signaling pathway have been shown to modulate cotton fiber development by directly binding to MeJA-responsive elements within the promoters of downstream target genes [42]. These findings provide a crucial theoretical framework for exploring the functional association between *GhPAO* genes, MeJA signaling, and fiber development. In this study, we demonstrated that *GhPAO21*, which is highly expressed during the fiber elongation stage, harbors a MeJA-responsive element (TGACG-motif) within its promoter region. Furthermore, Y1H assays confirmed that this promoter is specifically bound by *GhTGA1*, a key transcription factor in the JA signaling pathway (Figure 9). Dual-luciferase reporter assays further validated that *GhTGA1* transcriptionally activates the *GhPAO21* promoter (Figure 10), providing direct molecular evidence linking *GhPAO21* to MeJA-mediated signaling during fiber development. In conclusion, the specific binding of *GhTGA1* to the *GhPAO21* promoter not only defines the molecular basis of *GhPAO21* regulation via MeJA signaling but also establishes a preliminary regulatory framework—and identifies novel candidate targets—for dissecting the molecular mechanisms through which MeJA promotes cotton fiber development.

Based on the findings presented herein, future investigations should prioritize targeted functional validation in vivo, which remains an indispensable direction for advancing research in this field. First, chromatin immunoprecipitation sequencing (ChIP-seq) could be employed to systematically analyze the genome-wide binding landscape of *GhTGA1*, thereby elucidating the broader transcriptional regulatory network in which it operates. Second, the generation of transgenic cotton lines harboring *GhPAO21* overexpression constructs or CRISPR/Cas9-mediated knockout mutations would enable direct phenotypic assessment of fiber development, thereby definitively establishing the specific functional roles of *GhPAO21* in this process. Furthermore, the precise molecular mechanisms underlying the interplay between the *GhPAO* genes, phytohormone signaling, and other interconnected pathways warrant further in-depth investigation. Collectively, these endeavors will substantially refine our understanding of the regulation networks governing cotton growth and fiber development, thereby providing a more robust theoretical foundation and technical framework for the genetic improvement in fiber quality.

## 4. Materials and Methods

### 4.1. Identification of Cotton GhPAO Genes

The amino acid sequence of previously characterized *A. thaliana* PAO protein was retrieved from The Arabidopsis Information Resource (TAIR, available at https://www.arabidopsis.org/, accessed on 22 November 2025) and employed as the query sequence. TBtools (version 2.390) [43] BLASTp searches were conducted against the respective protein databases of the target cotton species. For *Gossypium hirsutum* (cv. TM-1), the TM-1_NBI genome assembly was utilized, with the corresponding protein sequences obtained from the Cotton Multiomics Database (CottonMD, available at https://yanglab.hzau.edu.cn/CottonMD, accessed on 23 November 2025). Additionally, the telomere-to-telomere (T2T) genome assembly for TM-1 (T2T-TM-1) was retrieved from the Cottonomics database (available at http://cotton.zju.edu.cn/, accessed on 2 March 2026). For *Gossypium arboreum*, the reference genome assembly version 1.0 (*Gossypium_arboreum*_v1.0) was employed, which is available under NCBI RefSeq accession: GCF_000612285.1. For *Gossypium raimondii*, genome assembly version 2.1 was utilized, corresponding to NCBI taxonomy ID: 29730. For the TBtools (version 2.390) [43] BLASTp searches, an E-value threshold of <1 × 10^−5^ was applied to filter significant hits. Additionally, we obtained the hidden Markov model (HMM) profile for the PAO domain (PF01593) [30]. This profile was from the Pfam database [44]. We used it to screen protein sequences using HMMER. The resulting candidate genes were further validated by confirming the presence of the characteristic PAO domain using SMART [45] and InterPro databases [46]. The full-length nucleotide sequences of all identified *GhPAO* genes family members are provided in File S1 (FASTA format) and Table S1.

### 4.2. Chromosomal Location and Collinearity

Chromosomal location information for the identified *GhPAO* genes was extracted from the corresponding GFF3 genome files of the respective cotton species. Gene chromosomal distribution maps were generated, and synteny/collinearity analyses were performed using TBtools (version 2.390) [43].

### 4.3. Phylogenetic Analysis

Phylogenetic analyses were performed using MEGA (version 11.0). For the reconstruction of the phylogenetic tree, the Maximum Likelihood (ML) algorithm was selected. Branch support was assessed via bootstrap analysis with 1000 replicates. All remaining parameters were retained at their default settings.

### 4.4. Gene Structure and Cis-Element Analysis

Conserved protein motifs were identified using the MEME Suite (version 5.5.8) [47]. *cis*-acting regulatory elements within the 2000 bp promoter regions upstream of the translation start site were predicted using PlantCARE (available at https://bioinformatics.psb.ugent.be/webtools/plantcare/html/, accessed on 2 December 2025). The resulting *cis*-element distributions were visualized using TBtools software (version 2.390) [43].

### 4.5. Expression Pattern Analysis

Transcriptome datasets encompassing various cotton tissues and fiber developmental stages were utilized to analyze expression profiles of the *GhPAO* gene family, as detailed in the Data Availability Statement. Expression heatmaps were generated using TBtools software (version 2.390) based on Log_2_-transformed expression values [43].

### 4.6. qRT-PCR Analysis

Total RNA was isolated from the ovules and fibers of the cotton cultivar Jin668 at various DPA using the FastPure Universal Plant Total RNA Isolation Kit (Vazyme Biotechnology, Nanjing, China). Subsequently, first-strand cDNA was synthesized from 1 µg of the extracted total RNA using the HiScript II Q RT SuperMix for qPCR (Vazyme Biotechnology), strictly adhering to the manufacturer’s provided protocol. qRT-PCR was performed using the ChamQ SYBR qPCR Master Mix (Vazyme Biotechnology). Three independent biological replicates were analyzed for each gene under investigation. The cotton ubiquitin gene *UBQ7* (GenBank accession no: AY189972) served as the internal reference for normalization of the expression data. The primer sequences utilized in this study are detailed in Table S3. Relative gene expression levels were calculated using the 2^−ΔΔCT^ method, as previously described [48].

### 4.7. Y1H Assay

In the Y1H assay, an approximately 2000 bp promoter fragment of *GhPAO21* was cloned into the pLacZi bait vector (Clontech, Mountain View, CA, USA) to generate the reporter construct. The coding sequences of candidate TFs predicted to regulate *GhPAO21* were inserted into the pJG4-5 prey vector. The resulting prey constructs were individually co-transformed with the *GhPAO21* promoter reporter construct into the yeast strain EGY48. Positive transformants were selected on synthetic dropout medium lacking tryptophan and uracil (SD/-Trp/-Ura) and subsequently screened on X-gal-containing agar plates to assess β-galactosidase activity. The cultures were incubated at 30 °C for 3 to 5 days, and the development of blue coloration was monitored to evaluate the binding capacity of the candidate TFs to the *GhPAO21* promoter. Primer sequences are provided in Table S3.

### 4.8. Dual-Luciferase Reporter Assay

The *GhPAO21* promoter fragment was cloned into the pGreenII0800-LUC reporter vector, and the coding sequence of *GhTGA1* was inserted into the pGreenII62-SK vector (both vectors were available from our laboratory). The primer sequence information is shown in Table S3. Following transformation into *Agrobacterium tumefaciens* strain GV3101, the recombinant strains were cultured, harvested by centrifugation, and resuspended in infiltration buffer to a final optical density at 600 nm (OD_600_) of 0.8–1.2. Equal volumes of the *Agrobacterium* suspensions harboring the reporter and effector constructs were mixed and co-infiltrated into the abaxial surface of *Nicotiana benthamiana* leaves. Following infiltration, plants were initially maintained in darkness for 12 h and subsequently grown under normal light conditions for 2 to 3 days. Firefly luciferase (LUC) activity was visualized by applying D-luciferin substrate and capturing luminescence signals using a live imaging system. For quantitative analysis, the infiltrated leaf tissues were harvested, ground in liquid nitrogen, and homogenized in the appropriate lysis buffer. Sample preparation and measurement were performed using the TransDetect Double-Luciferase Reporter Assay Kit (TransGen Biotech, Beijing, China; catalog no. FR201-02-V2) strictly following the manufacturer’s instructions. Firefly luciferase (LUC) and Renilla luciferase (REN) activities were sequentially quantified using a multifunctional microplate reader. The regulatory effect of *GhTGA1* on the *GhPAO21* promoter was evaluated by calculating the ratio of LUC activity to REN (LUC/REN).

## 5. Conclusions

This study has provided the first comprehensive genome-wide characterization and functional analysis of the polyamine oxidase (PAO) gene family in cotton. Our results revealed that the *GhPAO* family underwent a significant expansion event during cotton evolution and that its members exhibit distinct stage-specific expression profiles during fiber development, a process potentially modulated by phytohormones signaling. These findings substantially advance our understanding of PAO-mediated regulatory mechanisms in cotton fiber development. Notably, we identified *GhTGA1* as a key upstream TF that binds directly to the MeJA-responsive *cis*-element within *GhPAO21* promoters, thereby preliminarily implicating this regulation of module in control of fiber initiation and elongation. The study establishes a foundation for future functional investigations and highlights *GhPAO21* as a prime candidate for subsequent genetic validation—through overexpression, CRISPR/Cas9-mediated knockout, or gene silencing approaches—to definitively elucidate its mechanistic role in fiber development. Further investigations should explore the potential contributions of *GhPAO* family members to abiotic stress adaptation and dissect the regulatory networks governed by MeJA signaling during fiber morphogenesis.

## Figures and Tables

**Table 1:** Information and physicochemical characteristics of PAO genes in *Gossypium hirsutum* L.

Gene	Gene ID	Protein			Instability Index	GRAVY	Subcellular Localization Prediction
		Length (aa)	MW (kDa)	pI			
*GhPAO1*	Gh_A03G0717	1762	192.50	5.74	41.42	−0.48	Nucleus
*GhPAO2*	Gh_A05G0221	561	61.33	5.8	37.48	−0.33	Chloroplast
*GhPAO3*	Gh_A05G1548	778	85.58	5.81	43.73	−0.25	Cytoplasm
*GhPAO4*	Gh_A05G2520	854	93.52	8.72	38.18	−0.23	Cytoplasm
*GhPAO5*	Gh_A05G3233	1807	197.81	5.86	42.50	−0.54	Nucleus
*GhPAO6*	Gh_A07G0104	508	56.67	5.91	36.29	−0.22	Chloroplast
*GhPAO7*	Gh_A08G0331	499	55.95	5.55	27.75	0.02	Endoplasmic Reticulum
*GhPAO8*	Gh_A08G0507	505	56.12	5.37	33.18	−0.14	Extracellular Localization
*GhPAO9*	Gh_A08G1292	493	55.54	5.25	33.29	−0.26	Extracellular Localization
*GhPAO10*	Gh_A12G2582	488	54.02	5.68	40.09	−0.01	Plasma Membrane
*GhPAO11*	Gh_A13G1224	750	83.42	8.56	42.13	−0.19	Cytoplasm
*GhPAO12*	Gh_D02G0971	1770	193.37	5.77	41.81	−0.48	Nucleus
*GhPAO13*	Gh_D04G0374	1608	176.07	5.53	41.52	−0.51	Plasma Membrane
*GhPAO14*	Gh_D05G0300	560	61.30	5.76	37.7	−0.32	Cytoplasm
*GhPAO15*	Gh_D05G1723	778	85.49	5.90	42.90	−0.24	Cytoplasm
*GhPAO16*	Gh_D06G1841	907	99.02	8.77	38.36	−0.23	Cytoplasm
*GhPAO17*	Gh_D07G2378	509	56.89	5.77	37.79	−0.23	Cytoplasm
*GhPAO18*	Gh_D08G0428	536	60.02	5.52	26.16	0.03	Endoplasmic Reticulum
*GhPAO19*	Gh_D08G0594	505	55.97	5.35	33.02	−0.12	Endoplasmic Reticulum
*GhPAO20*	Gh_D08G1583	493	55.39	5.31	33.86	−0.25	Endoplasmic Reticulum
*GhPAO21*	Gh_D12G0881	488	54.10	5.55	40.28	−0.03	Plasma Membrane
*GhPAO22*	Gh_D13G1522	750	83.40	8.76	41.80	−0.18	Cytoplasm
*GhPAO23*	Gh_Sca005492G01	910	99.42	8.90	38.95	−0.25	Cytoplasm

Length, amino acid sequence length; MW, molecular weight; pI, isoelectric point; GRAVY, grand average of hydropathicity.

## Data Availability

The datasets generated and/or analyzed during the current study are available in the Supplementary Information Files repository. The RNA sequencing data of cotton under stress conditions used in this study were obtained from the National Center for Biotechnology Information (NCBI), https://static.pubmed.gov/portal/portal.fcgi/, accessed on 15 January 2026, with project number: PRJNA248163. The transcriptome data of different cotton tissues and fiber development stages were obtained from the NCBI project number: PRJNA490626. The complete nucleotide sequences of all gene members analyzed in this study are included within the Supplementary Materials of this article.

## Supplementary Materials

The following supporting information can be downloaded at: https://www.mdpi.com/article/10.3390/plants15101429/s1, Figure S1: The conserved motif of GrPAO proteins. Figure S2. The conserved motif of GaPAO proteins. Figure S3. Expression of *GhPAO10* and *GhPAO21* genes at different stages of cotton development. Expression of *GhPAO10* and *GhPAO21* is normalized based on the expression of *GhUBQ7*. Error bars represent ± SD (n = 3). Asterisks indicate significant differences by *t*-test; ** *p* ≤ 0.01, *** *p* ≤ 0.001. The complete nucleotide sequences of 23 *GhPAO* genes analyzed in FASTA format are provided in File S1 and listed in Table S1. Table S1 Summary of Nucleotide Sequence information for *GhPAOs*. Table S2: This table shows the ID correspondence of polyamine oxidase (PAO) family genes between the gap-free telomere-to-telomere (T2T) genome and the previous TM-1-NBI genome of *Gossypium hirsutum.* Table S3: Primers used in this study. Table S4: List of the conserved motifs of the GhPAO proteins; Table S5. Information on candidate upstream regulatory factors targeting the TGACG motif in the *GhPAO21* promoter.
