# Supplementary material for: Evolution and Expression Analysis of PAO Gene Family in Cotton: Focusing on Fiber Development and Stress Response
Source: Plants (Basel). 2026 May 7;15(10):1429. doi: 10.3390/plants15101429 (PMC13210522; doi:10.3390/plants15101429)
Supplement: Supplementary file 1 [file plants-15-01429-s001.zip › Supplementary Materials Table S1.pdf]

Table S1 Summary of Nucleotide Sequence information for *GhPAOs*

Gene Name	Nucleotide Sequence
GhPAO1	ATGGATGGTGGTGGGGGCTCTAAGAAAAGATTAAAGGTCACAGCGGCGGAGGTTGGTGTTGATTCAGATGATGA<br>TGAGCCCATTTTGTCTTTGTTGAAGTTAAGGAAACCTAAGAATCCTAAAAAGGATAAGGCTGGATTGGAAGGCA<br>GTGCCGGGAAGTGCCAGAAGGTTGAAGTTAAAGCAGGTAAAACTGTGGATAAAGATGAGGAGGATTTTGGGGG<br>AATGAATGATACATTGGCCAGCTTCAGAAAGAAGTTAAAGGATCCTAAAAAAGATATTGATCCAGGAGCAATGA<br>GGGTAAGGAGTTATTCTTTGAATAAGTCTGTGGAGGGTGGTGGAATTTTGGATGGGAAATCTGTGTCGAACACTG<br>ATGTGAAAGGTCAGGATATTGGTGAAGACAGGTCTGATGTGGCTAATGATAAAGTTGTTGGAAAAAAGCGTACA<br>GGGAAAGTAAGGAGAACCAAGTCAGATTCAAAAGCCACGCCTAGTGAGGTTGATGATGAATCTGGAGCTAAAC<br>TTGAGGAAGATCAGAATGAGGGAGGTTTGTTGCCTGGGGAAGGTTCGTATCAGTGTTCTCACAAGGCACAATCT<br>GGTTCAGTAGGGAAATCTTGCGCAATTTTGAGTTTGAAACATAATTGCGAGGCTGCTCATCATGCTTCTGATTCAA<br>AGAATCCTAGCAGAAATTATGGTGATAGTTCTCATTCAGTTTCTAGCTCAAGCTTCTCACATTCATCCTCCAAAGA<br>ATGCAACACAGCTGAGAATCAAGGATTTGACCATAGTTTGTGTCAACAAGAAAGCATTTTGGAACCAGGTGACG<br>TAACTGTTCAAAAGGATCCTACAGAGCATCCATGTAGGTCATCTAAATTTTGTGACAAGGAAAGATATTGTCATTC<br>CAACATTGAGCTCAGGGACAATTTCTCAGCAATTGACCAGAGGAGTAGACCAGGAAGTGAAAGTTCACAACAA<br>AATAAACATAATCTGTCACTGTCTGTTGTTGATTCACTGAATATGGAAGAAACTTGATGTTCCAAATTCTTGCGCT<br>GAGGAATACTCTTTAGAAACTTCCATTCATCCCAATGAACTTGTTGCCTCCATTCAGAGGTGCAACTCTGCTCTGG<br>ATCAACCTTCTGAAGACGCAAGCCATGGTGCTTGTGGTCCCAGCCATGATACTGTTTTCATCAGCAAAGAGGCCA<br>ATGTTGACTCTCCCATTTCAACACCTGATGAAAATGAAAGTTTCCATGAAGATGCAGTCTCTCTCCCTAGTTCAGA<br>AATAAGAAACAGCAAGTCATCAGCTGTCCAGCGAGGTGGGCGCAATATTAAAAAGCGTAGACACGGAGATATG<br>GCTTATGAAGGGGATGCTGATTGGGAAAATTTGCTAACCGAGCAAGGTTTTTTTGGAAATCAACAGTTTGTAGAC<br>AGTGATCATTCCTTTAGAGCAAGAGAGAAGTTTGATGAGGCAGCAGTATCATCTGGACTGAAAGCTCGTGCTGTG<br>GGGCCAGTTGAGAAGATCAAATTTAAGGAGGTGTTGAAGTGTAGAGGTGGGCTACAGGAATACTTGGAATGCAG<br>GAATCATATTTTAGGTCTTTGGAGCAAAGATGTTAACCGCATTTTGCCTCTTGTTGACTGTGGTGTTAGTGACACTC<br>CTTCAGAGGGTGAACCATCCCGAGCTTCTCTAATTAGGAAGATATATGCATTTCTTGATCAGGGTGGTTACATAAA<br>CTTTGGAATTTCTTCAAAGAAAGAGAAGGCTGAGCTTAGTGTTAAGGACAACTACAAACTTCTTGAGGGAAGAA<br>GAAGTGATGGTAATTCTGTTGCCTCTGTTGCTGATTCAGAGGATGGAGTTGCCTTTATCCTGGGTCAGGTCAAGAA<br>TTCTAAAGCTTCTATGGATGCAAAGACTGGTGTTAGAGTTGTTGATGAAAACCAGGCATCTGAAGCCACAATAGC<br>AATAGCTGAAGTATTGGTTGATTCGATCACACCAAAATTACCTTATATATGCCAGCAAAATAGCAGCGTTAGTGCA<br>AAATTAAACACTGGATTGATTAGTTCGCAGGTTTCAAGTACTGATCTATCTTGTGATGCAATTGATGTTGGAGTAG<br>CCCCTGTAGTAACTCCAGAAGAAAGGAATGACTCACAGTATGTTCAATCTGCAACTTATGATAAACCTGACGGGA<br>ATCATCAGCTGCTGAATGATTCAGAGGTCAGAAAGAACATCATAGTTATTGGAGCTGGTCCTGCTGGATTGACTG<br>CTGCACGCCACTTGAAACGTCAGGGATTTTCTGTAGTTGTACTTGAGGCTAGGGATAGGATAGGAGGTCGTGTTT<br>ATACTGATTGCTCCTCTCTTTCAGTACCTGTGGATCTTGGGGCTAGCATTATTACTGGAGTTGAGGCTGATGTGTCA<br>ACTAATAGAAGACCAGATCCATCCTCATTGATTTGTGCACAGTTGGGGCTAGAGTTAACTGTGTTGAATAGTTCCT<br>GTCCTCTTTATGACATTGTATCTGGTCAAAAGGTTCCTGCTGATCTGGATGATGCTCTGGAAGCTGAATACAATAG<br>TCTTCTTGACGATATGGTGTTTCTTTTTGCTCAAAAAGGTAAAAAAGCAATGACAATTTCTCTTGAGGATGGTTTA<br>GAATATGCCCTAAAAAGGCATCGGATGGAAGAAATAGGAGTAGATATTGAAGACAGAATCACATTCTTCAGTGG<br>ATGCTTTCTATGACTCAAAAGAAAGCAATATCTTTGGATTTCCTGGAAAAAAACGTTCTGAAGAGGAGATTTTGA<br>GTCCTCTTGAGAGAAGGGTCATGAATTGGCACTATGCTCACTTGGAGTATGGCTGTGCTGCTTCGCTTAAGGAAG<br>TTTCTCTTCCCAACTGGAATCAGGATGATGTTTATGGCGGCTTTGGAGGAGCCCATTGTATGATTAAAGGGGGTTA<br>CAGTACGGTGGTTGAGTCTCTTGGAGAAGGGCTTCTGATCCACTTGAACCACGTAGTCACAAATATTTCATACAG<br>CCCAAAGGGCCCGGGGGTTGATAATAGTCATCATAGGCAGGTCAAAGTTTCCACATCAAATGGCAGTGAGTTTTC<br>AGGAGATGCTGTGCTTATCACTGTGCCACTTGGTTGCTTGAAAGCAGGAGCCATAAAGTTTTCTCCTCCGTTGCCC

	CAATGGAAACATTCTTCCATACAGCAGCTTGGTTTTGGAGTACTTAATAAAGTCGTCTTGGAATTCCCAGAAGTTT<br>TTTGGGATGATACTGTGGATTACTTTGGAGTGACTGCTGAGGAAACAGATAGTAGAGGCCATTGTTTTATGTTTTG<br>GAATGTCCGAAAAACTGTTGGGGCTCCTGTTCTTATAGCCTTAGTGGCTGGTAAGGCAGCTATTGATGGTCAAAAT<br>ATGAGCGCATCAGATCATGTAAACCATGCTGTAATTATTCTCCGTAAACTTTTTGGAGAGGCTTCAGTTCCTGATC<br>CTGTTGCCTCAGTTGTTACTGATTGGGGAAGGGATCCTTTTAGTTATGGCGCTTACTCCTATGTTGCCATAGGAGCA<br>TCTGGAGAAGACTATGATATGTTGGCCAGGCCTGTTGAGAACTGCTTGTTTTTTGCTGGAGAAGCTACCTGCAAG<br>GAGCATCCTGACACAGTTGGTGGTGCAATGTTGAGTGGGCTTCGGGAGGCTGTGCGATTAATTGACATATTTACC<br>ACTGGAAATGATTATACAGGAAGTAGAGGCAATGGAGGCTGCCCAGAGACATTCAGAATCAGGAAGGGATGAA<br>GTTAGGGACATAATTAAGAGACTTGAAGCAGTTGAACTTTCTAATGTCTTGTACAAAAACTCTTTGGATCGTGCTC<br>GGGTTTTAAGCAGGGAAGCTTTACTGCGGGACATGTTCTTTAATGTGAAAACCACTGCAGGACGATTGCATCTAG<br>CCAAGAAGTTGTTAGGTCTCCCAGTTGAATCCTTGAAATCCTTTGCTGGGACAAAGGAAGGGCTTAGCACACTC<br>AACTCATGGATGCTGGATTCAATGGGGAAAGATGGGACGCAACTGTTGCGCCATTGTGTTCGTCTTCTTGTGCTTG<br>TCTCAACTGATCTACTTGCAGTTCGTTCATCAGGCATAGGGAAAACTGTGAAGGAAAAAATTTGTGTGCATACAA<br>GTCGTGATATACGTGCTATAGCAAGCCAGCTGGTTAATGTTTGGCTTGAAGTCTTCCGTAAGGCAAAAGTTTCTTC<br>AAAGAGAAAATCCCTTAAAGATCCAGCTTCAGGAAAGCCACCTCTGCACTCACAGCATGGTGCTTTTGAGAGTA<br>AAGAAAGCTTGCGGATCCATTTTCTGCTGGAAAGCAGTATCCTTTAAACATAAAGGAGAATGGCAAATCACTTGA<br>TATTGAGGTGGAAGCTGTCAACCAAGGAATGTCAGAGGAAGAGCAGGCTGCCTTTGCTGCTGAAGCAGCTGCCC<br>GAGCTGCAGCAAAAGCAGCTGCAGAGGCACTTGCATCCACAGAAGCCAATTGCAACAAATTACTGCAGCTTCCT<br>AAGATTCCTTCTTTTCACAAATTTGCCAGAAGGGAGCAATACGCACAAATGGATGAAGGGAAATGGCCTGGTAG<br>TGTTTTTGGAAGACAAGATTGTATATCAGAAATTGACTCTAGGAACTGCAGAGTCAGGGACTGGTCTGTTGATTTC<br>TCTGCTGCTTGTGTTAACCTTGACAATTCCGGAATGTCAGTAGATAACCTGTCTCAGAGGAGCCACTTGAGCTCA<br>GAGAACATTCTGGAGAAAGTTTGGCTGTGGATAGCAGTATCTTCACGAAAGCATGGATTGATACTGCTGGTAATG<br>GGGGGATCAAGGATTATCATGCCATTGAGAGATGGCAGTCTCAAGCAGCTGCTGCTGATCCAGATTTCTTCCATC<br>CTACAAATTTCAAGGATGAGGAAGATTCAAATACGAGTTCAAGGCAACCAACCTGGAAGAATGATGGACGAGC<br>AAATGAGAGCTCCGTCTCCCAAGTTTCTGTAAACAAGGAGCGATTTGAAAATCATCCCTGTGGAGCTGATCGTAT<br>TAAACAGGCTGTCGTTGATTATGTTGCATCATTGCTAATGCCCCTTTATAAGGCAAGAAAAATTGATAAGGAGGGA<br>TACAAATCGATAATGAAAAAAACTGCAACAAAGGTAATGGAGCAGGCAACAGATGCAGAGAAAAACATGGCT<br>GTTTTTGAATTTCTTGATTTCAAGCGCAAAAACAAGATTCGTCCCTTTGTAGACAAATTGATTGAGAGGCACATGG<br>CAATGAAGCCAACCATGAATCTATGA
GhPAO2	ATGGGAAAGAAGCCAAGAATTGTGATAATTGGAGCTGGGATGGCTGGTCTTACTGCAGCTAACAAGCTCTATACT<br>TCCACTGGCTCCGACCATTTGTTTGAGCTTGTTGTTGTTGAAGGTGGTGATAGAATTGGTGGCAGAATCAACACTT<br>CGGAGTTTTGTGGTGACAGAATTGAGATGGGTGCTACTTGGATCCATGGTATAGGAGGCAGCCCGGTACATCAAA<br>TTGCTCGGGAAATCCATGCGCTTGAGTCTGATAAGCCATGGGAGTGTATGGATGGGTTCTCGGGTGAGCCAAAGA<br>CTATTGCTGAAGGTGGGTTCGAGCTAAATGCCTCCATCGTTGACCCCATATCCACCCTTTTCAAAAACCTGATGGA<br>TTTCGCTCAAGGGAAGCTGACTGAATACAGTGCAGGCAGCGGAGGAGATGCTTGTTACTACAATTTTGCAGCTAA<br>AGCAGCCTTGAAAGATTGTACGAGCAATGGTGGCTTTGGTAACCAGAGTAGTGTCGGTGCGTTTCTTAGACGAGG<br>CCTTGGTGCTTACTGGGATTCTTGCAAGGACCGTGAGGAGCTGAACGGATATGGTAAATGGAGCAGAAAATTGC<br>TTGAAGAAGCCGTTTTTGCTATGCATGAAAACACCCAGAGAACTTATACTTCTGCCGGTGATCTGTTCAATCTAGA<br>TTACGAGGCAGAAAGCGAGTACCGTATGTTTCCTGGTGAAGAAATCACCATTTCTAAAGGCTATTTGAGCATAAT<br>TGAACATCTTGCATCTGTTCTTCCTCCTGGCGTAATCCAATTAGGCCGCAAAGTCACAAGAATCGAATGGCAACC<br>TGAGGGTCATAAATCAATACAAGTTCCAAATGGCTATGATTCCAGACCAGTGAAGATTGAGTTTTGTGATGGATCT<br>TCTATGTTAGCAGATCATGTGATAGTCACAGTTTCATTAGGGGTCTTAAAATCTGGAACTGGTCAAGATTCGGGTA<br>TGTTCAATCCTCCCCTTCCTACTTTCAAGACAGAGGCTATATCAAGACTTGGATATGGAGTTGTTAACAAGCTGTT<br>CCTTCAATGGAGTCCAAATGGTAATCGACCCGCCAATGATAAAGAGAAGTTCCCTTCCTTGCAAATTGTTTTCCAT

	CCCCCAGAATCCGAGTTAAGGCATGAAAAGATCCCAGGGTGGATGAGGAGGACAGCTTCACTGTCTCCTATTTAT<br>AACAATTCAAGCGTCCTCCTATCCTGGTTTGCAGGTAAAGAAGCACTTGAGCTTGAAACACTTAGCGATGAAGA<br>GATTATAAATGGAGTTTCAGCAACAGTATCTGGTTTATTACCAGTATCAAAACACCACAAGGAAGACAAGTATAA<br>TTCCCCTGAATTCTGCAATGGGAATGTGGAGAGCTGTGATGACAATGGAGTGAGATTTGGTAAGGTTTTGAAGAG<br>CAAATGGGGCAGTGATCCATTATTCTTGGGATCTTACAGCTACGTGGCTGTTGGATCAAGCGGTGCTGATTTAGAC<br>ACAATGGCTGAACCCTTACCAAAGCTTGGGAGCACTGACTCAGACCACCATCCACTTCAAATATTGTTTGCTGGG<br>GAGGCTACACACAGAACCCACTATTCTACAACCCATGGAGCTTATTTCAGTGGTCTTAGGGAAGCCAATAGGCTT<br>CTCAAACATTATCGTTGTGTTGGGGTTTAG
GhPAO3	ATGGAGCCTCCCCAAGATACTTCCGAGAACCCTAACGATGTCCTTTCCGACGACGACTCTTCACCGGAAAACAC<br>CAATCCCGACGATCAAGAAATCCCCAGTACGACACTCGACCCACCCATTTCCGATACCCAAGATGAATCCTCCG<br>ATCCCGTCCCCGACGAGCAACCCGAAAACACTAATTCGAACCCCGCCGAGCCTGGTCCACCTGCACGCAAGCG<br>CCGCCGCAGAAAGCGTTTCTTTACAGAACTCATCGCCAATCCATCCTTCTCCAAGAACCGGCGCCCTAGAATATC<br>GGGCCTAGCTAGAGAAATGGACACCGAAGCTTTAATCGCGATCTCCGTTGGTTTCCCTGTTGATTCTCTTACCGAA<br>GAAGAAATCGAAGCCAACGTGGTGTCCAGAATCGGAGGCCAAGAGCAAGCCAACTACATCGTTGTAAGAAATC<br>ACATTCTGGCTCGCTGGAGATCCAATGTATCCGTCTGGCTGACGCGCGAGCACGCCCTCGAATCAATCCGAGCTG<br>AGCACAAGAACCTAGTGAACGCAGCATACAATTTCCTTCTCGAACACGGTTACATTAATTTCGGTTTAGCCCCGG<br>CTGTTAAAGAAGCGAAATTGAAGTCTTTTGATGGTGTAGAAAGAGCCAATGTGGTGATTGTGGGTGCGGGTCTTT<br>CCGGTTTGGTCGCGGCGAGGCAATTAGTTTCCATGGGGTTTAAAGTTGTCATCTTGGAAGGTAGGACGCGCCCTG<br>GAGGGCGCGTAAAGACAAGGAAGATGAAAGGTGATGGGGTGGTGGCTGCAGCGGATCTTGGTGGGAGTGTTCT<br>CACGGGAATAAATGGAAATCCACTTGGGGTTCTTGCAAGGCAAATGGGATTACCGCTTCATAAGGTGCGAGATAT<br>TTGTCCTTTGTATTTGCCAGATGGAAAGGCCGTAGATGCTGATGTTGATTCTAGGATAGAGGTTTCATTTAATAAGC<br>TATTGGATAGGGTTTGTAAACTTAGGCATTCTATGATTGAGGAAGTTAAATCAGTTGATGTTCCATTAGGGACAGC<br>ATTAGAAGCCTTTAGGAGTGTTTACAAGTTTGCTGAGGATTCACAGGAGAGCATGTTGTTGAATTGGCATCTTGCT<br>AACCTTGAATATGCTAATGCTTCTTTGATGGCTAATTTGTCTATGGCCTATTGGGATCAAGATGATCCATATGAGAT<br>GGGCGGCGATCACTGTTTCATACCCGGTGGCAATGAGAGGTTTGTTCGAGCACTTGCGGAGGACCTTCCCATTTT<br>CTATGGGAGGACTGTGCAGAGTATCAGGTATGGTATCGATGGTGTTAGGGTTTACGCCGGTGGGCAGGAGTTTTG<br>TGGGGATATGGCTCTTTGCACTGTTCCATTAGGAGTTCTCAAGAAGGGATCGATAGAATTTGTTCCTGAGCTTCCG<br>CAAAGAAAGAAGGATGCCATTCAGAGACTGGGATTTGGGTTGCTGAATAAGGTTGCTATGTTGTTTCCATACAAT<br>TTTTGGGGCGGAGAGATTGATACTTTTGGCCATCTGACAGAAGACCCAAGTATGAGAGGCGAGTTCTTTTTGTTTT<br>ATAGCTATTCTTCTGTGTCAGGTGGTCCACTCCTTGTTGCTCTAGTTGCCGGAGATGCAGCAATCAAGTTTGAACT<br>GATGTCTCCTGTTGAGTCTGTGGAAAGGGTTTTAAACATATTGCGAGGCATTTTTCATCCAAAAGGGATTGTTGTA<br>CCAGATCCTGTCCAGGCTGTTTGTACCCGGTGGGGAAAGGATCGCTTCACTTATGGATCCTACTCTCATGTTGCTA<br>TTGGTTCATCCGGGGATGATTATGATATTCTAGCTGAGAGTGTTGGAGATGGGAGAGTCTTCTTTGCTGGTGAGGC<br>AACTAATAAGCAGTATCCTGCCACAATGCATGGAGCCTTTTTAAGTGGCATGAGAGAGGCCGCTAACATGCTTAG<br>AGTGGCCAGGAGGAGGTCATTGATTCTATCTGACAAAGTTAATAACGACTTGGAGAAATGTGATACTTTGAATAA<br>GTTGTTCGAGAACCCTGACCTGACATTCGGGAGCTTCTCAGCTTTGTTTGATCCCCATTCTAATGATGTTGGATCG<br>CATGCATTTATAAGGGTCAAATTCCATGGGGATAAATTAAACTCGAGTCACTTGTGTCTTTATGGCTTGATTACGA<br>AGAAGCAAGCCATTCAGTTAAGTGAAATGAATGGAGATGGGAACAGGATGAATTCGTTGCATCGTGACTTTGGG<br>GTGAAGTTGGTTGGTGGTAAAGGGTTATCAAATGTTGCGGAGTTGCTGATATCACGCATCAAAGCAGCTAAACCA<br>ACCTAA
GhPAO4	ATGAATCCACCAAATGAAACCCTGGATGATTTCTCTCAATTCCCTCTTCCCCATTTTACTCTCACTCCTCCCTTACC<br>AAATCCTACCTCTATTCCTAATTTCCATCCAATCCCAATCCTTAACCCGAATGCCGCTCCTCCTCTCAATGATCATC<br>TAATTTCCTTTCCAACCCCCAAAAAACGACGACGCGGCCGACCTCAACGCAGTGGCGCGACGTCGGCGTATCAG<br>TTCCTTACCTTCACCAATGGCTCCTTCAGCCCCAACCTCCCGAACTCTAATCCTAACCTTGACCTTAATTCAATAA

CCTCATCATCAGCGGCGACGGAACAAACTACACAACCCAAAATTGACGACGAGATCATTTTGATCAGTAAGGAA
TCAACGGCAGAGGCTCTCACCGCTCTTTCTGCTGGATTCCCTGCAGATTCCCTCACCGAGGAAGAAATTGATTTC
GGCGTCATTTCCTCCATTGGTGGCATCGAGCAGGTAAATTATATTCTCATTCGAAATCACATTATTGCAAAATGGC
GCGAAAATGCATCCAATTGGGTGGCTAAAGACATGCTTGTTGATTCTATACCGAAACATTGTAGCACGATTTTAAA
TTCTGCATATAATTATTTAGTTACGTATGGATATATAAATTTTGGGATTGCCCCTGCAATTAAGGAAAAAATTCCTGC
GGAACCGACTAGAAGTAATGTGGTTATTATTGGTACCGGGTTAGCAGGACTGGCTGCGGCTAGACAGTTAATGAG
GTTTGGGTTTAAGGTGACGGTTCTGGAAGGGAGGAAACGAGCCGGCGGGAGGGTTTATACAAAGAAGATGGAG
GGAGGGAATAGGGTGAGTGCAGCTGCTGATTTAGGTGGGAGTGTGTTAACAGGTACCTTGGGGAATCCGTTAGG
GATTCTGGCGAAACAATTGGGTTCTTCTCTTTTTAAGGTGAGGGATAAATGTCCACTTTACAGGACAGATGGGAG
TCCGGTGGATCCGGATATGGATATGAAGGTGGAGATGGCCTTTAATAGGCTTTTGGATAAAGCTAGCGAGCTTAG
GCAGTTGATGGGGGAGGTTTCTACGGATGTTTCACTTGGGGCTGCATTAGAGACATTTAGAGAGGTTTATAGGGAT
GCAGTGACTGAAGAAGAGATTAATTTGTTCAATTGGCATCTTGCAAATTTAGAATATGCAAATGCCGGATTGGTTT
CAAAGCTTTCACTTGCATTCTGGGACCAAGATGATCCATATGATATGGGAGGGGATCATTGTTTCTTGCCTGGAGG
AAATGGAAGGTTGGTTCAAGCTCTGGCCGAGAATGTGCCTATTTTGTACGAAAAAACTGTGCATACTATCAATTAT
GGCAATGATGGAGTGCAGGTTATGACAGGAAGTCAGGTGTATGAAGGTGATATGGCATTATGTACGGTACCTCTC
GGAGTTCTAAAGAGTGGGTCAATCAAGTTTGTCCCAGAGTTGCCTCAGAGGAAGCTTGATGGAATAAAGAGGTT
GGGATTTGGGTTATTGAACAAGGTTGGAATGCTTTTTCCTTATGTATTTTGGGGTACAGACTTTGATACCTTTGGGC
ATCTTACTAATGATCCAAGCCGTCGAGGGGAGTTTTTCTGTTCTATAGCTATGCAACAGTTTCCGGTGGTCCTCTA
TTGCTTGCTTTAGTAGCAGGAGAAGCTGCACACAGGTTTGAGAGTCTGCCTCCTATAGATGTTGTGGCCCAGGTT
CTCCAAATTCTCAAGGGTATATATGAACCACAGGGTATCACTGTCCCCGAGCCCCTCCAAACTGTCTGTACCAGA
TGGGGTGGTGATCCCTTCAGCCTAGGTTCATACTCTAATGTTGCAGTGGGAGCATCCGGGGATGACTATGATATAT
TAGCTGAAAGTGTGGGGGACGGAAGACTTTTCTTTGCAGGGGAGGCCACTACAAGGCGATACCCTGCCACCATG
CATGGAGCTTTTCTTACTGGACTTCGGGAAGCTGCAAATATGGCTCAATATGCAAAGTCTCGGACTGCAAAGAAA
AAGATCAACAGGAGTCCATCAAGTAATGCTCATTCTTATGCTTCTGCCCTTATGGATTTATTCAGAGAGCCTGATC
TGGAATTTGGGAGTTTTTCTGTTATTTTTTGTCAAAAGAATGCTAATCCGAAGTCACCAGCCATTCTAAGGGTGAA
AATTAGTGAGCCCCGAAAGAGGAATCTGGAAAGCTCAAAGACAGATCAGCAACATTCGAATAAGGTGCTTTTTC
AGCAGCTCCAATCACATTTTAATCAGCAACAACAGTTGCATGTTTATACATTGTTATCAAAGAAACAGGCATTTGA
GCTGAGAGAAGTGAGAGGTGGTGATGAGATGAGGTTGAACTATCTGTGCGAAAAGCTGGGAATTAAGCTGGTG
GGACGTAAGGGTTTGGGACCTACTGCCGATTCCATCATTGCTTCTATTAAAGCACAGAAGGGCGTTCGAAAACCT
TCTTCAACTCCTTTGGCTCCAAAATCAGGGACATCAATGCTGAAAATTGGCACTTTAAAGCAAAAGTTCATCAGG
TAA

GhPAO5

ATGGAAAGTGATGACGGTTCAAACTCCAAGGCAGGTCCACAATTGCAATTTGATTTGGGGAATAATGTTGAGCTA
GGGCTAGAAAAGGTTACATTACAGAATTCTTTGAAACTTAGGAACAAGAAAATGGATGGTGGTGGGGGCTCTAA
GAAAAGACTAAAAGTAACAGCGGTGGAGGTTGATGTTGATTCAGATGATGATGAGCCCATTTTGTCCTTGTTGAA
GTTAAGAAAACCCAAGAATCCTAAAAAGGATAAGGCTGGTTTGGAAGGCAGCGCTGGGAAGTGCAAGAAGGTT
GAAGTTAAAGCAGTTAAAACTGAGGGCAAGAATGAGGAGGATTTGGGGGGAATGAATGATACGTTGGCCAGCT
TTAGAAAGAAGCTAAAGGATCCCAAGAAAGATGTTGATCCAGGAGCAAAGAGGGAAAGGGATTATTCTTTGAAT
AAGTCTGTGGAGGGTGGTGTAGTTTTGGATGGGAAATCTGTGTCGAACACTGGTGTGAAAGGTCAGGATATTGGT
GAAGACAGGTCTGATGCGGTTACTGATACAGTTTTCGAAAGAAAGCATACAGGGAAAGTAAGGAGAGCCAAGT
TTGATTCAAAATCCAAGCTCATCGAGGTTGATGATGAATCCAGAGCTAAGCTAGAGGAAGATCAGAATGAGGGA
GGTTTGTCGCCTGAGGGTGGTTTGAATCAACATTCTCACGAGGCACAATCTGATTCAGTGAGGAAATCTTGCCCA
ATTACGAGTTTGAAACATAATTGCAAGGGTTCCCATCATGCTTCTGCTTCAAAGAATCCTAGCAGAAATTATGGTG
ATAGGTCTCATTCAGATTCTAGTTCAAGCTTCTCACATTCATTCTCCAAAGAATGCAACACAGCTGAGAATCAAG
GATTTGGCCATAGTGTGTGTCAACAAGAAAGCATTTTGGAACCAGGTGACTTAAATGTTCAAAAGGGTCCTTCAG

GGGATCCATGTAGGTCACCTAAAGTTTGTGACAAAGAAAAATATGGGCATTCCAACATTGAGCTCAGGGACAAT
TGCTCAGCAGTTGACCAGAGGAATAAGCCAGAAAGTGGAGGTTCACGACAAACTAAACATAACCTGTTACCGTC
TGTTGTTGATTCACTGAAGATGGAAGATACTTGCACTGATGTTCCAAATGCTTGCGCTGAGGAAAACTCTTTAGA
AGACTCTGTTCATCCCAATGAATTTGTTGCCTCCATTCAGAGGTGCAACTCTGCTCTCCGTCAACCTTCTGAAGAT
GCATGCCATGGTGCGTGTGGTCCCAGCCATGATACTCTTTTCATCAGCAAAGAGGCCAATGTTGATTCTCCCACAT
CAACACCTGATGAAAATGAAAGTTTTCATGAAGATGCAATCTCTCTCCCCAGTTCTGAAATCAAAGACAGTATGT
CATCAGCTGTACAGCGAGGTGGGCGCAGTATTAAAAAGCGTAGACATGGAGATATGGCTTATGAAGGGGATGCT
GATTGGGAGAATTTGCTAAATGAGCAAGGGTTTTTGGAAATCAACAGTTTGCAGACAGTGATCGTTCCTTTAGA
GCAAAAGAGAAGTTTGATGAGGCAGCAGTATCATCTGGACTGAAAGCTCGTGCTGTGGGACCAGTTGAGAAGAT
CAAATTTAAGGAGGTCTTGAAGGGTAGAGGTGGGCTACAGGAATACTTGGAATGCAGGAATCATATCTTAGGTCT
TTGGAGTAAAGATGTTAACCGCATTTTGCCTCTTGCTGAGTGTGGTGTTACTGACACTCCTTCAGAGGGTGAACC
ACCCCGAGCTTCCCTAATCAGGGAGATATATGCATTTCTTGATCAGGGTGGTTACATAAACTTTGGAATTGCTTCA
AAGAAAGAAAAAGCTGAGCTTAGGGTTAAGGATAACCGCAAGCTTCTAAAGGAAAGAAAAAATTATGGCAATT
CTGTGGCCTCTGTTGCTGATTCAGAGGATGGAGTTGCCTTCATCCTTGGCCAAGTCAAGAATTCTGAAGCCTCAAT
GGATGCAAAGATCAGTGTTAGAGTTGATGATGAAAACCAGGCATCTGAAGCCACAATACCTGAAGTGTTGGTTG
ATTCGATCACATCAGAATTACCTTGCAGAAAAGAACAAAAGGAACACCCAAGTGATAATTGCCAGCAAAATGG
CAGCATCAGTGCAAAACTAAACCCTTTATTGATTAGTTCGCAGGTTCCAAGTGCAGATCTATCTTGTGATGCTATT
GACATGGGAATAGCCCCTGTAGTAACTCCAGAAGAAAGAAATGACTCTCATTATGTTCAGTCTGCAACTTATGAT
AAACCTGATGGGAATCATCAACTGCAGGGTGATTCAGAGGTCAGAAAGAACATCATAATTGTTGGAGCTGGTCC
TGCTGGATTGACTGCTGCACGCCACTTGAAACGTCAGGGATTTTCTGTAGTAGTACTTGAGGCTAGGGATAGGAT
AGGAGGTCGTGTTTATACTGATTGCTCCTCTCTTTCAGTACCTGTGGATCTTGGGGCTAGCATTATTACTGGAGTTG
AGGCTGATGTGTCAACTAATAGAAGACCAGATCCATCCTCACTGATTTGTGCACAGTTGGGGCTAGAGTTGACTG
TGTTGAATAGTTCCTGTCCTCTTTATGACATTGTATCTGGTCAAAAGGTTCCTGCTGATCTGGATGATGCTCTGGAA
GCTGAATACAATAGTCTTCTTGATGATATGGTGTTTCTTGTTGCTCAAAAAGGTCAAAAAGCAATGACAATTTCTC
TTGAGGATGGTTTAGAATTTGCCCTAAAAAGGCATCGGATGGAAGAAATAGGAGCTGATATTGAAGAAATAGAA
TCACATTCTTCAGTGGATGCTGTCTATGACTTGAAAGCAAGCAATGGAAAAAAATGTTCTGAAGGGGAGATTTTG
AGTCCTCTTGAGAGAAGGGTTATGAATTGGCACTATGCCCACTTGGAGTATGGCTGTGCTGCTCCGCTTAAGGAA
GTGTCTCTTCCCAATTGGAATCAAGATGATGTTTATGGCGGCTTTGGAGGAGCCCATTGTATGATTAAAGGAGGTT
ACAGTAAGGTGGTTGAGTCTCTTGGAGAAGGACTTCTGATCCACTTGAGCCATGTAGTCACAAATATTTCATACG
GCCCAAAGGACCCTGGGATTGATAATAGTCATCATAGGCAGGTCAAAGTTTCAACGTCAAATGGCAGTGAATTTT
CAGGAGATGCTGTGCTGATCACTGTGCCACTTGGTTGCTTGAAAGCAGGAGCCATAAAGTTTTCTCCTCCATTGC
CCCAATGGAAACATTCTTCCATACAGCAACTTGGTTTTGGAGTACTTAATAAAGTTGTTTTGGAATTTCCAGAAGT
TTTTTGGGATGATACTGTGGATTACTTTGGAGTGACTGCTGAGGAAACAGATAGTAGAGGCCATTGCTTTATGTTT
TGGAATGTCCGAAAAACTGTTGGGGCTCCTGTTCTTATAGCCTTAGTGGCTGGTAAGGCAGCTATTGATGGTCAA
ACTATGAGCTCATCAGATCATGTAAACCATGCTGTACTTATTCTCCGAAAACTTTTTGGTGAGGCTTCAGTTCCTGA
TCCTGTTGCCTCAGTTGTAACTGATTGGGGAAGGGATCCTTTCAGTTACGGTGCTTACTCCTATGTTGCCATTGGA
GCATCTGGAGAAGACTATGATATGCTGGGCAGGCCTGTTGAGAACTGCTTGTTTTTTGCTGGAGAAGCTACCTGC
AAGGAGCATCCTGACACAGTTGGTGGTGCAATGTTGAGTGGACTTCGGGAGGCTGTGCGATTAATTGACATATTT
ACCACTGGAAATGATTATACAGCTGAAGTAGAGGCAATGGAGGGTGCACAGAGACGATCAGAATCAGGAAGGG
ATGAAGTTAGGGACATAATTAAGAGACTTGAAGCAGTTGAACTTTCTAATGTCTTGTACAAAAACTCTTTGGATC
GTGCTTGGGTTTTGAGCAGGGAAGCTTTACTACAGGACATGTTCTTTAATGTGAAAACCACTTCAGGACGACTGC
ATCTAGCCAAAAAATTGTTGGGTCTCCCAGTTGAATCCTTGAAATCCTTTGCTGGGACAAAGGAAGGGCTTAGCA
CACTCAACTCATGGATGCTGGATTCGATGGGGAAAGATGGGACTCAGTTGTTGCGCCATTGTGTTCGTCTTCTTGT
GCTTGTTTCAACTGATCTACTTGCAGTTCGTTCATCAGGCATAGGGAAAACTGTGAAGGAAAAAATTTGTGTGCA

	TACAAGTCGTGATATACGTGCTATAGCAGCCAGCTGGTTAGTGTTTGGCTTGAAGTCTTCCGTAAGGCAAAAGCTT<br>CTTCAAAGAGAAAACCCCTTAAAGATACTGCTTCAGGAAAGCCGCCTCTACACTCACAACATTGTGCTTTTGAG<br>AGTAAAGCAAGCTTGCAGGATCCATTTCCTGCTGGAAAGCAGTATCCTTTCTATGCAAAAGAGAATGGCAAATCG<br>GTTGATATGGAGGTGGAATCTGTCAACCAAGGAATGTCAGAGGAAGAGCAGGCTGCCTTTGCTGCTGAAGCAGC<br>TGCCCGAGCTGCAGCAAAAGCAGCTGCAGAGGCACTTGCATCCACAGAAGCCAACTGCAACAAATTGCTGCAG<br>CTTCCTAAAATTCCTTCTTTTCACAAATTTGCCAGAAGGGAGCAATATGCACAAATGGATGAAGGGAAATGGCCT<br>GGTGGTGTTTTAGGAAGACAAGATTGTATATCAGAAATAGATTCTAGGAACTGCAGAGTCAGAGACTGGTCTGTT<br>GATTTCTCTGCTGCTTGTGTTAACCTTGACAGTTCCAGAATGTCAGTAGATAACCTGTCTCAGAGGAGCCACTTGA<br>AGCTTAGAGAACACTCTGGAGAAAGTTTGGCTGTGGACAGTAGTATCTTCACAAAAGCATGGGTTGAGAATGCT<br>GGTAGTGAGGGGATTAAGGATTGTCATGCCATTGAGAGATGGCAGTCTCAAGCAGCTGCTGCTGATCCAGATTTC<br>TTCCATCCTACAAATTTCAAGGATGAGGAAGATTCAAATGCTAGTTCAAGGCAAACAACCTGGAAGCATGATGG<br>ACGAGCAAATGAGAGCTCCATCTCCCAAGTTTCTGTTAACAAGGAGCGATTTGAAAATCATCCCCATGGTACTGA<br>TCGTATTAAGCAGGCTGTCGTTGATTATGTTGCATCATTGCTAATGCCCCTTTATAAGGCAAGAAAAATTGATAAG<br>GAGGGATACAAATCGATAATGAAGAAAACTGCGACAAAGGTAATGGAGCAGGCAACAGATGCAGAGAAAAAC<br>ATGGCTGTTTCTGAATTTCTAGATTTCAAGCGCAAAAATAAGATTCGCCCCTTTGTAGACAAATTGATTGAGAGGC<br>ACATGGCAATGAAGCCAATCATGAAACCATGA
GhPAO6	ATGGCTAAGCCGAGGATTGTGATAATTGGAGCAGGAATGGCTGGTCTTGCAGCAGCCAACAAGCTTTACACTAG<br>TTCAAATGACTTGTTTGAACTATTTGTTGTTGAAGGTGGTACTAGAATTGGGGGAAGGATCAATACATCAGAGTTT<br>TATGGTGATAGAGTTGAAATGGGTGCTACTTGGATTCATGGTATAAAAGGTAGCCCAGTTCATCAAATTGCTCAAC<br>AAATAAATGCATTACAAGGGTCTGATAAGCCATGGGAGTGTATGGATGGGTTACTTGATGAACCAAAGACCATTG<br>CTGAAGGTGGGTTCGAGCTAAATGGCTCTATGATTGAACCCATATCGACACTTTTTAAGAACTTGATGGATTTTGC<br>TCAAGGCAATGAAGCATCCAAATGGTGTTCGATGAGTTGTCGCTTACGTAACAAAAGTGTTGGTTCTTTTTTAAG<br>AAAAGGCCTTGATGTGTATTGGGATTCTAGTAAAGACCGTGAAGAGCTTAAAGGGTATGGTAAATGGAGTAGAG<br>AGTTGCTTGAAGAAGCCATTTTTGCAATGTATGAGAACACACAAAGGACTTATACATCAGCTGGTGATCTTTTCA<br>GTTTAGATTATGAAGCAGAAAGTGAGTACCGTATGTTCCCTGGTGAAGAAATCACTATTGGTAACGGATATTCCA<br>GTATAATCGAATACCTCGCGTCGGTACTCCCACGGGACGTAATCCAATTAGACCGAAAAGTCGCTAAAATCGAAT<br>GGGATCGTTGTGATTCGAGGCCTGTGAAGATACACTTCTTGGATGGATCTTTTGTGGTAGCTGATCATGTTATTGTC<br>ACAGTTTCGTTAGGGGTTTTAAAAGCTGGTATTTGTAACGCTTCAGGTTTGTTTAGTCCTCCACTTCCTTCTTTCAA<br>AACAGATGCTATATCAAGACTTGGATATGGTGTTGTTAACAAGCTGTTTCTCCGATTAAACGGTAATCGAAAACCC<br>GAAGAGCTCCCTTCCTTGCAAATGGTGTTTCATCGTTCGGATTCCGAGTTAAGGCATAAAAAGATCCCATGGTGG<br>ATGAGAAGGACAGCTACTTTATCCCCTATTTACAACAATGCAAGTGTGTTCCTATCTTGGTTTGCAGGGAAAGAA<br>GCTCTTGAACTCGAAAGACTTAGCAACGAAGAGATTATAAACGCGGTAACAACGACAGTTTCGAGTTTATTATCG<br>AAACCCCATAATGAAATCATGTCTGATAGCAACTCCAATGGATTTGAAGTGAGTTTTGTTGATGTATTGAAGAGC<br>AAATGGGGGAGTGATCCATTGTTCTTAGGGTCATACAGTTATGTTGCTGTTGGATCGAGTGGTGCTGATTTTGACA<br>CCATGGCTGAACCATTACCTACTGATGTGTATCATCATCGTCCCCTTCAAATTTTGTTTGCTGGGGAAGCTACACAT<br>AGAACTCACTATTCCACAACTCATGGAGCGTACTTTAGTGGTATTAGGGAAGCCAATAGGCTTCTTCAACATTATC<br>ATTGTGTTGGGGTTTAA
GhPAO7	ATGGATCTGCTCCGCTTCCAAGGCATTGAGAGGCAGGAGGAGGCAGCAGTTCCCTGTGTCATTGTGATAGGAGG<br>TGGTATTTCCGGCCTTGCTGCCGCTCGGACTCTGACTGATGCTTCTTTCAAGGCAATGATTCTACACCATTATTGGC<br>TACACGGAGTATGCAATGAGAATCCCTTAGCTCCATTAATATCCTCTCTGGGGCTTAAATTGTACCGTACTAGTGGT<br>GACAATTCTGTGTTGTATGACCATGATTTGGAAAGTTATGCACTTTTTGATATGGATGGTCGTAAAGTTCCACAAG<br>AGATTGTTGTTGAAGTTGGAGATGTATTCAAGAGAATACTCAAAGAGACTGAGAAAGTACGGGATGAACACAAG<br>AAGGACATGTCAGTCCTTAAAGCAATATCAATTGTGCTAGATAGGAATCCTGAGTTAAGACAAGAAGGACTTGC<br>CTATGAAGTGATGCAGTGGTACATATGTAGAATGGAAGCTTGGTTTGCTGCAGATACAGATATGATATCCTTGAAA

TGCTGGGATCAGGCAAATCAACTCCACTTCAACTTTAAACTCTACCTACATCTTTCACTATTGCAAATCAGAATCT
CGAAGCTTTTCAACACTGGTTTGCATGAAGTGACGCTGATTAATTTAGAACAAGTCCTTTTGGGTGGTCATGGACT
TATGGTGCAGGGTTATGACCCCATAATAAAAGAACTTGCTAAAGATATTGATGTTCGCTTGAATCATAGTAGGGTT
TCTAAAATATCCAGAGGATGTGATAAGGTGGTGGTCAAAGTTGAGAACGGATTGAGCTTCATTGCTGATGCTGCT
ATAGTAACTGTACCCCTCGGGGTTCTTAAAGCCAATTTGATTCAGTTTGAACCAAAGTTGCCAGAATGGAAGGTT
GCTGCAATTTCAGATATTGGTGTTGGTAACGAAAACAAGATTGCCTTACTATTTGACCGAGTCTTTTGGCCAAATG
TTGAGCTGTTAGGCATTGTTGCACGCACTTCTTATTCTTGTGGTTATTTTCTCAATCTTCACAAGGCAACAGGCCAT
CCTATTCTTGTCTATATGGCTGCTGGAAGATTTGCTGACGATCTGGAGAAGTTTTCTGATGAATATGCTGTGAACTT
TGTGATGTCGCAGTTGAAGAAAATGTTTCCTGATGCAACTGAGCCGGTACAATATCTGGTGTCACATTGGGGAAC
AGATCCAAATTCCCTTGGCTGTTATTCGTATGATCCAGTCGGGATGGCAGGAGATGTGTATGATAAGCTTAGAGAA
CCTTTGGATAATCTTTTCTTTGGAGGGGAAGCAGTTACCGAGGAGCACCAAGGGTCGGTGCACGGAGCTTACTCT
TCTGGAGTCCTGGCTGCCAGAAACTGTGAGAGTCATCTCTTAGAGAGATTAGGTGACTTTAGAAAGCTCCAGCTA
ATCTCCTTTAGTGGTGATGCATTATTAGAACCCATATTTCCTCTCCAGATATCTAGGATGTGA

GhPAO8

ATGGATTCTTCTTCACGCTCCGCTGTCATCATCATCGGCGCCGGCATCTCTGGTATATCGGCGGCGAAGGTTTTGG
CTGACAACGGAATTGCGGATTTGCTGATTTTGGAAGCTTCCGGTAGAATTGGAGGTAGGATCCGGAAAGAAAGT
TTCGGTGGGGTGTCGGTGGAGCTTGGAGCGGGTTGGATCGCGGGTGTAGGTGGCAAAGCGTCCAATCCCGTTTG
GGAGATAGCTTCTAAGTTTGGCCTCCGAACCTGCTTCTCTGACTACAGTAATGCCCGCTATAACATCTACGATCGG
AGTGGGAAGATCTTTCCGAGTGCAATCGCCGCAGACTCATACAAGAAAGCGGTGGACTCGGCGATACAGAAACT
AAGGGACCTAGAGTCAAACTCTGTCGAGGATGTCGCCAATGGAGCCGAGTTACCTTTAACAGCGAAGACACCG
ATAGAGCTCGCGATTGACTTTATATTACACGATTTCGAGATGGCAGGCAAGTTCCACAACTTATTTTTGTTTTCCTC
TGTGGAGCCAATATCAACATACGTAGATTTTGGGGAAAGAGAATTTTTGGTGGCAGATGAAAGGGGTTATGAGTA
TTTACTGTATAAAATGGCAGAGGATTTTCTATTGACGTCGGAGGGAAAAATCCTGGATAATCGCCTCAAACTCAAT
AAGGTTGTCAGGGAATTACAGCACTCGAGAAACGGCGTCACGGTGAAAACAGAGGATGGTTGTGTTTACGAAG
CCAACTACGTCATTTTGTCAGCTAGCATCGGTGTTCTCCAAAGCGACCTCATTTGCTTCAAGCCGCCCTTGCCCAG
GTGGAAAACGGATGCCATAGAGAAATGTGATGTGATGGTATATACCAAGATCTTCCTCAAGTTCCCGTACAAGTT
TTGGCCTTGTGGGACAGACAAAGAGTTCTTCATCTATGCTCACGAGCGGAGAGGCTATTACACGTTTTGGCAGCA
TATGGAAAATGCGTACCCTGGTTCGAATATTTTGGTGGTAACATTGACCAATGGTGAATCAAAACGTGTTGAAGC
TCAATCTGATGAAGAGACGTTAAAGGAAGCAATGGGTGTGCTGAGGGACATGTTTGGGCCCGACATACCTACTG
CTACAGATATACTTGTTCCCCGATGGTGGAATAATAGGTTCCAGCGTGGCAGCTACAGCAATTACCCCATAATCTC
TAATAACCAAGTTGTTAATGATATTAAGGCCCCAGTTGGACGCATTTTTTTTACTGGTGAACACACAAGTGAAAGA
TTTAATGGTTATGTGCATGGAGGATACCTTGCAGGTATTGATACAAGTAAAGCTTTACTGGAAGAAATAAGAAAA
GACGAAAGAGAAAATGAGAGTAAAAGTTTCTTGCTGGAGCCATTAATAGCATTGTCAGGGTCATTAACTTTGGCA
CAGTCGGATGCAGTCTCAGGTCTCCAAAAATGTGAGGTTCCAACGCAATTATATCTTAGCGGCAAGCTTGGCATT
CCAGAAGCAATCTTATGA

GhPAO9

ATGGAGCCTTCTCCTCGGTCGTCCGTTATTATCATCGGCGCCGGCGTCTCTGGTTTATCGGCGGCGAAGGTTTTGG
CTGAGAATGGAATTGGGGATTTGTTGATCTTAGAAGCGTCTGATAGAATTGGCGGTAGGATCCGGAAAGAGAAG
TTCGGAGACGTCTCGGTGGAGCTGGGAGCGGGTTGGATTGCCGGTGTAGGTGGCAAAGAGTCCAACCCGGTTTG
GGAGATTGCCGCGAAGCTTGGCCTCCGAACCTGTTTCTCTGACTACAGCAATGCCCGCTATAACATCTACGATCG
GAGCGGGAAGATATTTCCAAGTGGAATCGCCGCCGACTCGTACAAGAAGGCGGTGGACTCAGCGATTCAGAAA
CTAAAGGGCCTCGAGTCAAACTATGTGGAAGATGCCACCAATAGAACCGACTTTACTTTAACACCGAAGACACC
AATAGAGCTCGCAATTGACTTTATATTACACGATTTTGAGATGGCAGAGGTGGAGCCAATATCAACTTACGTAGAT
TTTGGGGAAAGAGAGTTTTTGGTGGCAGATGAAAGAGGTTATGAATATTTACTGTATAAAATGGCCGAGGAATTT
CTATTTACCTTGGAGGGTAAAATCCTGGACAATCGCCTGAAACTGAACAAGGTTGTTAGGGAATTACAGCACTCG
AGAAACGGCGTCACAGTGAGAACAGAGGATGGTTGCGTTTACGAAGCCGACTACGTGATTTTGTCTGCTAGCAT

	TGGTGTTCTTCAAAGCGACCTCATTTCCTTCAGGCCACCCTTGCCTAGGTGGAAAACGGAAGCGATAGAGAAAT<br>GTGATGTGATGGTGTATACCAAGATCTTCCTCAAGTTTCCGTATAAGTTCTGGCCTTGTGGGCCTGGAAAAGAGTT<br>CTTTATCTATGCTCACGAGAGGAGAGGCTATTACACGTTTTGGCAGCACATGGAGAATGCATACCCTGGTTCAAAT<br>ATTTTGGTTGTAACGTTAACGAACGATGAATCGAAGCGTGTGGAATCTCAATCCGATGAAGAGACATTGAAGGA<br>AGCTATGGTTGTGCTTAGGGACATGTTCGGGTCTGACATACCCGATGCCACTGATATACTTGTTCCCCGCTGGTGG<br>AATAACAGGTTCCAGCGTTGCAGCTACAGCAACTACCCTATGATATCTAATAACCAAGTCATTAATGATATTAAGG<br>CCCCAGTGGGACGCATTTTCTTTACTGGTGAACACACGAGTGAAAGATTTAATGGCTATGTACATGGTGGACACC<br>TTGCAGGCATTGATACTAGTAAGGCAGTACTGGAAGAAATGAGAAAAGATGAAAGACAGAACGACAAACAGA<br>ACCAAAATTTCTTGTTAGAGCCCTTGTTAGCATTGACTCTGACTCAGGCGGATGCAGTCTCAGGTCTCCACAAAT<br>GTGATGTTCCCACACAATTGTATCTCAGCGGCAAGCTTGGCATTCCGGAAGCGATCTTGTGA
GhPAO10	ATGGAGTCGTCGGACAAGAGTAATCACCAATTGCGTGGAGGTATTTGCTATCCAAATGGGGCGAGGAGGCCTGT<br>AAGAACACCTTCAGTAATCGTGATTGGGGCTGGAATGGCTGGAATTGCAGCTGCACGTGCTCTCCATGAAGCCTC<br>ATTTCAGGTTACGGTGTTAGAATCCAGGGACAGAATTGGAGGTCGAGTTCATACTGATTACTCATTTGGTTTTCCT<br>GTTGACCTTGGTGCTTCATGGTTGCATGGAGTTTCGAAAGAAAATCCCTTGGCACCATTGATCAGTAGACTTGGA<br>CTACCACTTTATCGGACTAGTGGTGATAACTCTGTGCTGTATGACCATGACTTGGAGAGTTATGCACTTTTTGATAT<br>GGATGGTCATCAAGTTCCACAGGAATTGGTCACTAAGGTTGGAGAAACATTTGAGTGCATTTTGGAAGAGGCAA<br>TGAGACAAGAGCACAGTGAAGACATGTCCATAAGTAGTGCTTTCTCAATTGTTTTCGAAAGAAGACCAGCATTA<br>AGGTTGCAAGGGCTTGCACATAAGGTACTTCAGTGGTATGTATGCAGAATGGAAGGTTGGTTTGCTTCGGATGCT<br>GATACCATCTCCCTTAAAAGCTGGGACCAGGCAGAGCTATTACCTGGTGGTCACGGACTCATGGTCAGGGGCTAT<br>CTTCCTGTCATAAACACTCTGGCTAAAGGTATTGACATCCGCTTGAGCCACAGGGTTACAAACATTGTGAGGCGT<br>TACACTGGAGTGAAGGTTACTGTGGAAGACGGTACTACATTTGCAGCAGATGCTGTTATCGTTGCTGTTCCTCTAG<br>GTGTACTAAAAGCCAAGACAATCAAGTTCGAACCAAGGCTTCCTGAATGGAAGGAAGCAGCAATTGATGAACTT<br>GGAGTGGGAATTGAGAATAAAATTATATTGCACTTTGACAAGGTGTTTTGGCCTAATGTGGAGTTTTTGGGAGTTG<br>TTGCTGACACATCTTATCATTGCAGCTACTTTCTAAACCTTCATAAGGCGACAGGTCATTCTGTCCTCGTTTATATG<br>CCTGCTGGGCAGCTGGCCAGAGACATTGAGAAAATGTCTGATGAAGCTGCTGTGGAGTTTGCTTTTATGCAACTC<br>AAGAAGATCCTTCCAGAGGCATGTGCCCCGATTCAGTATCTTGTTTCTCGATGGGGCACAGATGTGAACACACTA<br>GGCTCCTATAGCTATGATGCAGTAGGCATGTCCCATGACCTGTATGAGAGGCTAAGGGTGCCAGTGGATAACATAT<br>TCTTTGCGGGGGAGGCAACCAGTATGAGCTACCCAGGGTCCATTCATGGTGCATTTTCAACTGGGCAGATGGCTG<br>CTGAGGACTGTAGGATGCGTGTACTGGAGCGATATGGAGAGTTGAACTTGCTCCAACCAGTTATGGGCGAGGAA<br>GCAGGGTTGTGCGTCCCGCTTTTAATAACGCGCTTGTAA
GhPAO11	ATGGAGACGCCAGTTTCAGAAGGGTTGGTTTCAAAGAGGTCGTTGAGGAAGAAATCCGCAGTGAAGAATTATGA<br>TGAGAATTTAATGGATGAGTTCATAGAGAAGCATATAGGTGGTTCGTTTAGGAAGATTAGAACAAAGGAGGAGTT<br>GGAGAAAGAGACTGAAACTGAGGCAATGATAGCATTATCTTTGGGTTTCCCTATTGATGCACTGATTGAGGATGA<br>AATTAAAGCAGGAGTGGTAAGAGATATAGGTGGAAAAGAGCAGAATGATTACATTGTTCTCAGGAATCATATCCT<br>CTCTCGATGGAGGAGTAATGTACGGATATGGTTATCTAAAGGACATATAAGAGAAACCGTGAGTAATGAATATGA<br>ACATCTGTTGTCTGCTGCTTATGATTTTCTTCTGTATAATGGGTATATTAATTTTGGAGTTTCACCATCCTTTTCCTCT<br>TACATCCCAGCGGAGGCAACTGAGGGTTCTGTGATAATAGTTGGAGCTGGACTTGCTGGCTTGGCAGCAGCAAG<br>GCAACTTATATCTTTTGGTTTCAAGGTTGTTGTTATAGAAGGGAGGAATCGACCTGGGGGAAGAGTTTATACTCAA<br>CTGATGGGTAAGAAGGATAAGCGTGGTGCTGTGGATCTTGGTGGTAGTGTAATCACCGGCATCCATGCCAATCCT<br>CTTGGAGTTCTGGCCCGGCAACTTTCTATTCCACTTCATAAGGTCCGAGATAATTGCCCTTTATATAAACCTGATGG<br>GGTGCCTGTCAATAAGGTAATCGATTCCAAGACTGAAATGATCTTTAACAAGTTGCTTGACAAAGTCAATGAACT<br>GAGAAAAATAATGGGTGGATTTGCTAATTATATTTCTCTTGGATCAGTTCTGGAAAAGCTAAGACAGTTGTATGGT<br>GTGGCTAGAAGCCCAGAGGAGAGACAACTTTTGGAATGGCATCTTGCTAATTTGGAATATGCAAATGCAGGATGT<br>CTTTCTGACTTGTCGGCTGCCTACTGGGACCAGGATGATCCTTATGAGATGGGTGGAGACCATTGTTTTCTTGCTG

GAGGGAACTGGAGATTGATAAAAGCATTATGTGATGGAGTTCCCATAATCTATGGGAAAACAGTTGATGCTATTA
GATATGGTGTTGAAGGAGTTGAGGTTGTTACCGGTAAGCAAGCATTCCAAGCAGATATGGTTCTTTGTACTGTGCC
TCTTGGAGTCTTGAAGAGAAGGACCATTAGATTTGAGCCAGAGTTACCTCAAAGAAAGCTAGCTGCAATTGACA
GACTAGGTTTTGGGCTCCTGAATAAAGTTGCCATGATTTTCTCTCATGTTTTTTGGGGAGAAGAGTTGGACACATT
TGGATGTCTCAATGATACCAGTGATAACCGTGGGGAGTTCTTTTTATTCTACAGCTACCACACTGTTTCCGGGGGT
CCAGTGTTGATTGCGCTGGTGGCTGGTAAAGCTGCACAAACATTTGAGCGCACAGATCCTTCACTCTTGCTCCAT
CGTGTTCTAAGCAAACTTAGAGGTATATATGGTCCAAAAGGTGTAGATGTACCCGACCCTATACAGACAATTTGTA
CAAGATGGGGAAATGATCCCTTTTCATATGGTTCATACTCTCATGTTAGGGTACAGTCATCCGGCAGGGATTATGA
TATACTTGCAGAAAGTATAGGCAATAGGTTGTTTTTTGCTGGTGAAGCCACAACTCGGCAATATCCAGCCACCAT
GCATGGTGCCTATTTGAGTGGGTTAAGGGAAGCTTCGCGTATTCTCCGTGCCACAAGAGGTCGCCAAAACTACTT
TAGGAGGTCTGTGCAGCGGAATGTTGGACCAAGCAGTGATCAATTGGGTGATCTGTTCAAGATGCCTGATCTAGT
ATTTGGGAAGTTCTCTTTTGTGTTCAATCCATTAACAGAGGACCCTAAATCATTGGGGCTTTTGAGAATTACTTTTG
ATAATTGCACAGAAGATACGAGGAAGGTGCTGGAAAAAAGCTGTGACCCCCAATCGAATCAATCATTGCAGCTG
TACACAGCATTGTCCCGTGAACAGGCACATGAGCTACAGATGGTAACTGGAGAAGATGAAAGTAAGTTGGTTTA
TTTGATAAATAATATTGGATTAAAGCTTATGGGAGCTAATGCTCTTGGAATCACATATAACTCCTTGGTTACTAGCA
TATCTAGTGCAAGAAAAGGTAGGAGCAGGTACTGTATATCTGCGCCACTGCTAAATACAGTTTAG

GhPAO12

ATGGATGGTGGTGGAGGCTCTAAGAAAAGATTAAAGGTCACAGCGGTGGAGGTTGGTGTTGATTCAGATGATGA
TGAGCCCATTTTGTCTTTGTTGAAGTTAAGGAGACCTAAGAATCCTAAAAAGGATAAGGCTGGATTGGAAGGCA
GTGCGGGGAAGTGCCAAAAGGTTGAAGTTAAGGCAGGTAAAACTGTGGACAAAGATGAGGAGGATTTTGGGGG
AATGAATGATACATTGGCCAGCTTCAGAAAGAAGTTAAAGGATCCTAAAAAAGATATTGATCCAGGGGCAATGA
GGGTAAGGAGTAACTCTTTGAATAAGTCTGTGGAGGGTGGTGGAATTTTGGATGGGAAATCTGTGTCGAACACTG
ATGTGAAAGGTCAGGATATTGGTGAAGACAGGTCTGATGTGGCTACTGATAAAGTTGTTGGAAAAAAGCGTACA
GGGAAAGTAAGGAGAACCAAGTCAGATTCAAAAGCCAAGCCCAGTGAGGTTGATGATGAATCCAGAGCTAAAC
TTGAGGAAGATCAGAATGAGGGAGGTTTGTCGCCTGGGGAAGGTTTGTATCAGTGTTCTCACAAGGCACAATCT
GGTTCAGTAGGGAAATCTTGCCCAATTTTTTGTTTGAAACATAATTGCGAGGCTGCTCATCATGCTTCTGATTCAA
AGAATCCTAGCAGAAATTGTGGTGATAGTTCTCATTCAGTTTCTAGTTCAAGCTTCTCACATTCATCCTCCAAAGA
ATGCAACACAGCTGAGAATCAAGGATTTGACCACAGTTTGTATCAACAAGAAAGCATTTTGGAACCAGGTGACT
TAACTGTTCAAAAGGATCCTACAGAATATCCATGTAGGTCATCTAAAGTTTGTGACAAGGAAAGATATTGTCATTC
CAACATTGAGCTCAGGGACAATTTCTCAGCAATTGACCTGAGGAGTAAACCAGGAACTGAAAGTTCACAACAA
AATAAACTTAATCTGTCGCTGTCTGTTGTTGATTCACTGAAGATGGAAGAAACTTGCACTGACGTTCCAAATTCTT
GCGCTGAGGAATACTCTTTAGAAACTTCCATTCATCCCAATGAACTTGTTGCCTCCATTCAGAGGTGCAACTCTGC
TCTGCATCAACCTTCTGAAGATGCAAGCCCTGGTGCTTGTGGTCCCATCCATGATACTGTTTTCATCAGCAAAAAG
GCCAATGTTGACTCTCCCATTTCAACACCTGATGAAAATGAAAGTTGCCATGAAGATGCAGTCTCTCTCCCTAGTT
CTGAACTAAAAAACAGCAAGTCATCAGCTTTCCAGCGAGGTGGGCGCAATATTAAAAAGCGTAGACATGGAGAT
ATGGCTTATGAAGGGGATGCTGATTGGGAAAATTTGCTAACTGAGCAAGGTTTTTTTGGAAATCAACAATTTGCA
GACAGTGATCATTCCTTTAGAGCAAGAGAGAAGTTTGATGAGGCAGCAATATCATCTGGACTGAAAGCTTGTGCT
GTGGGGCCAGTTGAGAAGATCAAATTTAAGGAGGTGTTGAAGTGTAGAGGTGGTCTACAGGAATACTTGGAATG
CAGGAATCATATCTTAGGTCTTTGGAGTAAAGATGTTAACCGCATTTTGCCTCTTGTTGACTGCGGTGTTAGTGAC
ACTCCTTCAGAGGGTGAACCATCCCGAGCTTCCCTAATTAGGGAGATATATGCATTTCTTGATCAGGGTGGTTACA
TAAACTTTGGAATTTCTTCAAAGAAAGAGAAGGCTGAGCTTAGTGTTAAGGACAACTACAAACTTCTCGAGGGA
AGAAAAAGTGATGGTAATTCTGTTGCCTCTGTTGCTGATTCAGAGGATGGAGTTGCCTTTATCCTTGGTCAGGTCA
AGAATTCTAAAGCTTCTATGGATGCAAAGACTGGTCTTAGAGTTGTTGATGAAAACCAGGCATCTGAAGCGACA
ATAGCAATAGCTGAAGTATTGGTTGATTCAATCACACCAGAATTACCTTATAAATGCCAGCAAAATGGCAGCTTTA
GTGCAAAATTGAACACTGGATTGATTAGTTCGCAGGTTTCTAGTACTGATCTATCTTGTGATGCAACTGATGTTGG

AGTAGCCCCTGTAGTAACTCCAGAAGAAAGGAATGACTCACAGTATGTTCAATCTGCAACTTATGATAAACCTGA
CGGGAATCATCAGCTGCTGAATGATTCAGAGGTCAGAAAGAACATCATAGTTATTGGAGCTGGTCCTGCTGGATT
GACTGCTGCACGCCACTTGAAACGTCAGGGATTTTCTGTAGTTGTACTTGAGGCTAGGGATAGGATAGGAGGTCG
TGTTTATACTGATTGCTCCTCTCTTTCAGTACCTGTGGATCTTGGGGCTAGCATTATTACTGGAGTTGAGGCTGATG
TGTCAACTAATAGAAGACCAGATCCATCCTCATTGATTTGTGCACAGTTGGGGCTAGAGTTAACTGTGTTGAATAG
TTCCTGTCCTCTTTATGACATTGTATCTGGTCAAAAGGTTCCTGCTGACCTGGATGATGCTCTTGAAGCTGAATACA
ATAGTCTTCTTGACGATATGGTGTTTCTTGTTGCTCAAAAAGGTAAAAAAGCAATGACAATGTCTCTTGAGGATGG
TTTAGAATATGCCCTAAAAAGGCATCGGATGGAAGAAATAGGAGCAGATATTGAAGAAACAGAATCACATTCTT
CAGTGGATGCTTTCTATGACTCAAAAGCTAGCAATATCTTTGGATTTCCTCGAAAAAAATGTTCCNATGATGCTCT
GGAAGCTGAATACAATAAGGAGATTTTGAGTCCTCTTGAGAGAAGGGTCATGAATTGGCACTATGCTCACTTGGA
GTATGGCTGTGCTGCTTCGCTTAAGGAAGTTTCTCTTCCCAACTGGAATCAGGATGATGTTTATGGCGGCTTTGGA
GGAGCCCATTGTATGATTAAAGGGGGTTACAGTACGGTGGTTGAGTCTCTTGGAGAAGGGCTTCTGATCCACTTG
AACCACGTAGTCACAAATATTTCATACAGCCCAAAGGGCCCGGGGGTTGATAATAGTCATCATAGGCAGGTCAA
AGTTTCCACATCAAATGGCAGTGAGTTTTCAGGAGATGCTGTGCTTATCACTGTGCCACTTGGTTGCTTGAAAGC
AGGAGCCATAAAGTTTTCTCCTCCGTTGCCCCAATGGAAACATTCTTCCATACAGCAGCTTGGTTTTGGAGTACTT
AATAAAGTCGTCTTGGAATTCCCAGAAGTTTTTTGGGATGATACTGTGGATTACTTTGGAGTGACTGCTGAGGAA
ACAGATAGTAGAGGCCATTGTTTTATGTTTTGGAATGTCCGAAAAACTGTTGGGGCTCCTGTTCTTATAGCCTTAGT
GGCTGGTAAGGCAGCTATTGATGGTCAAAATATGAGCGCATCAGATCATGTAAACCATGCTGTAATTATTCTCCGT
AAACTTTTTGGAGAGGCTTCAGTTCCTGATCCTGTTGCCTCAGTTGTTACTGATTGGGGAAGGGATCCTTTTAGTTA
TGGCGCTTACTCCTATGTTGCCATAGGAGCATCTGGAGAAGACTATGATATGTTGGCCAGGCCTGTTGAGAACTG
CTTGTTTTTTGCTGGAGAAGCTACCTGCAAGGAGCATCCTGACACAGTTGGTGGTGCAATGTTGAGTGGGCTTCG
GGAGGCTGTGCGATTAATTGACATATTTACCACTGGAAATGATTATACAGCCGAAGTAGAGGCAATGGAGGCTGC
CCAGAGACATTCAGAATCAGGAAGGGATGAAGTTAGGGACATAATTAAGAGACTTGAAGCAGTTGAACTTTCTA
ATGTCTTGTACAAAAACTCTTTGGATCGTGCTCGGATTTTAAGCAGGGAAGCTTTACTGCGGGACATGTTCTTTAA
TGTGAAAACCACTGCAGGACGATTGCATCTAGCCAAGAAGTTGTTAGGTCTCCCAGTTGAATCCTTGAAATCCTT
TGCTGGGACAAAGGAAGGGCTTAGCACACTCAACTCATGGATGCTGGACTCAATGGGGAAAGATGGGACGCAA
CTGTTGCGCCATTGTGTTCGTCTTCTTGTGCTTGTTTCAACTGATCTACTTGCAGTTCGTTCATCAGGCATAGGGAA
AACTGTGAAGGAAAAAATTTGTGTGCATACAAGTCGTGATATACGTGCTATTGCAAGCCAGCTGGTTAATGTTTG
GCTTGAAGTCTTCCGTAAGGCAAAAGCTTCTTCAAAGAGAAAATCCCTTAAAGATCCAGCTTCAGGAAAGCCAC
CTCTGCACTCACAGCATGGTGCTTTTGAGAGTAAAGAAAGCTTGCAGGATCCATTTTCTGCTGGAAAGCAGTATC
CTTTAAACATAAAGGAGAATGGCAAATCACTTGATATTGAGGTGGAAGCTGTCAACCAAGGAATGTCAGAGGAA
GAGCAGGCTGCCTTTGCTGCTGAAGCAGCTGCCCGAGCTGCAGCAAAAGCAGCTGCAGAGGCACTTGCATCCA
CAGAAGCTAACTGCAACAAATTACTGCAGCTTCCTAAGATTCCTTCTTTTCACAAATTTGCCAGAAGGGAGCAAT
ACGCACAAATGGATGAAGGGAAATGGCCTGGTAGTGTTTTTGGAAGACAAGATTGTATATCAGAAATTGACTCTA
GGAACTGCAGAGTCAGGGACTGGTCTGTTGATTTCTCTGCTGCTTGTGTTAACCTTGACAATTCCGGAATGTCAGT
AGATAACCTGTCTCAGAGGAGCCACTTGAAACTCAGAGAACACTCTGGAGAAAGTTTGGCTGTGGATAGCAGTA
TCTTCATGAAAGCATGGGTTGATACTGCTGGTAATGGGGGGATCAAGGATTATCATGCCATTGAGAGATGGCAGT
CTCAAGCAGCTGCTGCTGATCCAGATTTCTTCCATCCTACAAATTTCAAGGATGAGGAAGATTCAAATACTAGTTC
AAGGCAACCAACCTGGAAGAATGATGGGCGAGCAAATGAGAGCTCCGTCTCCCAAGTTTCTGTAAACAAGGAG
CGATTTGAAAATCATCCCCATGGAGCTGATTGTATTAAACAGGCTGTCGTTGATTATGTTGCATCATTGCTAATGCC
CCTTTATAAGGCAAGAAAAATTGATAAGGAGGGATACAAATCGATAATGAAAAAAACTGCAACAAAGGTAATG
GAGCAGGCAACAGATGCAGAGAAAAACATGGCTGTTTTTGAATTTCTTGATTTCAAGCGCAAAAACAAGATTCG
TCCCTTTGTAGACAAATTGATTGAGAGGCACATGGCAATGAAGCCAACCATGAATCTATGA

GhPAO13 ATGGAAAGTGATGACGGTTCAAACTCCAAGGCAGGTCCACAATTGCAATTTGATTTGGGGAATAATGTTGAGCTA

GGGCTAGAAAAGGTTACATTACAGAATTCTTTGAAACTTAGGAACAAGAAAATGGATGGTGGTGGGGGCTCTAA
GAAAAGACTAAAAGTAACAGCGGTGGAGGTTGATGTTGATTCAGATGATGATGAGCCCATTTTGTCCTTGTTGAA
GTTAAGAAAATCTAAGAATCCTAAAAAGGATAAGGCTGGATTGGAAGGCAGCGCTGGGAAGTGCAAGAAGGTT
GAAGTTAAAGCAGTTAAAACTGAGGGCAAGAATGAGGAGGATTTGGGGGGAATGAATGATACGTTGGCCAGCT
TTAGAAAGAAGCTAAAGGATCCCAAGAAAGATGTTGATCTAGGAGCAAAGAGGGAAAGGGATTATTCTTTGAAT
AAGTCTGTGGAGGGTGGTGGAGTTTTGGATGGGAAATCTGTGTTGAACACTGGTGTGAAAGGTCAGGATATTGGT
GAAGACAGGTCTGATGTGGTTACTGATACAGTTGTCGAAAGAAAGCATACAGGGAAAGTAAGGAGAGCCAAGT
TTGATTCAAAATCCAAGCTCATCGAGGTTGATGATGAATCCAGAGCTAAGCTAGAGGAAGATCAGAATGAGGGA
GGTTTGTCGCCTGAGAGTGGTTTGAATCAACATTCTCACGAGGCACAATCTGATTCAGTGAGGAAATCTTGCCCA
ATTTCGAGTTTGAAACGTAATTGCAAGGGTTCCTATCATGCTTCTGCTTCAAAGAATCCTAGCAGAAATTATGGTG
ATAGGTCTCATTCAGATTCTAGTTCAAGCTTCTCACATTCATCCTCCAAAGAATGCAACACAGCTGAGAATCAAG
GATTTGACCATAGTGTGTGTCAACAAGAAAGCATTTTGGAACCAGTTGACTTAAATGTTGAAAAGGGTCCTACAG
AGGACCCATGTAGGTCACCTAAAGTTTGTGACAAAGAAAAATATGGGCATTCCAACATTGAGCTCAGGGACAAT
TGCTCAGCAGTTGACCAGAGGAATAAGCCAGAAAGTGAAGGTTCACCACAAACTAAACATAATCTGTTACCGTC
TGTTGTTGATTCACTGAAGATGGAAGAGACTTGCACTGATGTTCCAAATGCTTGCGCTGAGGAAAACTCTTTAGA
AAACTCTGTTCATCCCAATGAATTTGTTGCCTCCATTCAGAGGTGCAACTCTGCTCTCCGTCAACCTTCTGAAGAT
GCATGCCATGGTGCTTGTGGTCCCACCCATGATACCCTTTTCATCAGCAAAGAGGCCAATGTTGATTCTCCCACAT
CAACACCTGATGAAAATGAAAGTTTTCATGAAGATGCAGTCTCTCTCCCCAGTTCTGAAATCAAAGACAGTATGT
CATCAGCTGTCCAGCGAGGTGGGCGCAGTATTAAAAAGCGTAGACATGGAGATATGGCTTATGAAGGGGATGCT
GATTGGGAGAATTTGCTAAATGAGCAAGGGTTTTTGGAAATCAACAGTTTGCAGACAGTGATCGTTCCTTTAGA
GCAAAAGAGAAGTTTGATGAGGCAGCAGTATCATCTGGACTGAAAGCTCGTGCTGTGGGACCAGTTGAGAAGAT
CAAATTTAAGGAGGTCTTGAAGGGTAGAGGTGGGCTACAGGAATACTTGGAATGCAGGAATCATATCTTAGGTCT
TTGGAGTAAAGATGTTAACCGCATTTTGCCTCTTGCTGAGTGCGGTGTTAGTGACACTCCTTCAGAGGGTGAACC
ACCCCGAGCTTCCCTAATCAGGGAGATATATGCATTTCTTGATCAGGGTGGTTACATAAACTTTGGAATTGCTTCA
AAGAAAGAAAAAGCTGAGCTTAGGGTTAAGGATAACCACAAGCTTCTCAAGGAAAGAAAAAATTATGGCAATT
CAGTGGCCTCTGTTGCTGATTCAGAGGATGGAGTTGCCTTCATCCTTGGCCAGGTCAAGAATTCTGAAGCCTCTTT
GGATGCAAAGGTCGGTGTTAGAGTTGATGATGAAAACCAGGCATCTGAAGCCACAATACCTGAAGTGTTGGTTG
ATTCGATCACATCAGAATTACCTTGCGGAAAAGAACAAAAGGAACACCCAAGTGATAATTGCCAGCAAAATGG
CAGCATCAGTGCAAAACTAAACCCTTTATTGATTAGTTCGCAGGTTCCAAGTGCAGATCTATCTTGTGATGCTATT
GACATGGGAATAGCCCCTGTAATAACTCCAGAAGAAAGAAATGACTCACATTATGTTCAGTCTGCAACTTATGAT
AAACCTGATGGGAATCATCAACTGCAGGGTGATGCAGAGGTCAGAAAGAACATCATAATTGTTGGAGCTGGTCC
TGCTGGATTGACTGCTGCACGCCACTTGAAACGTCAGGGATTTTCTGTAGTTGTACTTGAGGCTAGGGATAGGAT
AGGAGGTCGTGTTTATACTGATTGCTTCTCTCTTTCAGTACCCGTGGACCTTGGGGCTAGCATTATTACTGGAGTTG
AGGCCGATGTGTCAACTAATAGAAGACCAGATCCATCCTCATTGATTTGTGCACAGTTGGGGCTAGAGTTGACCG
TGTTGAATAGTTCCTGTCCTCTTTATGACATTGTATCTGGTCAAAAGGTTCCTGCTGATCTGGATGATGCTCTGGAA
GCTGAATACAATAGTCTTCTTGATGATATGGTGTTTCTTGTTGCTCAAAAAGGTCAAAAAGCAATGACAATTTCTC
TTGAGGATGGTTTAGAATATGCCCTAAAAACGCATCGGATGGAAGAAATAGGAGCTGATATTGAAGAAATAGAA
TCACATTCTTCAGTGGAAGCTGTCTATGACTTGAAAGCAAGCAATGGAAAAAAATGTTCTGAAGGGGAGATTTT
GAGTCCTCTTGAGAGAAGGGTTATGAATTGGCACTATGCCCACTTGGAGTATGGCTGTGCTGCTCCGCTTAAGGA
AGTGTCTCTTCCCAATTGGAATCAAGATGATGTTTATGGCGGCTTTGGAGGAGCCCATTGTATGATTAAAGGAGGT
TACAGTAAGGTGGTTGAGTCTCTTGGAGAAGGACTTCTGATCCACTTGAACCATGTAGTCTCAAATATTTCATACG
GCCCAAAGGACCCTGGGATTGATAATAGTCATCATAGGCAGGTCAAAGTTTCCACATCAAATGGCAGTGAGTTTT
CAGGAGATGCTGTGCTGATCACTGTGCCACTTGGTTGCTTGAAAGCAGGAGCCATAAAGTTTTCTCCTCCATTGC
CCCAATGGAAACATTCTTCCATACAGCAACTTGGTTTTGGAGTACTTAATAAAGTTGTTTTGGAATTTCCAGAAGT

TTTTTGGGATGATACTGTGGATTACTTTGGAGTGACTGCTGAGGAAACAGATAGTAGAGGCCATTGCTTTATGTTT
TGGAATGTCCGAAAAACTGTTGGGGCTCCTGTTCTTATAGCCTTAGTGGCTGGTAAGGCAGCTATTGATGGTCAA
ACTATGAGCTCATCAGATCATGTAAACCATGCTGTACTTATTCTCCGAAAACTTTTTGGTGAGGCTTCAGTTCCTGA
TCCTGTTGCCTCAGTTGTGACTGATTGGGGGAGGGATCCTTTCAGTTACGGTGCTTACTCCTATGTTGCCATTGGA
GCATCTGGAGAAGACTATGATATGTTGGGCAGGCCTGTTGAGAACTGCTTGTTTTTCGCTGGAGAAGCTACTTGC
AAGGAGCATCCTGACACAGTTGGTGGTGCAATGTTGAGTGGACTTCGGGAGGCTGTGCGATTAATTGACATATTT
ACCACTGGAAATGATTATACAGCTGAAGTAGAGGCAATGGAGGGTGCACAGAGACGATCAGAATCAGGAAGGG
ATGAAGTTAGGGACATAATTAAGAGACTTGAAGCAGTTGAACTTTCTAATGTCTTGTACAAAAACTCTTTGGATC
GTGCTTGGGTTTTGAGCAGGGAAGCTTTACTACGGGACATGTTCTTTAATGTGAAAACCACTTCAGGACGATTGC
ATCTAGCCAAAAAGTTGTTGGGTCTCCCAGTTGAATCCTTGAAATCCTTTGCTGGGACAAAGGAAGGGCTTAGCA
CACTCAACTCATGGATGCTGGATTCGATGGGGAAAGATGGGACTCAGCTGTTGCGCCATTGTGTTCGTCTTCTTGT
GCTTGTTTCAACTGATCTACTTGCAGTTCGTTCATCAGTGGCTACAATGACTTTTTTATTAATAATTTTTCNCCTGAA
ACTTAGAGAACACTCTGGAGAAAGTTTGGCTGTGGACAGTAGTATCTTCACAAAAGCATGGGTTGATAATGCTGG
TAGTGAGGGGATTAAGGATTGTCATGCCATTGAGAGATGGCAGTCTCAAGCAGCTGCTGCTGATCCAGATTTCTT
CCATCCTACAAATTTCAAGGATGAGGAAGATTCAAATGCTAGTTCAAGGCAAACAACCTGGAAGCATGATGGAC
GAGCAAATGAGAGCTCCATCTCCCAAGTTTCTGTTAACAAGGAGCGATTTGAAAATCATCCCCATGGTACTGATC
GTATTAAGCAGGCTGTCGTTGATTATGTTGCATCATTGCTAATGCCCCTTTATAAGGCAAGAAAAATTGATAAGGA
GGGATACAAATCGATAATGAAGAAAACTGCGACAAAGGTAATGGAGCAGGCAACCGACGCAGAGAAAAACAT
GGCTGTTTCTGAATTTCTTGATTTCAAGCGCAAAAATAAGATTCGCCCCTTTGTAGACAAATTGATTGAGAGGCAC
ATGGCAATGAAGCCAATCATGAAACCATGA
ATGGCAAAGAAGCCAAGAATTGTGATAATTGGAGCTGGGATGGCCGGTCTTACTGCAGCTAACAAGCTCTATACT
TCCACTGGCTCTGACCATTTGTTTGAGCTTGTTGTTGTTGAAGGTGGTGATAGAATTGGTGGCAGAATCAACACTT
CGGAGTTTTGTGGTGACAGAATTGAGATGGGTGCTACTTGGATCCATGGTATAGGAGGCAGCCCGGTACATCAAA
TTGCTCGGGAAATCCATGCACTTGAGTCTGATAAGCCATGGGAGTGTATGGATGGGTTCTCGGGTGAGCCAAAGA
CTATTGCTGAAGGTGGGTTCGAGCTAAATGCCTCCATCGTTGACCCCATATCCACCCTTTTCAAAAACCTGATGGA
TTTCGCTCAAGGGAAGCTGACTGAATACAGTGCAGGCAGCGGAGGAGATGCTTGTTACTACAATTTTGCAGCTAA
AGCAGCCTTGAAAGATTGTACGAGCAATGGTGGCTTTGGTAACCAGAGTGTCGGTGCGTTTCTTAGACGAGGCCT
TGGTGCTTACTGGGATTCTTGCAAGGACCGTGAGGAGCTGAACGGATATGGTAAATGGAGCAGAAAATTGCTTG
AAGAAGCCGTTTTTGCCATGCATGAAAACACCCAGAGAACTTATACTTCTGCCGGTGATCTGTTCAATCTAGATTA
CGAGGCAGAAAGCGAGTACCGTATGTTTCCTGGTGAAGAAATCACCATTTCTAAAGGCTATTTGAGCATAATTGA
ACATCTTGCATCTGTTCTTCCTCCTGGCGTAATCCAATTAGGCCGCAAAGTCACAAGAATCGAATGGCAACCTGA
GhPAO14 GGGTCATAAATCTATACAAGTTCCAAACGGCTATGATTCCAGACCAGTGAAGATTGAGTTTTGTGATGGATCTTTT
ATGTTAGCAGATCATGTGATAGTCACAGTTTCATTAGGGGTCTTAAAATCTGGAACTGGTCAAGATTCAGGCATGT
TCAATCCTCCCCTTCCTCCTTTCAAGACAGAGGCTATATCAAGACTTGGATATGGTGTTGTTAACAAGCTGTTCCTT
CAATGGAGTCCAAATGGTAATCGACCGGCCAATGATAAAGAGAAGTTTCCTTCCTTGCAAATTGTTTTCCATCCC
CCAGAATCCGAGTTAAGGCATGAAAAGATCCCAGGGTGGATGAGGAGGACAGCTTCACTGTCTCCTATTTATAAC
AATTCAAGCGTCCTCCTATCCTGGTTTGCAGGTAAAGAAGCACTTGAGCTTGAAACACTTAGCGATGAAGAGATT
ATAAATGGAGTTTCAGCAACAGTATCTGGTTTATTACCAGTATCAAAACACAAGAAGGAAGACCAGTATAATTCC
CCTGAATTCTGCAATGGGAATGTGGAGAGCTGTGATGACAATGGAGTGAGATTTGGTAAGGTTTTGAAGAGCAA
ATGGGGCAGTGATCCATTATTCTTGGGATCTTACAGCTACGTGGCTGTTGGATCAAGCGGTGCTGATTTAGACACA
ATGGCTGAACCCTTACCAAAGCTTGGGAGCACTGACTCAGACCACCATCCACTTCAAATATTGTTTGCTGGGGAG
GCTACACACAGAACCCACTATTCTACAACCCATGGAGCTTATTTCAGTGGTCTTAGGGAAGCCAATAGGCTTCTC
AAACATTATCGTTGTGTTGGGGTTTAG
GhPAO15 ATGGAGCCTCCCCAAGATACCTCCGAGAACCCTAACGATGTCCTTTCCGACGATGACTCTTCACCGGAAAACAC

CAATCCCGACGATCAAGAAATCCCCAGTACGACACTCGACCCACCTATTTCCGATACCCAAGATGAATCCTCCGA
TCCCGTCCCCGACGAGCAACCCCAAAACACTAATTCGAACCCCGCGGAGCCTGGTCCACCTGCACGCAAGCGC
CGCCGCAGAAAGCGTTTCTTTACTGAACTTATCGCCAATCCATCCTTCTCCAAGAACCGTCGCCCTAGAATATCGG
GCCTAGCTAGAGAAATGGACACCGAAGCTTTAATCGCGATTTCTGTTGGTTTCCCTGTTGATTCTCTTACCGAAGA
AGAAATCGAAGCCAACGTGGTGTCCAGAATCGGAGGCCAAGAGCAAGCCAACTACATCGTTGTAAGAAATCAC
ATTCTGGCTCGCTGGAGATCCAATGTATCCGTCTGGCTGACGCGCGAGCACGCCCTCGAATCAATCCGAGCTGAG
CACAAGAACCTAGTGAACGCAGCATACAATTTCCTTCTCGAACACGGTTACATTAATTTCGGTTTAGCCCCGGCT
GTTAAAGAAGCGAAATTGAAGTCTTTTGATGGTGTAGAAAGAGCCAATGTGGTGATTGTGGGTGCGGGTCTTTCC
GGTTTGGTCGCGGCGAGGCAATTAGTTTCCATGGGGTTTAAAGTTGTCATCTTAGAAGGTAGGACGCGCCCTGGA
GGGCGCGTGAAGACAAGGAAGATGAAAGGTGATGGGGTGGTGGCTGCAGCGGATCTTGGTGGGAGTGTTCTTA
CGGGAATAAATGGAAATCCACTTGGGGTTCTTGCAAGGCAAATGGGATTACCGCTTCATAAGGTGCGAGATATTT
GTCCTTTGTATTTGCCAGATGGAAAGGCCGTAGATGCTGATGTTGATTCTAGGATAGAAGTTTCATTTAATAAGCTA
TTGGATAGGGTTTGTAAGCTTAGGCATTCTATGATTGAGGAAGTTAAATCAGTTGATGTTCCATTAGGGACAGCAT
TAGAAGCCTTTAGGAGTGTTTACAAGGTTGCTGAGGATTCACAGGAGAGCATGTTGTTGAATTGGCATCTTGCTA
ATCTTGAATATGCTAATGCTTCCTTGATGGCTAATTTGTCTATGGCCTATTGGGATCAAGATGATCCATATGAGATG
GGCGGCGATCATTGTTTCATACCTGGTGGCAATGAGAGGTTTGTTCGAGCACTTGCGGAGGACCTTCCCATTTTCT
ATGGGAGGACTGTGCAGAGTATCAGGTATGGTATTGATGGTGTTAGGGTTTACGCCGGTGGGCAGGAGTTTTGTG
GGGATATGGCTCTTTGCACTGTTCCATTAGGAGTTCTCAAGAAGGGATCGATAGAATTTGTTCCTGAGCTTCCGCA
AAGAAAGAAGGATGCCATTCAGAGACTGGGATTTGGGTTGCTGAATAAGGTTGCTATGTTGTTTTCATACAATTTT
TGGGGCGGAGAGATTGATACTTTTGGCCACCTGACAGAAGACCCAAGTATGAGAGGCGAGTTCTTTTTGTTTTAT
AGCTATTCTTCTGTGTCAGGTGGTCCACTCCTTGTTGCTCTAGTTGCCGGAGATGCAGCAATCAAGTTTGAACTGA
TGTCTCCTGTTGAGTCTGTGAAAAGGGTTTTAAACATATTGCGAGGCATTTTTCATCCAAAAGGGATTGTTGTTCC
TGATCCTGTCCAGGCTGTTTGTACCCGGTGGGGAAAGGATCGCTTCACGTATGGATCCTACTCTCATGTTGCTATT
GGTTCATCCGGGGATGATTATGATATTCTAGCTGAGAGTGTTGGAGATGGGAGAGTCTTCTTTGCTGGTGAGGCAA
CTAATAAGCAGTATCCTGCCACAATGCATGGAGCCTTTTTAAGTGGCATGAGAGAGGCCGCTAACATGCTTAGAG
TGGCCAGGAGGAGGTCATTGGTTCTATCTGACAAAGTTAATAACGACTTGGAGAAATGTGATACTTTGGATAAGT
TGTTTGAGAACCCTGACCTGACATTCGGGAGCTTCTCAGCTTTGTTTGATCCCCATTCTAATGATGTTGGATCGCAT
GCATTAATAAGGGTCAAATTTCATGGGGATAAATTAACCTCGAGTCACTTGTGTCTTTATGGCTTGATAACGAAGA
AGCAAGCCATTCAGTTAAGTGAAATGAATGGAGATGGGAACAGGATGAATTCGTTGTATCGTGACTTTGGGGTG
AAGTTGGTTGGTGGTAAAGGGTTATCAAATGTTGCGGAGTTGCTGATATCACGCATCAAAGCAGCTAAACCAACC
TAA

ATGAACTCACCAAATGAAACCCCTGATCAATTCTCCCAATTCCCCCTTCCCCATTTCACTCTCACTCCACCTTTAC
CAAACCCTAACCCTAATTTCCCTCCAACCCCAAACTCAACTCCAACACTAACCCCAGTCCTCGATTCTAACACCA
ATGCTACACCTTCTCTCGATGATCAACTTCTACCCTTCCCAGTTCCCAAAAAACGACGACGCGGCAGGCCCCGAC
GCACTGCCTCAACGTCATCGTTTCAACTCCTTACCTTCCCCAACGATTCATTCAACCCCAATGTTCCATACTCTGA
CCCTAACCCTTATTCGATTCCCTCATCAGTAGCGGCGTCGACACAAACTTCACAACCCAAAATTGCTGACGAGAT
CATTGTTATCAATAAAGAATCGACGGCTGAGGCTCTCACCGCTCTTTCCGCTGGATTCCCTGCTGATTCTCTCACT
GhPAO16 GAGGAAGAAATTGACTTCGGCGTAGTTTCCTCTGTTGGTGGCATCGAGCAGGTAAATTACATTCTTATTCGAAATC
ACATTATTGCGAAATGGCGTGAAAATATATTCAATTGGGTGACTAAAGAAATGTTTGTTGATTCTATACCACAACA
TTGTCGTACGCTCTTAGATTCTGCTTATGATTATTTGGTTACTCATGGATATATAAATTTTGGGGTTGCCCCAGCAAT
CAAGGACAAAATTCCTGTCGGTCTTAGTAAAGGTAATGTGGTTATCATTGGTGCTGGATTGGCGGGGCTGGCTGC
GGCTAGACAGCTAATGAGGTTCGGATTTAAGGTGACGGTTTTGGAAGGGAGGAAGAGAGCAGGTGGGAGGGTT
TATACAAAGAAGATGGAAGGAGGGAATAGGGTGAGTGCAGCTGCGGATTTAGGTGGGAGTGTATTAACAGGTAC
ATTGGGGAATCCATTAGGGATCATGGCAAAACAATTGGGTGCTTCGCTTTTTAAGGTGAGGGATAAGTGTCCACT

TTATCGGATGGATGGGAGTCCGGTGGATCCAGATATGGATATGAAGGTGGAGACGGCTTTTAATCGGCTTTTGGAT
AAGGCTAGTAAGCTTAGGCAGTTAATGGGGGAGGTTTCCATGGATGTTTCACTTGGGGCAGCATTAGAGACGTTT
AGACAGGTTTATAGAGATGCAGTAACTGAAGAGGAGATTAATCTGTTCAATTGGCATCTTGCAAATTTAGAATATG
CAAATGCAGGATTGGTTTCAAAGCTTTCACTTGCATTTTGGGACCAAGATGATCCATATGACATGGGAGGGGATC
ATTGTTTCTTGCCTGGAGGGAATGGAAGGTTGATTCAGGCTCTAGCAGAGAATGTGCCTATTTTATATGAGAAGAC
TGTGCATACTATTAGGTATGGAAGTGATGGAGTGCAGGTTACGGCAGGAAATCAGGTGTTTGAAGGTGATATGGC
ACTATGTACTGTTCCTCTTGGAGTTTTAAAGAGTGGGTCAATAAAGTTTGTTCCTGAGTTGCCTCAGAGGAAACTT
GATGGGATAAAGAGGTTGGGATTTGGGTTGTTGAATAAGGTCGCTATGCTTTTCCCTTATGTATTTTGGGGTACAGA
TCTTGATACCTTTGGGCATCTTACTGAAGATCCAAGTTGCCGAGGGGAGTTTTTTCTATTTTATAGCTATGCAACAG
TTGCTGGTGGTCCTCTCTTGCTTGCTTTAGTAGCAGGAGAAGCTGCACATAGGTTTGAGACTCTGCCTCCTACAGA
TGCAGTAACCCAAGTTCTCCAAATTCTCAAGGGTATATATGAACCGCAAGGAATCACTGTCCCTGAACCCCTCCA
AACCGTCTGTACTAGATGGGGTGGTGATCCCTTTAGCCTAGGTTCATACTCTAATGTTGCTGTGGGAGCATCTGGA
GATGACTATGATATATTAGCAGAAAGTGTTGGGGATGGAAGACTTTTCTTTGCTGGGGAGGCCACTACACGTCGA
TACCCTGCCACCATGCATGGAGCTTTTCTTACTGGGCTCAGGGAAGCTGCAAATATGGCTCAATATGCCAACGCT
CGGACTGCAAAGAAAAAGATAGACAGGAGTCCTTCAAATAATGTTCATTCTTGTGCTTCCCTCCTTATGGATTTGT
TCAGAGAACCTGATTTGGAATTCGGGAACTTTTCTGTTATTTTTGGTCGAAAGAATGCTGATCCAAAGTCACCAG
CAGTTTTGAGGATAACATTCAGTGAGCCCCGAAAGAAGAATCAGGAAGGTTCAAAGACAGATCAGCAACATTCT
AATAAGGTGCTTTTTCAGCAGCTACAGTCACATTTTAATCAGCAACAACAGCTACATGTTTACACATTGTTATCTA
AGCAACAGGCACTTGAGCTGAGAGAAGTGAGAGGTGGTGATGAGATGAGGTTGAACTACCTCTGTGAAAATCT
GGGAATTAAGCTGGTGGGACGGAAGGGTTTGGGACCTAATGCTGATTCTGTCATTGCATCTATAAAAGCACAGAG
GGGTGTCCGGAAACCCTCAACAACTCCTGTGGTTCTAAAATCTGGGGCATCGAAGATGAAACCAGGCACTTTAA
AGAAAAAATTCATTAGGAGGGCTAAAATAGTCCGCAACACTAAAGGGTTGATTCCAGCTCTGGTTCCGAATGCA
GCAAATGGCAATATGCCAGAGGAAATGAAAGTGATAAAGCTGGCTCCTCCTGACTCCTCTGCTTCGGGTATGTCT
GAAGGCTTCTAG

ATGGCTAAGAAGCCGAGAGTTGTGATAATTGGAGCAGGAATGGCTGGTCTTACAGCAGCCAACAAGCTTTACAC
TAGTTCAAATGACTTGTTTGAACTATTTGTTGTTGAAGGTGGAACTAGAATTGGGGGAAGGATCAATACGTCAGA
GTTTTATAGTGATAGAGTTGAAATGGGTGCTACTTGGATTCATGGAATAAAAGGTAGCCCAGTTCATCAAATTGCT
CAACAAATCAATGCATTACAAGGGTCTGATAAGCCATGGGAGTGTATGGATGGGTTACTTGATGAACCAAAGAC
CATTGCTGAAGGTGGGTTCGAGCTAAATGGCTCTATGATTGAACCCATATCGACACTTTTTAAGAACTTGATGGAT
TTTGCTCAAGGCAATGAAGCATCCAAATGGTGTTCGATGAGTCGTCGCTTAGGTAACAAAAGTATTGGTTCTTTTT
TAAGAAAAGGCCTTGATGTGTATTGGGATTCTTGTAAAGACCATGAAGAGCTTAAAGGGTATGGTAAATGGAGTA
GAGAGTTGCTTGAAGAAGCCATTTTTGCAATGTATGAGAACACACAAAGGACTTATACATCAGCTGGTGATCTTT
TCAGTTTAGATTATGAAGCAGAAAGTGAGTACCGTATGTTCCCTGGTGAAGAAATCACTATTGGTAACGGATATTC
CAGTATAATCGAATACCTCGCGTCGGTACTCCCACGGGACGTAATCCAATTAGACCGAAAAGTCGCTAAAATCGA

GhPAO17

ATGGGATCGTTGTGATTCGAGGCCTGTGAAGATACACTTCTTGGATGGATCTTTTGTGTTAGCTGATCATGTTATTG
TCACAGTTTCGTTAGGGGTTTTAAAAGCTGGTATTTGTAATGATCCAGGTTTGTTTAGTCCTCCACTTCCTTCTTTCA
AAACGGATGCTATATCAAGACTTGGATATGGTGTTGTTAACAAGCTGTTTCTCCGATTAAACGGTAATCGAAAACC
CGAAGAGCTCCCTTCCTTGCAAATGGTGTTTCATCGTTCGGATTCCGAGTTAAGGCATAAAAAGATCCCATGGTG
GATGAGAAGGACAGCTACTTTATCCCCTATTTACAACAATGCAAGTGTGTTCCTATCTTGGTTTGCAGGGAAAGA
AGCTCTTGAACTCGAAAGACTTAGCAACGAAGAGATTATAAAGGCGGTAACAACGACAGTTTCGAGTTTATTATC
GGAACCCCATAATGAAATCATGTCTGATAGCAACTCCAATGGATTTGAAGTGAGCTTTGTTGATGTATTGAAGAG
CAAATGGGGGAGTGATCCATTGTTCTTAGGGTCTTACAGTTATGTTGCTGTTGGATCGTGTGGTGCTGATTTTGACA
CCATGGCTGAACCATTACCTACTGATGTGTATCATCATCATCCACTTCAAATTTTGTTTGCTGGGGAAGCTACACAT
AGAACTCACTATTCCACAACTCATGGAGCTTACTTTAGTGGTATTAGGGAAGCCAATAGGCTTCTTCAACATTATC

	ATTGTGTTGGGGTTTAA
GhPAO18	ATGGATCTGCTCCGCTTCCAAGGCATTGAGAGGCAGGAGGAGGCAGCAGTTCCCTGTGTCATTGTGATAGGAGG<br>TGGTATTTCCGGCCTTGCTGCCGCTCGGACTCTGACTGATGCTTCTTTCAAGGTAATCCTGTTGGAATCACGAGAA<br>AGACTTGGTGGTCGCATCCATACTGATTTCTCTTTTGGTTGCCCTGTGGATATGGGAGCTTCATGGAAAGCTTTAG<br>ATCTTGATATTTCTCTTACTTTGCTTCTTTATAAGCTACACGGGGTATGCAATGAGAATCCCTTAGCTCCATTAATAT<br>CCTCTCTGGGCCTTAAATTGTACCGTACTAGTGGTGACAATTCTGTGTTGTATGACCATGATTTGGAAAGTTATACA<br>CTTTTTGATATGGATGGCCGTAAAGTTCCACAAGAGATTGTTGTTGAAGTTGGAGATGTATTCAAGAGAATACTCA<br>AAGAGACTGAGAAAGTACGGGACGAACACAAGAAGGACATGTCAGTCCTTAAAGCAATATCAATTGTGCTAGA<br>AAGGAATCCTGAGTTAAGACAAGAGGGACTTGCCTATGAAGTGATGCAGTGGTACATATGTAGAATGGAAGCTT<br>GGTTTGCTGCAGATACAGATATGATATCCTTGAAATGCTGGGATCAGGCAAATCAACTCCACTTCAACTTTAAACT<br>CTACATACATCTTTCACTATTGCAAATCAGAATCTCGAAGCTTTTCAACACTGGTTTGCATGAAGTGACGCTGATT<br>AATTTAGAACAAGTCCTTTTGGGTGGTCATGGACTTATGGTGCAGGGTTATGACCCCATAATAAAAGAACTTGCTA<br>AAGATATTGATGTTCGCTTGAATCATAGTAGGGTTTCTAAAATATCCAGAGGATGTGATAAGGTGGTGGTCAAAGT<br>TGAGAACGGATTGAGCTTCATTGCTGATGCTGCTATAGTAACTGTACCCCTCGGGGTTCTTAAAGCCAATTTGATT<br>CAGTTTGAACCAAAGTTGCCAGAGTGGAAGGTTGCTGCAATTTCAGATATTGGTGTTGGTAACGAAAACAAGAT<br>TGCCTTACTATTCGACCGAGTCTTTTGGCCAAATGTTGAGCTGTTAGGCATTGTTGCACGCACTTCTTATTCTTGTG<br>GTTATTTTCTCAATCTTCACAAGGCAACAGGCCATCCTATTCTTGTCTATATGGCTGCTGGAAGATTTGCTGACGAT<br>CTCGAGAAGTTTTCTGATGAATATGCTGTGAAATTTGTGATGTCGCAGTTGAAGAAAATGTTTCCTGATGCAACTG<br>AGCCGGTACAATATCTGGTGTCACATTGGGGAACAGATCCAAATTCCCTTGGCTGTTATTCGTATGATCCAGTCGG<br>GATGGCAGGAGATGTGTATGATAAGCTTAGAGAACCTTTGGATAATCTTTTCTTTGGAGGGGAAGCAGTTACCGA<br>GGAGCACCAAGGGTCGGTGCACGGAGCTTACTCTTCTGGAGTCCTGGCTGCCAGAAACTGTGAGAACCATCTCT<br>TAGAGAGATTAGGTGACTTTAGAAAGCTCCAGCTGATCTCCTTTAGTGGTGATGCATTATTAGAACCCATATTTCCT<br>CTCCAGATATCTAGGATGTGA
GhPAO19	ATGGATTCTTCTTCAAGCTCCGCTGTCATCATCATCGGCGCCGGCATCTCTGGTATATCGGCGGCGAAGGTTTTGG<br>CTGACAACGGAATTGCGGATTTGCTGATTTTGGAAGCTTCCGGTAGAATTGGAGGTAGGATCCTGAAAGAAAGTT<br>TCGGAGGGGTGTCGGTGGAGCTTGGAGCGGGTTGGATCGCTGGTGTAGGTGGCAAAGCGTCCAATCCCGTTTGG<br>GAGATAGCTTCTAAGTTTGGCCTCCGAACCTGCTTCTCTGACTACAGTAATGCCCGCTATAACATCTACGATCGGA<br>GTGGGAAGATCTTTCCGAGTGCAATCGCCGCAGACTCATACAAGAAAGCGGTGGACTCGGCGATACAGAAACTA<br>AGGGACCTAGAGTCAAACTCTGTCGAGGATGTCGCCAATGGAGCCGAGTTACATTTAACAGCGAAGACACCGAT<br>AGAGCTCGCGATTGACTTTATATTACACGATTTCGAGATGGCAGGCAAGTTGCACAACTCATTTTTGTTTTCCCCT<br>GTGGAGCCAATATCAACATACGTAGATTTTGGGGAAAGAGAATTTTTGGTGGCAGATGAAAGGGGTTATGAGTAT<br>TTACTGTATAAAATGGCAGAGGATTTTCTATTGACGTCGGAGGGAAAAATCCTGGATAATCGCCTCAAACTCAAT<br>AAGGTTGTCAGGGAATTACAGCACTCGAGAAACGGCGTCACGGTGAAAACAGAGGATGGTTGTGTTTACGAAG<br>CCAACTACGTCATTTTGTCAGCTAGCATCGGTGTTCTCCAAAGCGACCTCATTTGCTTCAGGCCGCCCTTGCCCAG<br>GTGGAAAACGGATGCCATAGGGAAATGTGATGTGATGGTATATACCAAGATCTTCCTCAAGTTCCCGTATAAGTTT<br>TGGCCTTGTGGGACTGACAAAGAGTTCTTCATCTATGCTCACGAGCGGAGAGGCTATTACACGTTTTGGCAGCAC<br>ATGGAAAATGCATACCCTGGTTCGAATATTTTGGTGGTAACATTGACCAATGGTGAATCAAAACGTGTTGAAGCT<br>CAATCTGATGAAGAGACGTTAAAGGAAGCAATGGGTGTGCTGAGGGACATGTTTGGGCCCGACATACCGACTGC<br>TACAGATATACTTGTTCCCCGATGGTGGAATAATAGGTTCCAGCGTGGCAGCTACAGCAATTACCCCATAATCTGT<br>AATAACCAAGTTGTTAATGATATTAAGGCCCCAGTTGGACGCATTTTTTTTACTGGTGAACACACAAGTGAAAGA<br>TTTAATGGTTATGTGCATGGTGGATACCTTGCAGGTATTGATACAAGTAAAGCTTTACTGGAAGAAATAAGAAAA<br>GACGAAAGAGAAAATGAGAGTAAAAGTTTCTTGCTGGAGCCATTAATAGCATTGTCAGGGTCATTAACTTTGGCA<br>CAGTCGGATGCAGTCTCAGGTCTCCAAAAAATGTGAGGTTCCAACGCAATTATATCTTAGCGGCAAGCTTGGCATT<br>CCAGAAGCAATCTTATGA

GhPAO20	ATGGAGCCTTCTCCTCCGTCGTCCGTTATCATCATCGGCGCCGGCGTCTCCGGTTTATCGGCGGCGAAGGTTTTGG<br>CTGAGAATGGAATTGGGGATTTGTTGATCTTAGAAGCGTCTGATAGAATTGGCGGTAGGATCCGGAAAGAGAAG<br>TTCGGAGACGTCTCGGTGGAGCTGGGAGCGGGTTGGATTGCCGGTGTAGGTGGCAAAGAGTCCAACCCGGTTTG<br>GGAGATTGCCGCGAAGCTTGGCCTCCGAACCTGTTTCTCTGACTACAGCAATGCCCGCTATAACATCTACGATCG<br>GAGCGGGAAGATATTTCCAAGTGGAATCGCCGCCGACTCGTACAAGAAGGCGGTGGACTCAGCGATTCAGAAA<br>CTAAAGGGCCTCGAGTCAAACTATGTGGAAGATGCCACCAATAGAACCGACTTTACTTTAACACCGAAGACACC<br>AATAGAGCTCGCAATTGACTTTATATTACACGATTTTGAGATGGCAGAGGTGGAGCCAATATCAACTTACGTAGAT<br>TTTGGGGAAAGAGAGTTTTTGGTGGCAGATGAAAGAGGTTATGAATATTTACTGTATAAAATGGCCGAGGAATTT<br>CTGTTTACCTCGGAGGGTAAAATCCTGGACAATCGCCTCAAACTGAACAAGGTTGTTAGGGAATTACAGCACTC<br>GAGAAACGGCGTCACGGTGAGAACAGAGGATGGTTGCGTTTTCGAAGCCGACTACGTGATTTTGTCTGCTAGCA<br>TTGGTGTTCTTCAAAGCGACCTCATTTCCTTCAGGCCACCCTTGCCCAGGTGGAAAACGGAAGCCATAGAGAAA<br>TGTGATGTGATGGTGTATACCAAGATCTTCCTCAAGTTTCCGTATAAGTTCTGGCCCTGTGGGCCTGGAAAAGAGT<br>TCTTTATCTATGCTCACGAGAGGAGAGGCTATTACACGTTTTGGCAGCACATGGAGAATGCGTACCCTGGTTCGA<br>ATATTTTGGTTGTAACGTTGACGAACGATGAATCGAAGCGTGTGGAATCTCAATCCGATGAAGAGACATTGAAGG<br>AAGCTATGGTTGTGCTTAGGGACATGTTCGGGTCTGACATACCCGATGCCACTGATATACTTGTTCCCCGCTGGTG<br>GAATAACAGGTTCCAGCGTTGCAGCTACAGCAACTACCCTATGATATCTAATAACCAAGTCATTAATGATATTAAG<br>GCCCCAGTGGGACGCATTTTCTTTACTGGTGAACACACGAGTGAAAGATTTAATGGCTATGTACATGGTGGACAC<br>CTTGCAGGCATTGACACAAGTAAGGCAGTGCTGGAAGAAATGAGAAAAGATGAAAGACAGAAAGGCAAACAG<br>AACCAAAATTTCTTGTTAGAGCCCTTGTTAGCATTGACTCTGACACAGGCGGATGCAGTCTCAGGTCTCCACAAA<br>TGTGATGTTCCTACACAATTGTATCTCAGCGGCAAGCTCGGCATTCCGGAAGCGATCTTGTGA
GhPAO21	ATGGAGTCGTCGGACAAGAGTAATCACCAATTGCGTGGAGGTATTTGCTATCCAAATGGGGCGAGGAGGCCAGT<br>AAGAACACCTTCAGTAATCGTGATTGGGGCTGGAATGGCTGGAATTGCAGCCGCACGTGCTCTCCATGAAGCCT<br>CATTTCAGGTTACGGTGTTAGAATCCAGGGACAGAATTGGAGGTCGAGTTCATACTGATTACTCATTTGGTTTTCC<br>TGTTGACCTTGGTGCTTCATGGTTGCATGGAGTTTCGAAAGAAAATCCCTTGGCACCATTGATCAGTAGACTTGG<br>ACTACCACTTTATCGGACTAGTGGTGATAACTCTGTGCTGTATGACCATGACTTGGAGAGTTATGCACTTTTTGATA<br>TGGATGGTCATCAAGTTCCACAGGAGTTGGTCACTAAGGTTGGAGAAACATTTGAGTGCATTTTGGAAGAGGCA<br>ATGAGACAAGAGCACAGTGAAGACATGTCCATAAGTAGTGCTTTCTCAATTGTTTTCGAAAGAAGACCAGAATT<br>AAGGTTGGAAGGGCTTGCACATAAGGTACTTCAGTGGTATGTATGCAGAATGGAAGGTTGGTTTGCTTCGGATGC<br>TGATACCATCTCACTTAAAAGCTGGGACCAGGCAGAGCTATTACCTGGTGGTCACGGACTCATGGTCAGGGGCTA<br>TCTTCCTGTCATAAACACTCTGGCCAAAGGTATTGACATCCGCTTGAGCCACAGGGTTACAAACATAGTGAGGCG<br>TTACACTGGAGTGAAGGTTACTGTGGAAGATGGTACTACATTTGCGGCGGATGCTGTTATCGTTGCTGTTCCTCTA<br>GGCGTACTAAAAGCCAAGAATATCAAGTTCGAACCAAGGCTTCCTGAATGGAAGGAAGCAGCAATTGATGAAC<br>TTGGAGTGGGAATTGAGAATAAAATTATATTGCACTTTGACAAGGTGTTTTGGCCTAATGTGGAGTTTTTGGGAGT<br>TGTTGCTGACACATCTTATCATTGCAGCTACTTTCTAAACCTTCATAAGGCGACAGGTCATTCTGTCCTCGTTTATA<br>TGCCTGCTGGGCAGCTGGCCAGAGACATTGAGAAAATGTCTGATGAAGCTGCTGTGGAGTTTGCTTTTATGCAAC<br>TCAAGAAGATCCTTCCAGAGGCATGTGCCCCGATTCAGTATCTTGTTTCTCGATGGGGCACAGATGTGAACACAC<br>TAGGCTCCTATAGCTATGATGCAGTAGGCATGTCCCATGATCTGTATGAGAGGCTAAGAGTGCCAGTGGATAACAT<br>ATTCTTTGCGGGGGAGGCAACCAGTATGAGCTATCCAGGGTCCATTCATGGTGCATTTTCAACTGGGCAGATGGC<br>TGCTGAGGACTGTAGGATGCGTGTACTGGAGCGATATGGAGAGTTGAACTTGCTCCAACCAGTTATGGGTGAGG<br>AAGCAGGGTTGTGTGTCCCGCTTTTAATAACGCGTTTGTAA
GhPAO22	ATGGAGACGCCAGTTTCAGAAGGGTTGGTTTCAAAGAGGTCGTTGAGGAAGAAATCCGCAGTGAAGAATTATGA<br>TGAGAATTTAATGGATGAGTTCATAGAGAAGCATATAGGTGGTTCGTTTAGGAAGATTAGAACAAAGGAGGAGTT<br>GGAGAAAGAGACTGAAACTGAGGCAATGATAGCATTATCTTTGGGTTTCCCTATTGATGCGCTGATTGAGGATGA<br>AATTAAAGCAGGAGTGGTAAGAGATATAGGTGGAAAAGAGCAGAATGATTATATTGTTCTTAGGAATCATATCCT

	TTCTCGGTGGAGGAGTAATGTACGGATATGGTTATCTAAAGGACATATAAGAGAAACCGTGAGTAATGAATATGA<br>ACATCTGTTGTCTGCTGCTTATGATTTTCTTCTGTATAATGGGTATATTAATTTTGGAGTTTCACCATCCTTTTCCTCT<br>TACATCCCAGCGGAGGCAACTGAGGGTTCTGTGATAATAGTTGGAGCTGGACTTGCTGGCTTGGCAGCAGCAAG<br>GCAACTTATATCTTTTGGTTTCAAGGTTGTTGTTATAGAAGGGAGGAATCGACCTGGGGGAAGAGTTTATACTCAA<br>CTGATGGGTAAGAAGGATAAGCGTGGTGCTGTGGATCTTGGTGGTAGTGTAATCACCGGCATCCATGCCAATCCT<br>CTTGGAGTTCTGGCCCGGCAACTTTCTATTCCACTTCATAAGGTCCGAGATAATTGCCCTTTATATAAACCTGATGG<br>GGTGCCTGTCAATAAGGTAATCGACTCGAAGACTGAAATGATCTTTAACAAGTTGCTTGACAAAGTCAATGAACT<br>GAGAAAAATAATGGGTGGATTTGCTAATTATATTTCTCTTGGATCTGTTCTGGAAAAGCTAAGACAGTTGTATGGT<br>GTGGCTAGAAGCCCAGAGGAGAGACAACTTTTGGAATGGCATCTTGCTAATTTGGAATATGCAAATGCAGGATGT<br>CTTTCTGACTTGTCGGCTGCCTACTGGGACCAGGATGATCCTTATGAGATGGGTGGAGACCATTGTTTTCTTGCTG<br>GAGGGAACTGGAGATTGATAAAAGCATTATGTGATGGAGTTCCCATAATCTATGGGAAAACAGTTGATGCTATTA<br>GATATGGCGTTGAAGGAGTTGAGGTTGTTACCGGTAAGCAAGCATTCCAAGCAGATATGGTTCTGTGTACTGTGC<br>CTCTTGGAGTCTTGAAGAGAAGGACCATTAGATTTGAGCCAGAGTTACCTCAAAGAAAGCTAGCTGCAATTGAC<br>AGATTAGGTTTTGGGCTCCTGAATAAAGTTGCCATGATTTTCTCTCATGTTTTTTGGGGAGAAGAGTTGGACACAT<br>TTGGATGTCTCAATGATACCAGTGATAACCGTGGGGAGTTCTTTTTATTCTACAGCTACCACACTGTTTCCGGGGG<br>TCCGGTGTTGATTGCGCTGGTGGCTGGTAAAGCTGCACAAACATTTGAGCGCACAGATCCTTCACTCTTGCTCCA<br>TCGCGTTCTAAGCAAACTTAGAGGTATATATGGTCCAAAAGGTGTAGATGTACCTGACCCTATACAGACAATTTGT<br>ACAAGATGGGGAAATGATCCCTTTTCATATGGTTCATACTCTCATGTTAGGGTACAGTCATCCGGCAGGGATTATG<br>ATATACTTGCAGAAAGTATAGGCAATAGGTTGTTTTTTGCTGGTGAAGCCACAACTCGGCAATATCCAGCCACCAT<br>GCATGGTGCCTATTTGAGTGGGTTAAGGGAAGCTTCGCGTATTCTCCGTGCCACAAGAGGTCGCCAAAACTACTT<br>TAGGAGGTCTGTGCAGCGGAATGTTGGACCAAGCAGTGATCAATTGGGTGATCTGTTCAAGATGCCTGATCTAGT<br>ATTTGGGAAGTTCTCTTTTGTGTTCAATCCATTAACAGAGGACCCTAAATCATTGGGGATTTTGAGAATTACTTTTG<br>ATAATTGCACAGATGATATGAGGAAGGTGCTGGAAAAAAGCTGTGGCCCCCAATCGAATCAATCATTGCAGCTGT<br>ACGCAGCATTGTCCCGTGAACAGGCACATGAGCTACAGATGGTAACTGGAGAAGATGAAAGTAAGTTGGTTTAT<br>TTGATAAATAATATTGGATTAAAGCTTATGGGAGCTAATGCTCTTGGAATCACATATAACTCCTTGGTTACTAGCAT<br>ATCTAGTGCAAGAAAAGGTAGGAGCAGGTACCGTATATCTGCACCACTGCTAAATACAGTTTAG
GhPAO23	ATGAACTCACCAAATGAAACCCCTGATCAATTCTCCCAATTCTCCCAATTCCCCCTTCCCCATTTCACTCTCACTC<br>CACCTTTACCAAACCCTAACCCTAATTTCCCTCCAACCCCAAACTCAACTCCAACACTAACCCCAGTCCTCGATT<br>CTAACACCAATGCTACACCTTCTCTCGATGATCAACTTCTACCCTTCCCAGTTCCCAAAAAACGACGACGCGGCA<br>GGCCCCGACGCACTTCCTCAACCTCATCGTTTCAACTCCTTAACTTCCCCAACGATTCATTCAACCCCAATGTTCC<br>ATACTCTGACCCTAACGCTTATTCGATTCCCTCATCAGTAGCGGCGTCGACACAAACTTCACAACCCAAAATTGC<br>CGACGAGATCATCGTTATCAATAAAGAATCGACGGCTGAGGCTCTCACCGCTCTTTCCGCTGGATTCCCCGCTGA<br>TTCTCTCACTGAGGAAGAAATTGACTTCGGCGTAGTTTCCTCTGTTGGTGGCATCGAGCAGGTAAATTACATTCTT<br>ATTCGAAATCACATTATTGCGAAATGGCGCGAAAATATATTCAATTGGGTGACTAAAGAAATGTTTGTTGATTCTA<br>TACCACAACGTTGTCGTACGCTCTTAGATTCTGCTTATGATTATTTGGTTACTCATGGATATATAAATTTTGGGGTTG<br>CCCCAGCAATCAAGGACAAAATTCCTGTCGGTCTTAGTAAAGGTAATGTGGTTATCATTGGTGCTGGATTGGCGG<br>GGCTGGCTGCGGCTAGACAGCTAATGAGGTTCGGATTTAAGGTGACTGTTTTGGAAGGGAGGAAGAGAGCAGGT<br>GGGAGGGTTTATACAAAGAAGATGGAAGGAGGGAATAGGGTGAGTGCAGCTGCAGATTTAGGCGGGAGTGTATT<br>AACGGGTACATTGGGTAATCCATTAGGGATCATGGCAAAACAGTTGGGTGCTTCGCTTTTTAAGGTGAGGGATAA<br>GTGTCCACTTTATCGGATGGATGGGAGTCCAGTGGATCCAGATATGGATATGAAGGTGGAGACAGCTTTTAATCG<br>GCTTTTGGATAAGGCTAGTAAGCTTAGGCAGTTAATGGGGGAGGTTTCCATGGATGTTTCACTTGGGGCAGCATTA<br>GAGACGTTTAGACAGGTTTATAGAGATGCAGTAACTGAAGAGGAGATTAATTTGTTCAATTGGCATCTTGCAAAT<br>TTAGAATATGCAAATGCAGGATTGGTTTCAAAGCTTTCACTTGCTTTTTGGGACCAAGATGATCCATATGACATGG<br>GAGGGGATCATTGTTTCTTGCCTGGAGGGAATGGAAGGTTGATTCAGGCTCTAGCAGAGAATGTGCCTATTTTATA

TGAGAAGACTGTGCATACTATTAGGTATGGAAGTGATGGAGTGCAGGTTACGGCAGGCAATCAGGTGTTTGAAG
GTGATATGGCACTATGTACTGTTCCTCTTGGAGTTTTAAAGAGTGGGTCAATAAAGTTTGTTCCTGAGTTGCCTCA
GAGGAAACTTGATGGGATAAAGAGGTTGGGATTTGGGTTGTTGAATAAGGTCGCTATGCTTTTCCCTTATGTATTTT
GGGGTACAGATCTTGATACCTTTGGGCATCTTACTGAAGATCCAAGTTGCCGAGGGAAGTTTTTTCTATTTTATAGC
TATGCAACAGTTGCTGGTGGTCCTCTCTTGCTTGCTTTAGTAGCAGGAGAAGCTGCACATAGGTTTGAGACTCTGC
CTCCTACAGATGCAGTAACCCAAGTTCTCCAAATTCTCAAGGGTATATATGAACCGCAAGGAATCACTGTCCCTG
AACCCCTCCAAACAGTCTGTACTAGATGGGGTGGTGATCCCTTTAGCCTAGGTTCGTACTCTAATGTTGCTGTGGG
AGCATCTGGAGATGACTATGATATACTAGCAGAAAGTGTGGGGGATGGAAGACTTTTCTTTGCTGGGGAGGCCAC
TACACGTCGATACCCTGCCACCATGCATGGAGCTTTTCTTACTGGGCTCAGGGAAGCTGCAAATATGGCTCAATAT
GCCAACGCTCGGACTGCAAAGAAAAAGATAGACAGGAGTCCTTCAAATAATGTTCATTCTTGTGCTTCCCTCCTT
ATGGATTTGTTCAGAGAACCTGATTTGGAATTCGGGAACTTTTCTGTTATTTTTGGTCGAAAGAATGCTGATCCAA
AGTCACCAGCAGTTTTGAGGATAACATTCAGTGAGCCCCGAAAGAAGAATCAGGAAGGTTCAAAGACAGATCA
GCAACATTCTAATAAGGTGCTTTTTCAGCAGCTACAGTCGCATTTTAATCAGCAACAACAGCTACATGTTTACACA
TTGTTGTCTAAGCAACAGGCACTTGAGCTGAGAGAAGTGAGAGGTGGTGATGAGATGAGGTTGAACTACCTCTG
TGAAAATCTGGGAATTAAGCTGGTGGGACGGAAGGGTTTGGGACCTAATGCTGATTCTGTCATTGCATCTATAAA
AGCACAGAGGGGTGTCCGGAAACCCTCAACAACTCCTGTGGTTCTAAAATCTGGGGCATCGAAGATGAAACCA
GGCACTTTAAAGCAAAAATTCATTAGGAGGGCTAAAATAGTCCGCAACACTAAAGGGTTGATTCCAGCTCTGGTT
CCGAATGCAGCAAATGGCAATATGCCAGAGGAAATGAAAGTGATAAAGCAGGCTCCTCCTGACTCCTCTGCTTC
GGGTATGTCTGAAGGCTTCTAG
