# Supplementary material for: Evolution and Expression Analysis of PAO Gene Family in Cotton: Focusing on Fiber Development and Stress Response
Source: Plants (Basel). 2026 May 7;15(10):1429. doi: 10.3390/plants15101429 (PMC13210522; doi:10.3390/plants15101429)
Supplement: Supplementary file 1 [file plants-15-01429-s001.zip › Supplementary Materials Table S2.pdf]

Table S2. This table shows the ID correspondence of polyamine oxidase (PAO) family genes between the gap-free telomere-to-telomere (T2T) genome and the previous TM-1-NBI genome of *Gossypium hirsutum*.

Name	NBI Genome ID	T2T Genome ID
GhPAO1	Gh_A03G0717	GhChrA03G1053.1
GhPAO1	Gh_A03G0717	GhChrD02G1046.1
GhPAO1	Gh_A03G0717	GhChrA05G4377.1
GhPAO1	Gh_A03G0717	GhChrD04G0450.1
GhPAO1	Gh_A03G0717	GhChrA05G1999.1
GhPAO1	Gh_A03G0717	GhChrD05G1993.1
GhPAO2	Gh_A05G0221	GhChrA05G0311.1
GhPAO2	Gh_A05G0221	GhChrD05G0325.1
GhPAO2	Gh_A05G0221	GhChrD07G0118.1
GhPAO2	Gh_A05G0221	GhChrA07G0125.1
GhPAO3	Gh_A05G1548	GhChrA05G1926.1
GhPAO3	Gh_A05G1548	GhChrD05G1915.1
GhPAO4	Gh_A05G2520	GhChrA05G3219.1
GhPAO4	Gh_A05G2520	GhChrD05G3102.1
GhPAO4	Gh_A05G2520	GhChrD06G2285.1
GhPAO4	Gh_A05G2520	GhChrA06G2339.1
GhPAO4	Gh_A05G2520	GhChrD05G3101.1
GhPAO5	Gh_A05G3233	GhChrA05G4377.1
GhPAO5	Gh_A05G3233	GhChrD04G0450.1
GhPAO5	Gh_A05G3233	GhChrA03G1053.1
GhPAO5	Gh_A05G3233	GhChrD02G1046.1
GhPAO5	Gh_A05G3233	GhChrA05G1999.1
GhPAO5	Gh_A05G3233	GhChrD05G1993.1
GhPAO6	Gh_A07G0104	GhChrA07G0125.1
GhPAO6	Gh_A07G0104	GhChrD07G0118.1
GhPAO6	Gh_A07G0104	GhChrD05G0325.1
GhPAO6	Gh_A07G0104	GhChrA05G0311.1

GhPAO7	Gh_A08G0331	GhChrA08G0426.1
GhPAO7	Gh_A08G0331	GhChrD08G0446.1
GhPAO7	Gh_A08G0331	GhChrD12G1150.1
GhPAO7	Gh_A08G0331	GhChrA12G1258.1
GhPAO8	Gh_A08G0507	GhChrA08G0650.1
GhPAO8	Gh_A08G0507	GhChrD08G0645.1
GhPAO8	Gh_A08G0507	GhChrD08G1941.1
GhPAO8	Gh_A08G0507	GhChrA08G2037.1
GhPAO9	Gh_A08G1292	GhChrA08G2037.1
GhPAO9	Gh_A08G1292	GhChrD08G1941.1
GhPAO9	Gh_A08G1292	GhChrD08G0645.1
GhPAO9	Gh_A08G1292	GhChrA08G0650.1
GhPAO10	Gh_A12G2582	GhChrD12G1150.1
GhPAO10	Gh_A12G2582	GhChrA12G1258.1
GhPAO10	Gh_A12G2582	GhChrA10G0408.1
GhPAO10	Gh_A12G2582	GhChrA08G0426.1
GhPAO11	Gh_A13G1224	GhChrA13G1936.1
GhPAO11	Gh_A13G1224	GhChrD13G1861.1
GhPAO12	Gh_D02G0971	GhChrD02G1046.1
GhPAO12	Gh_D02G0971	GhChrA03G1053.1
GhPAO12	Gh_D02G0971	GhChrA05G4377.1
GhPAO12	Gh_D02G0971	GhChrD04G0450.1
GhPAO12	Gh_D02G0971	GhChrA05G1999.1
GhPAO12	Gh_D02G0971	GhChrD05G1993.1
GhPAO13	Gh_D04G0374	GhChrD04G0450.1
GhPAO13	Gh_D04G0374	GhChrA05G4377.1
GhPAO13	Gh_D04G0374	GhChrA03G1053.1
GhPAO13	Gh_D04G0374	GhChrD02G1046.1
GhPAO13	Gh_D04G0374	GhChrA05G1999.1

GhPAO13	Gh_D04G0374	GhChrD05G1993.1
GhPAO14	Gh_D05G0300	GhChrD05G0325.1
GhPAO14	Gh_D05G0300	GhChrA05G0311.1
GhPAO14	Gh_D05G0300	GhChrD07G0118.1
GhPAO14	Gh_D05G0300	GhChrA07G0125.1
GhPAO15	Gh_D05G1723	GhChrD05G1915.1
GhPAO15	Gh_D05G1723	GhChrA05G1926.1
GhPAO16	Gh_D06G1841	GhChrD06G2285.1
GhPAO16	Gh_D06G1841	GhChrA06G2339.1
GhPAO16	Gh_D06G1841	GhChrA05G3219.1
GhPAO16	Gh_D06G1841	GhChrD05G3102.1
GhPAO17	Gh_D07G2378	GhChrD07G0118.1
GhPAO17	Gh_D07G2378	GhChrA07G0125.1
GhPAO17	Gh_D07G2378	GhChrD05G0325.1
GhPAO17	Gh_D07G2378	GhChrA05G0311.1
GhPAO18	Gh_D08G0428	GhChrD08G0446.1
GhPAO18	Gh_D08G0428	GhChrA08G0426.1
GhPAO19	Gh_D08G0594	GhChrD08G0645.1
GhPAO19	Gh_D08G0594	GhChrA08G0650.1
GhPAO19	Gh_D08G0594	GhChrD08G1941.1
GhPAO19	Gh_D08G0594	GhChrA08G2037.1
GhPAO20	Gh_D08G1583	GhChrD08G1941.1
GhPAO20	Gh_D08G1583	GhChrA08G2037.1
GhPAO20	Gh_D08G1583	GhChrD08G0645.1
GhPAO20	Gh_D08G1583	GhChrA08G0650.1
GhPAO21	Gh_D12G0881	GhChrD12G1150.1
GhPAO21	Gh_D12G0881	GhChrA12G1258.1
GhPAO21	Gh_D12G0881	GhChrA10G0408.1
GhPAO21	Gh_D12G0881	GhChrA08G0426.1

GhPAO22	Gh_D13G1522	GhChrD13G1861.1
GhPAO22	Gh_D13G1522	GhChrA13G1936.1
GhPAO23	Gh_Sca005492G01	GhChrA06G2339.1
GhPAO23	Gh_Sca005492G01	GhChrD06G2285.1
GhPAO23	Gh_Sca005492G01	GhChrA05G3219.1
GhPAO23	Gh_Sca005492G01	GhChrD05G3102.1
