# Supplementary material for: Evolution and Expression Analysis of PAO Gene Family in Cotton: Focusing on Fiber Development and Stress Response
Source: Plants (Basel). 2026 May 7;15(10):1429. doi: 10.3390/plants15101429 (PMC13210522; doi:10.3390/plants15101429)
Supplement: Supplementary file 1 [file plants-15-01429-s001.zip › Supplementary Materials Table S3.pdf]

Table S3. Primers used in this study

Primer	Sequence (5'-3')
GhPAO10-qRT-F:	CCTGTTTTCCAGGTCCGTGCTT
GhPAO10-qRT-R:	GTCAGGCTTCTTCTCTTCCGCC
GhPAO21-qRT-F:	CAAAGCTTCCGGTTCTGCCTCT
GhPAO21-qRT-R:	GTCCGAAGCACGGATCTGGAAA
GhUBQ7-F:	CCGCATTAGGGCACTCTTTTC
GhUBQ7-R:	GGCATTCCACCTGACCAACAA
pLacZi-*pGhPAO21*-F:	tattggatcggaattcgaaaatcgagccattacaacaactcc
pLacZi-*pGhPAO21*-R:	gagcacatgcctcgagggattaagatcgggattaggatttag
JG4-5-*GhARR12*-F:	TGCCTCTCCCGAATTCATGATGGAGGAGAAAATGGGTG
JG4-5-*GhARR12*-R:	TCCAAAGCTTCTCGAGCATGCACGATCCAAGGGGG
JG4-5-*GhEDF1*-F:	TGCCTCTCCCGAATTCATGGCTATTTTCGTTAAAG
JG4-5-*GhEDF1*-R:	TCCAAAGCTTCTCGAGAGAAGCATGGCAACTTTTG
JG4-5-*GhVSR3*-F:	TGCCTCTCCCGAATTCATGGGATTACCAAATGCGAG
JG4-5-*GhVSR3*-R:	TCCAAAGCTTCTCGAGTCAACCTCCATACCAGAAATG
JG4-5-*GhSOT17*-F:	TGCCTCTCCCGAATTCATGTCGGCTCAACAAAATTTTC
JG4-5-*GhSOT17*-R:	TCCAAAGCTTCTCGAGCAAAGTTAAGCCTGAACC
JG4-5-*GhPLL18*-F:	TGCCTCTCCCGAATTCATGATCGACCAGTGTTTAAC
JG4-5-*GhPLL18*-R:	TCCAAAGCTTCTCGAGTATATATTCCATTACAAATTC
JG4-5-*GhCOR27*-F:	TGCCTCTCCCGAATTCATGGAGGGTTTCATCAGAAC
JG4-5-*GhCOR27*-R:	TCCAAAGCTTCTCGAGTGGAGTTTCCTTCTGTTTGC
JG4-5-*GhTGA1*-F:	TGCCTCTCCCGAATTCATGGAATCTCAGTTTGTTC
JG4-5-*GhTGA1*-R:	TCCAAAGCTTCTCGAGCTTGTGCTCGTGAACCTGCA
JG4-5-*GhHAT14*-F:	TGCCTCTCCCGAATTCATGGAGTTAGCCTTGAGCTTG
JG4-5-*GhHAT14*-R:	TCCAAAGCTTCTCGAGTGTGTTTGAAATAGAGGAG
0800-*pGhPAO21*-F:	ctatagggcgaattgggtacctagaaaatcgagccattacaacaactcc
0800-*pGhPAO21*-R:	aagcttatcgataccgtcgacggattaagatcgggattaggatttag
62SK-*GhTGA1*-F:	AAGCTTATCGATACCGTCGACATGGAATCTCAGTTTGTTC
62SK-*GhTGA1*-R:	TCAGCGTACCGAATTGGTACCTGCAGGTTCACGAGCACAAG
