# Supplementary material for: Evolution and Expression Analysis of PAO Gene Family in Cotton: Focusing on Fiber Development and Stress Response
Source: Plants (Basel). 2026 May 7;15(10):1429. doi: 10.3390/plants15101429 (PMC13210522; doi:10.3390/plants15101429)
Supplement: Supplementary file 1 [file plants-15-01429-s001.zip › Supplementary Materials Table S4.pdf]

Table S4. List of the conserved motifs of the *GhPAO* proteins

Motif	Length	Amino Acid Sequence
Motif 1	29	GRLFFAGEATTRRHPGTVHGAYLSGLREA
Motif 2	29	VTRWGNDPFSLGSYSYVAVGSSGDDYDIL
Motif 3	29	GVLKAGSIKFEPPLPQWKTDAIQRLGFGV
Motif 4	29	VIIIGAGLAGLAAARKLARFGFKVVVLEG
Motif 5	39	DDPYEMGGDHCFIPGGNSRVVEALAEGLPIHYGHTVTNI
Motif 6	41	YSGGPVLVALVAGKAAIDFESLSDEEIVNGVVSILRGIFGP
Motif 7	29	VFWGNELDTFGVLTEDADSRGEFFLFYSY
Motif 8	21	KVVTEDGSVFEADAVIVTVPL
Motif 9	21	VSVAVDLGASWITGVGGNPSG
Motif 10	29	DGQKVPADLDSKVETEFNKLLDKAVKLRQ
