# Supplementary material for: Evolution and Expression Analysis of PAO Gene Family in Cotton: Focusing on Fiber Development and Stress Response
Source: Plants (Basel). 2026 May 7;15(10):1429. doi: 10.3390/plants15101429 (PMC13210522; doi:10.3390/plants15101429)
Supplement: Supplementary file 1 [file plants-15-01429-s001.zip › Supplementary Materials Table S5.pdf]

Table S5. Information on candidate upstream regulatory factors targeting the TGACG motif in the *GhPAO21* promoter.

ID	Symbol	Description
Gh_D08G2618	ARR12	Encodes an *Arabidopsis* response regulator (ARR) protein that acts in concert with other type-B ARRs in the cytokinin signaling pathway.
Gh_A04G1448	EDF1, TEM1	Encodes a member of the RAV transcription factor family that contains AP2 and B3 binding domains.
Gh_A01G0091	VSR3, BP80-2	Encodes a vacuolar sorting receptor that participates in vacuolar sorting in vegetative tissues and in seeds.
Gh_A01G1833	SOT17, ST5C	Encodes a desulfoglucosinolate sulfotransferase, involved in the final step of glucosinolate core structure biosynthesis.
Gh_A01G0509	PLL18	Encodes a pectate lyase involved in response to nematodes.
Gh_A01G0983	COR27	cold regulated gene 27
Gh_A10G0769	TGA1	Encodes TGA1, a redox-controlled regulator of systemic acquired resistance.
Gh_D04G0859	HAT14	Homeobox-leucine zipper protein.
