# Supplementary material for: Evolution and Expression Analysis of PAO Gene Family in Cotton: Focusing on Fiber Development and Stress Response
Source: Plants (Basel). 2026 May 7;15(10):1429. doi: 10.3390/plants15101429 (PMC13210522; doi:10.3390/plants15101429)
Supplement: Supplementary file 1 [file plants-15-01429-s001.zip › Supplementary Materials Figure S2.pdf]

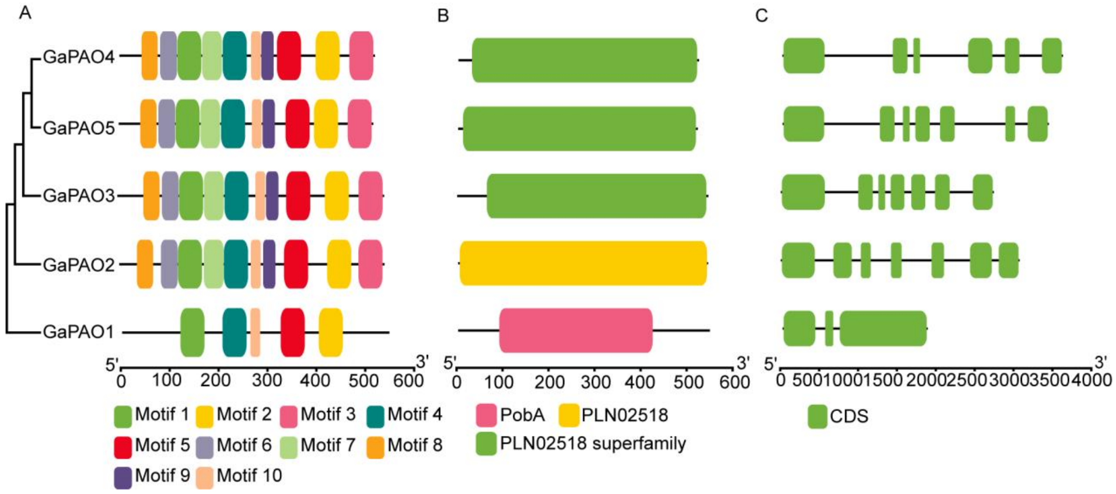


**Figure S2.** The conserved motif of GaPAO proteins. Distinct motifs are denoted by variant colors to ensure easy recognition. (**A**) Based on GaPAO protein sequences constructed a phylogenetic tree. The distribution of conserved motifs identified by MEME is depicted, with each distinct conserved motif marked by a colored box for clear visualization. (**B**) The figure illustrates the spatial distribution of conserved domains, with each distinct color block serving as a visual identifier for the corresponding conserved domain. (**C**) The gene structure of GaPAO is delineated, CDS are visually represented as green boxes.
