# Supplementary material for: Evolution and Expression Analysis of PAO Gene Family in Cotton: Focusing on Fiber Development and Stress Response
Source: Plants (Basel). 2026 May 7;15(10):1429. doi: 10.3390/plants15101429 (PMC13210522; doi:10.3390/plants15101429)
Supplement: Supplementary file 1 [file plants-15-01429-s001.zip › Supplementary Materials Figure S3.pdf]

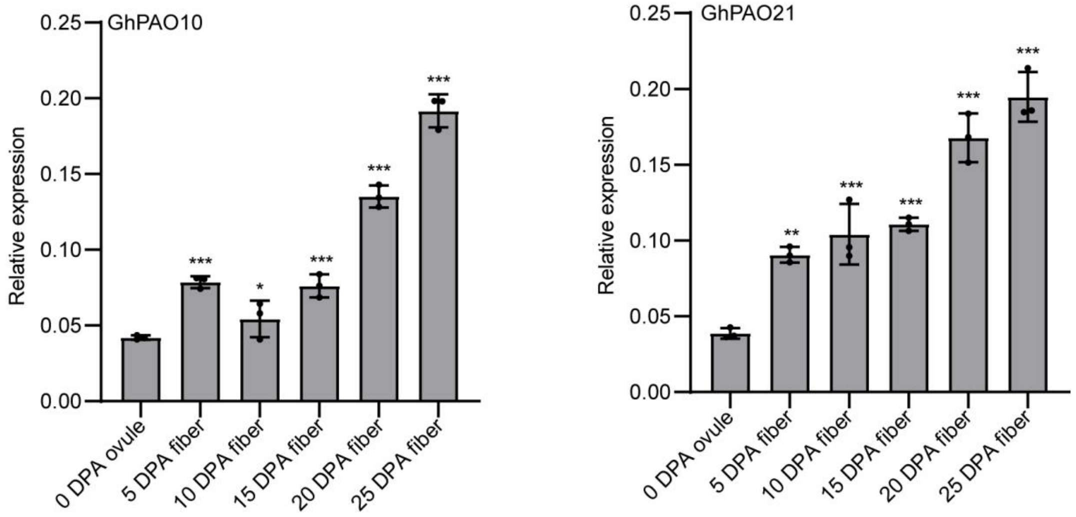

Figure S3. Expression of *GhPAO10* and *GhPAO21* genes at different stages of cotton development. Expression of *GhPAO10* and *GhPAO21* is normalized based on the expression of *GhUBQ7*. Error bars represent ± SD (n = 3). Asterisks indicate signiffcant differences by t-test; **$p \leq 0.01$; ***$p \leq 0.001$.
