# Supplementary material for: Evolution and Expression Analysis of PAO Gene Family in Cotton: Focusing on Fiber Development and Stress Response
Source: Plants (Basel). 2026 May 7;15(10):1429. doi: 10.3390/plants15101429 (PMC13210522; doi:10.3390/plants15101429)
Supplement: Supplementary file 1 [file plants-15-01429-s001.zip › Supplementary Materials File S1.pdf]

Supplementary Materials File S1

>GhPAO1
ATGGATGGTGGTGGGGGCTCTAAGAAAAGATTAAAGGTCACAGCGGCGGAGGTTGGTGTT
GATTCAGATGATGATGAGCCCATTTTGTCTTTGTTGAAGTTAAGGAAACCTAAGAATCCTAA
AAAGGATAAGGCTGGATTGGAAGGCAGTGCCGGGAAGTGCCAGAAGGTTGAAGTTAAAGC
AGGTAAAACTGTGGATAAAGATGAGGAGGATTTTGGGGGAATGAATGATACATTGGCCAG
CTTCAGAAAGAAGTTAAAGGATCCTAAAAAAGATATTGATCCAGGAGCAATGAGGGTAAG
GAGTTATTCTTTGAATAAGTCTGTGGAGGGTGGTGGAATTTTGGATGGGAAATCTGTGTCGA
ACACTGATGTGAAAGGTCAGGATATTGGTGAAGACAGGTCTGATGTGGCTAATGATAAAGT
TGTTGGAAAAAAGCGTACAGGGAAAGTAAGGAGAACCAAGTCAGATTCAAAAGCCACGC
CTAGTGAGGTTGATGATGAATCTGGAGCTAAACTTGAGGAAGATCAGAATGAGGGAGGTTT
GTTGCCTGGGGAAGGTTCGTATCAGTGTTCTCACAAGGCACAATCTGGTTCAGTAGGGAAA
TCTTGCGCAATTTTGAGTTTGAAACATAATTGCGAGGCTGCTCATCATGCTTCTGATTCAAA
GAATCCTAGCAGAAATTATGGTGATAGTTCTCATTCAGTTTCTAGCTCAAGCTTCTCACATT
CATCCTCCAAAGAATGCAACACAGCTGAGAATCAAGGATTTGACCATAGTTTGTGTCAACA
AGAAAGCATTTTGGAACCAGGTGACGTAACTGTTCAAAAGGATCCTACAGAGCATCCATGT
AGGTCATCTAAATTTTGTGACAAGGAAAGATATTGTCATTCCAACATTGAGCTCAGGGACA
ATTTCTCAGCAATTGACCAGAGGAGTAGACCAGGAAGTGAAAGTTCACAACAAAATAAAC
ATAATCTGTCACTGTCTGTTGTTGATTCACTGAATATGGAAGAAACTTGATGTTCCAAATTCT
TGCGCTGAGGAATACTCTTTAGAAACTTCCATTCATCCCAATGAACTTGTTGCCTCCATTCA
GAGGTGCAACTCTGCTCTGGATCAACCTTCTGAAGACGCAAGCCATGGTGCTTGTGGTCCC
AGCCATGATACTGTTTTCATCAGCAAAGAGGCCAATGTTGACTCTCCCATTTCAACACCTGA
TGAAAATGAAAGTTTCCATGAAGATGCAGTCTCTCTCCCTAGTTCAGAAATAAGAAACAGC
AAGTCATCAGCTGTCCAGCGAGGTGGGCGCAATATTAAAAAGCGTAGACACGGAGATATG
GCTTATGAAGGGGATGCTGATTGGGAAAATTTGCTAACCGAGCAAGGTTTTTTGGAAATC
AACAGTTTGTAGACAGTGATCATTCCTTTAGAGCAAGAGAGAAGTTTGATGAGGCAGCAGT
ATCATCTGGACTGAAAGCTCGTGCTGTGGGGCCAGTTGAGAAGATCAAATTTAAGGAGGTG
TTGAAGTGTAGAGGTGGGCTACAGGAATACTTGGAATGCAGGAATCATATTTTAGGTCTTT
GGAGCAAAGATGTTAACCGCATTTTGCCTCTTGTTGACTGTGGTGTTAGTGACACTCCTTCA
GAGGGTG
AACCATCCCGAGCTTCTCTAATTAGGAAGATATATGCATTTCTTGATCAGGGTGGTTACATA
AACTTTGGAATTTCTTCAAAGAAAGAGAAGGCTGAGCTTAGTGTTAAGGACAACTACAAAC
TTCTTGAGGGAAGAAGAAGTGATGGTAATTCTGTTGCCTCTGTTGCTGATTCAGAGGATGGA
GTTGCCTTTATCCTGGGTCAGGTCAAGAATTCTAAAGCTTCTATGGATGCAAAGACTGGTGT
TAGAGTTGTTGATGAAAACCAGGCATCTGAAGCCACAATAGCAATAGCTGAAGTATTGGTT
GATTCGATCACACCAAAATTACCTTATATATGCCAGCAAAATAGCAGCGTTAGTGCAAAAT
TAAACACTGGATTGATTAGTTCGCAGGTTTCAAGTACTGATCTATCTTGTGATGCAATTGAT
GTTGGAGTAGCCCCTGTAGTAACTCCAGAAGAAAGGAATGACTCACAGTATGTTCAATCTG
CAACTTATGATAAACCTGACGGGAATCATCAGCTGCTGAATGATTCAGAGGTCAGAAAGA
ACATCATAGTTATTGGAGCTGGTCCTGCTGGATTGACTGCTGCACGCCACTTGAAACGTCAG
GGATTTTCTGTAGTTGTACTTGAGGCTAGGGATAGGATAGGAGGTCGTGTTTATACTGATTG
CTCCTCTCTTTCAGTACCTGTGGATCTTGGGGCTAGCATTATTACTGGAGTTGAGGCTGATGT
GTCAACTAATAGAAGACCAGATCCATCCTCATTGATTTGTGCACAGTTGGGGCTAGAGTTA
ACTGTGTTGAATAGTTCCTGTCCTCTTTATGACATTGTATCTGGTCAAAAGGTTCCTGCTGAT
CTGGATGATGCTCTGGAAGCTGAATACAATAGTCTTCTTGACGATATGGTGTTTCTTTTTGCT
CAAAAAGGTAAAAAAGCAATGACAATTTCTCTTGAGGATGGTTTAGAATATGCCCTAAAA
AGGCATCGGATGGAAGAAATAGGAGTAGATATTGAAGACAGAATCACATTCTTCAGTGGA
TGCTTTCTATGACTCAAAAGAAAGCAATATCTTTGGATTTCCTGGAAAAAAACGTTCTGAA
GAGGAGATTTTGAGTCCTCTTGAGAGAAGGGTCATGAATTGGCACTATGCTCACTTGGAGT
ATGGCTGTGCTGCTTCGCTTAAGGAAGTTTCTCTTCCCAACTGGAATCAGGATGATGTTTAT
GGCGGCTTTGGAGGAGCCCATTGTATGATTAAAGGGGGTTACAGTACGGTGGTTGAGTCTC

TTGGAGAAGGGCTTCTGATCCACTTGAACCACGTAGTCACAAATATTTCATACAGCCCAAA
GGGCCCGGGGGTTGATAATAGTCATCATAGGCAGGTCAAAGTTTCCACATCAAATGGCAGT
GAGTTTTCAGGAGATGCTGTGCTTATCACTGTGCCACTTGGTTGCTTGAAAGCAGGAGCCAT
AAAGTTTTCTCCTCCGTTGCCCCAATGGAAACATTCTTCCATACAGCAGCTTGGTTTTGGAG
TACTTAATAAAGTCGTCTTGGAATTCCCAGAAGTTTTTTGGGATGATACTGTGGATTACTTTG
GAGTGACTGCTGAGGAAACAGATAGTAGAGGCCATTGTTTTATGTTTTGGAATGTCCGAAA
AACTGTTGGGGCTCCTGTTCTTATAGCCTTAGTGGCTGGTAAGGCAGCTATTGATGGTCAAA
ATATGAGCGCATCAGATCATGTAAACCATGCTGTAATTATTCTCCGTAAACTTTTTGGAGAG
GCTTCAGTTCCTGATCCTGTTGCCTCAGTTGTTACTGATTGGGGAAGGGATCCTTTTAGTTAT
GGCGCTTACTCCTATGTTGCCATAGGAGCATCTGGAGAAGACTATGATATGTTGGCCAGGC
CTGTTGAGAACTGCTTGTTTTTTGCTGGAGAAGCTACCTGCAAGGAGCATCCTGACACAGTT
GGTGGTGCAATGTTGAGTGGGCTTCGGGAGGCTGTGCGATTAATTGACATATTTACCACTGG
AAATGATTATACAGGAAGTAGAGGCAATGGAGGCTGCCCAGAGACATTCAGAATCAGGAA
GGGATGAAGTTAGGGACATAATTAAGAGACTTGAAGCAGTTGAACTTTCTAATGTCTTGTA
CAAAAACTCTTTGGATCGTGCTCGGGTTTTAAGCAGGGAAGCTTTACTGCGGGACATGTTCT
TTAATGTGAAAACCACTGCAGGACGATTGCATCTAGCCAAGAAGTTGTTAGGTCTCCCAGT
TGAATCCTTGAAATCCTTTGCTGGGACAAAGGAAGGGCTTAGCACACTCAACTCATGGATG
CTGGATTCAATGGGGAAAGATGGGACGCAACTGTTGCGCCATTGTGTTCGTCTTCTTGTGCT
TGTCTCAACTGATCTACTTGCAGTTCGTTCATCAGGCATAGGGAAAACTGTGAAGGAAAAA
ATTTGTGTGCATACAAGTCGTGATATACGTGCTATAGCAAGCCAGCTGGTTAATGTTTGGCT
TGAAGTCTTCCGTAAGGCAAAAGTTTCTTCAAAGAGAAAATCCCTTAAAGATCCAGCTTCA
GGAAAGCCACCTCTGCACTCACAGCATGGTGCTTTTGAGAGTAAAGAAAGCTTGCGGATCC
ATTTTCTGCTGGAAAGCAGTATCCTTTAAACATAAAGGAGAATGGCAAATCACTTGATATT
GAGGTGGAAGCTGTCAACCAAGGAATGTCAGAGGAAGAGCAGGCTGCCTTTGCTGCTGAA
GCAGCTGCCCGAGCTGCAGCAAAAGCAGCTGCAGAGGCACTTGCATCCACAGAAGCCAAT
TGCAACAAATTACTGCAGCTTCCTAAGATTCCTTCTTTTCACAAATTTGCCAGAAGGGAGCA
ATACGCACAAATGGATGAAGGGAAATGGCCTGGTAGTGTTTTTGGAAGACAAGATTGTATA
TCAGAAATTGACTCTAGGAACTGCAGAGTCAGGGACTGGTCTGTTGATTTCTCTGCTGCTTG
TGTTAACCTTGACAATTCCGGAATGTCAGTAGATAACCTGTCTCAGAGGAGCCACTTGAGC
TCAGAGAACATTCTGGAGAAAGTTTGGCTGTGGATAGCAGTATCTTCACGAAAGCATGGAT
TGATACTGCTGGTAATGGGGGGATCAAGGATTATCATGCCATTGAGAGATGGCAGTCTCAA
GCAGCTGCTGCTGATCCAGATTTCTTCCATCCTACAAATTTCAAGGATGAGGAAGATTCAA
ATACGAGTTCAAGGCAACCAACCTGGAAGAATGATGGACGAGCAAATGAGAGCTCCGTCT
CCCAAGTTTCTGTAAACAAGGAGCGATTTGAAAATCATCCCTGTGGAGCTGATCGTATTAA
ACAGGCTGTCGTTGATTATGTTGCATCATTGCTAATGCCCCTTTATAAGGCAAGAAAAAATTG
ATAAGGAGGGATACAAATCGATAATGAAAAAAACTGCAACAAAGGTAATGGAGCAGGCA
ACAGATGCAGAGAAAAACATGGCTGTTTTTGAATTTCTTGATTTCAAGCGCAAAAACAAGA
TTCGTCCCTTTGTAGACAAATTGATTGAGAGGCACATGGCAATGAAGCCAACCATGAATCT
ATGA

>GhPAO2
ATGGGAAAGAAGCCAAGAATTGTGATAATTGGAGCTGGGATGGCTGGTCTTACTGCAGCTA
ACAAGCTCTATACTTCCACTGGCTCCGACCATTTGTTTGAGCTTGTTGTTGTTGAAGGTGGTG
ATAGAATTGGTGGCAGAATCAACACTTCGGAGTTTTGTGGTGACAGAATTGAGATGGGTGC
TACTTGGATCCATGGTATAGGAGGCAGCCCGGTACATCAAATTGCTCGGGAAATCCATGCG
CTTGAGTCTGATAAGCCATGGGAGTGTATGGATGGGTTCTCGGGTGAGCCAAAGACTATTG
CTGAAGGTGGGTTCGAGCTAAATGCCTCCATCGTTGACCCCATATCCACCCTTTTCAAAAAC
CTGATGGATTTCGCTCAAGGGAAGCTGACTGAATACAGTGCAGGCAGCGGAGGAGATGCT
TGTTACTACAATTTTGCAGCTAAAGCAGCCTTGAAAGATTGTACGAGCAATGGTGGCTTTGG
TAACCAGAGTAGTGTCGGTGCGTTTCTTAGACGAGGCCTTGGTGCTTACTGGGATTCTTGCA
AGGACCGTGAGGAGCTGAACGGATATGGTAAATGGAGCAGAAAATTGCTTGAAGAAGCCG

TTTTTGCTATGCATGAAAACACCCAGAGAACTTATACTTCTGCCGGTGATCTGTTCAATCTA
GATTACGAGGCAGAAAGCGAGTACCGTATGTTTCCTGGTGAAGAAATCACCATTTCTAAAG
GCTATTTGAGCATAATTGAACATCTTGCATCTGTTCTTCCTCCTGGCGTAATCCAATTAGGCC
GCAAAGTCACAAGAATCGAATGGCAACCTGAGGGTCATAAATCAATACAAGTTCCAAATG
GCTATGATTCCAGACCAGTGAAGATTGAGTTTTGTGATGGATCTTCTATGTTAGCAGATCAT
GTGATAGTCACAGTTTCATTAGGGGTCTTAAAATCTGGAACTGGTCAAGATTCGGGTATGTT
CAATCCTCCCCTTCCTACTTTCAAGACAGAGGCTATATCAAGACTTGGATATGGAGTTGTTA
ACAAGCTGTTCCTTCAATGGAGTCCAAATGGTAATCGACCCGCCAATGATAAAGAGAAGTT
CCCTTCCTTGCAAATTGTTTTCCATCCCCCAGAATCCGAGTTAAGGCATGAAAAGATCCCAG
GGTGGATGAGGAGGACAGCTTCACTGTCTCCTATTTATAACAATTCAAGCGTCCTCCTATCC
TGGTTTGCAGGTAAAGAAGCACTTGAGCTTGAAACACTTAGCGATGAAGAGATTATAAATG
GAGTTTCAGCAACAGTATCTGGTTTATTACCAGTATCAAAACACCACAAGGAAGACAAGTA
TAATTCCCCTGAATTCTGCAATGGGAATGTGGAGAGCTGTGATGACAATGGAGTGAGATTT
GGTAAGGTTTTGAAGAGCAAATGGGGCAGTGATCCATTATTCTTGGGATCTTACAGCTACGT
GGCTGTTGGATCAAGCGGTGCTGATTTAGACACAATGGCTGAACCCTTACCAAAGCTTGGG
AGCACTGACTCAGACCACCATCCACTTCAAATATTGTTTGCTGGGGAGGCTACACACAGAA
CCCACTATTCTACAACCCATGGAGCTTATTTCAGTGGTCTTAGGGAAGCCAATAGGCTTCTC
AAACATTATCGTTGTGTTGGGGTTTAG

>GhPAO3
ATGGAGCCTCCCCAAGATACTTCCGAGAACCCTAACGATGTCCTTTCCGACGACGACTCTT
CACCGGAAAACACCAATCCCGACGATCAAGAAATCCCCAGTACGACACTCGACCCACCCA
TTTCCGATACCCAAGATGAATCCTCCGATCCCGTCCCCGACGAGCAACCCGAAAACACTAA
TTCGAACCCCGCCGAGCCTGGTCCACCTGCACGCAAGCGCCGCCGCAGAAAGCGTTTCTTT
ACAGAACTCATCGCCAATCCATCCTTCTCCAAGAACCGGCGCCCTAGAATATCGGGCCTAG
CTAGAGAAATGGACACCGAAGCTTTAATCGCGATCTCCGTTGGTTTCCCTGTTGATTCTCTT
ACCGAAGAAGAAATCGAAGCCAACGTGGTGTCCAGAATCGGAGGCCAAGAGCAAGCCAA
CTACATCGTTGTAAGAAATCACATTCTGGCTCGCTGGAGATCCAATGTATCCGTCTGGCTGA
CGCGCGAGCACGCCCTCGAATCAATCCGAGCTGAGCACAAGAACCTAGTGAACGCAGCAT
ACAATTTCCTTCTCGAACACGGTTACATTAATTTCGGTTTAGCCCCGGCTGTTAAAGAAGCG
AAATTGAAGTCTTTTGATGGTGTAGAAAGAGCCAATGTGGTGATTGTGGGTGCGGGTCTTTC
CGGTTTGGTCGCGGCGAGGCAATTAGTTTCCATGGGGTTTAAAGTTGTCATCTTGGAAGGTA
GGACGCGCCCTGGAGGGCGCGTAAAGACAAGGAAGATGAAAGGTGATGGGGTGGTGGCT
GCAGCGGATCTTGGTGGGAGTGTTCTCACGGGAATAAATGGAAATCCACTTGGGGTTCTTG
CAAGGCAAATGGGATTACCGCTTCATAAGGTGCGAGATATTTGTCCTTTGTATTTGCCAGAT
GGAAAGGCCGTAGATGCTGATGTTGATTCTAGGATAGAGGTTTCATTTAATAAGCTATTGG
ATAGGGTTTGTAAACTTAGGCATTCTATGATTGAGGAAGTTAAATCAGTTGATGTTCCATTA
GGGACAGCATTAGAAGCCTTTAGGAGTGTTTACAAGTTTGCTGAGGATTCACAGGAGAGCA
TGTTGTTGAATTGGCATCTTGCTAACCTTGAATATGCTAATGCTTCTTTGATGGCTAATTTGT
CTATGGCCTATTGGGATCAAGATGATCCATATGAGATGGGCGGCGATCACTGTTTCATACC
CGGTGGCAATGAGAGGTTTGTTCGAGCACTTGCGGAGGACCTTCCCATTTTCTATGGGAGG
ACTGTGCAGAGTATCAGGTATGGTATCGATGGTGTTAGGGTTTACGCCGGTGGGCAGGAGT
TTTGTGGGGATATGGCTCTTTGCACTGTTCCATTAGGAGTTCTCAAGAAGGGATCGATAGAA
TTTGTTCCTGAGCTTCCGCAAAGAAAGAAGGATGCCATTCAGAGACTGGGATTTGGGTTGC
TGAATAAGGTTGCTATGTTGTTTCCATACAATTTTTGGGGCGGAGAGATTGATACTTTTGGC
CATCTGACAGAAGACCCAAGTATGAGAGGCGAGTTCTTTTTGTTTTATAGCTATTCTTCTGT
GTCAGGTGGTCCACTCCTTGTTGCTCTAGTTGCCGGAGATGCAGCAATCAAGTTTGAACTGA
TGTCTCCTGTTGAGTCTGTGGAAAGGGTTTTAAACATATTGCGAGGCATTTTTCATCCAAAA
GGGATTGTTGTACCAGATCCTGTCCAGGCTGTTTGTACCCGGTGGGGAAAGGATCGCTTCAC
TTATGGATCCTACTCTCATGTTGCTATTGGTTCATCCGGGGATGATTATGATATTCTAGCTGA
GAGTGTTGGAGATGGGAGAGTCTTCTTTGCTGGTGAGGCAACTAATAAGCAGTATCCTGCC

ACAATGCATGGAGCCTTTTTAAGTGGCATGAGAGAGGCCGCTAACATGCTTAGAGTGGCCA
GGAGGAGGTCATTGATTCTATCTGACAAAGTTAATAACGACTTGGAGAAATGTGATACTTT
GAATAAGTTGTTCGAGAACCCTGACCTGACATTCGGGAGCTTCTCAGCTTTGTTTGATCCCC
ATTCTAATGATGTTGGATCGCATGCATTTATAAGGGTCAAATTCCATGGGGATAAATTAAAC
TCGAGTCACTTGTGTCTTTATGGCTTGATTACGAAGAAGCAAGCCATTCAGTTAAGTGAAAT
GAATGGAGATGGGAACAGGATGAATTCGTTGCATCGTGACTTTGGGGTGAAGTTGGTTGGT
GGTAAAGGGTTATCAAATGTTGCGGAGTTGCTGATATCACGCATCAAAGCAGCTAAACCAA
CCTAA

>GhPAO4
ATGAATCCACCAAATGAAACCCTGGATGATTTCTCTCAATTCCCTCTTCCCCATTTTACTCTC
ACTCCTCCCTTACCAAATCCTACCTCTATTCCTAATTTCCATCCAATCCCAATCCTTAACCCG
AATGCCGCTCCTCCTCTCAATGATCATCTAATTTCCTTTCCAACCCCCAAAAAACGACGACG
CGGCCGACCTCAACGCAGTGGCGCGACGTCGGCGTATCAGTTCCTTACCTTCACCAATGGC
TCCTTCAGCCCCAACCTCCCGAACTCTAATCCTAACCTTGACCTTAATTCAATAACCTCATC
ATCAGCGGCGACGGAACAAACTACACAACCCAAAATTGACGACGAGATCATTTTGATCAG
TAAGGAATCAACGGCAGAGGCTCTCACCGCTCTTTCTGCTGGATTCCCTGCAGATTCCCTCA
CCGAGGAAGAAATTGATTTCGGCGTCATTTCCTCCATTGGTGGCATCGAGCAGGTAAATTAT
ATTCTCATTCGAAATCACATTATTGCAAAATGGCGCGAAAATGCATCCAATTGGGTGGCTA
AAGACATGCTTGTTGATTCTATACCGAAACATTGTAGCACGATTTTAAATTCTGCATATAAT
TATTTAGTTACGTATGGATATATAAATTTTGGGATTGCCCCTGCAATTAAGGAAAAAATTCC
TGCGGAACCGACTAGAAGTAATGTGGTTATTATTGGTACCGGGTTAGCAGGACTGGCTGCG
GCTAGACAGTTAATGAGGTTTGGGTTTAAGGTGACGGTTCTGGAAGGGAGGAAACGAGCC
GGCGGGAGGGTTTATACAAAGAAGATGGAGGGAGGGAATAGGGTGAGTGCAGCTGCTGAT
TTAGGTGGGAGTGTGTTAACAGGTACCTTGGGGAATCCGTTAGGGATTCTGGCGAAACAAT
TGGGTTCTTCTCTTTTTAAGGTGAGGGATAAATGTCCACTTTACAGGACAGATGGGAGTCCG
GTGGATCCGGATATGGATATGAAGGTGGAGATGGCCTTTAATAGGCTTTTGGATAAAGCTA
GCGAGCTTAGGCAGTTGATGGGGGAGGTTTCTACGGATGTTTCACTTGGGGCTGCATTAGA
GACATTTAGAGAGGTTTATAGGGATGCAGTGACTGAAGAAGAGATTAATTTGTTCAATTGG
CATCTTGCAAATTTAGAATATGCAAATGCCGGATTGGTTTCAAAGCTTTCACTTGCATTCTG
GGACCAAGATGATCCATATGATATGGGAGGGGATCATTGTTTCTTGCCTGGAGGAAATGGA
AGGTTGGTTCAAGCTCTGGCCGAGAATGTGCCTATTTTGTACGAAAAAACTGTGCATACTAT
CAATTATGGCAATGATGGAGTGCAGGTTATGACAGGAAGTCAGGTGTATGAAGGTGATATG
GCATTATGTACGGTACCTCTCGGAGTTCTAAAGAGTGGGTCAATCAAGTTTGTCCCAGAGTT
GCCTCAGAGGAAGCTTGATGGAATAAAGAGGTTGGGATTTGGGTTATTGAACAAGGTTGGA
ATGCTTTTTCCTTATGTATTTTGGGGTACAGACTTTGATACCTTTGGGCATCTTACTAATGAT
CCAAGCCGTCGAGGGGAGTTTTTTCTGTTCTATAGCTATGCAACAGTTTCCGGTGGTCCTCT
ATTGCTTGCTTTAGTAGCAGGAGAAGCTGCACACAGGTTTGAGAGTCTGCCTCCTATAGATG
TTGTGGCCCAGGTTCTCCAAATTCTCAAGGGTATATATGAACCACAGGGTATCACTGTCCCC
GAGCCCCTCCAAACTGTCTGTACCAGATGGGGTGGTGATCCCTTCAGCCTAGGTTCATACTC
TAATGTTGCAGTGGGAGCATCCGGGGATGACTATGATATATTAGCTGAAAGTGTGGGGGAC
GGAAGACTTTTCTTTGCAGGGGAGGCCACTACAAGGCGATACCCTGCCACCATGCATGGAG
CTTTTCTTACTGGACTTCGGGAAGCTGCAAATATGGCTCAATATGCAAAGTCTCGGACTGCA
AAGAAAAAGATCAACAGGAGTCCATCAAGTAATGCTCATTCTTATGCTTCTGCCCTTATGG
ATTTATTCAGAGAGCCTGATCTGGAATTTGGGAGTTTTTCTGTTATTTTTTGTCAAAAGAATG
CTAATCCGAAGTCACCAGCCATTCTAAGGGTGAAAATTAGTGAGCCCCGAAAGAGGAATC
TGGAAAGCTCAAAGACAGATCAGCAACATTCGAATAAGGTGCTTTTTCAGCAGCTCCAATC
ACATTTTAATCAGCAACAACAGTTGCATGTTTATACATTGTTATCAAAGAAACAGGCATTTG
AGCTGAGAGAAGTGAGAGGTGGTGATGAGATGAGGTTGAACTATCTGTGCGAAAAGCTGG
GAATTAAGCTGGTGGGACGTAAGGGTTTGGGACCTACTGCCGATTCCATCATTGCTTCTATT

AAAGCACAGAAGGGCGTTCGAAAACCTTCTTCAACTCCTTTGGCTCCAAAATCAGGGACAT
CAATGCTGAAAATTGGCACTTTAAAGCAAAAGTTCATCAGGTAA

>GhPAO5
ATGGAAAGTGATGACGGTTCAAACTCCAAGGCAGGTCCACAATTGCAATTTGATTTGGGGA
ATAATGTTGAGCTAGGGCTAGAAAAGGTTACATTACAGAATTCTTTGAAACTTAGGAACAA
GAAAATGGATGGTGGTGGGGGCTCTAAGAAAAGACTAAAAGTAACAGCGGTGGAGGTTGA
TGTTGATTCAGATGATGATGAGCCCATTTTGTCCTTGTTGAAGTTAAGAAAACCCAAGAATC
CTAAAAAGGATAAGGCTGGTTTGGAAGGCAGCGCTGGGAAGTGCAAGAAGGTTGAAGTTA
AAGCAGTTAAAACTGAGGGCAAGAATGAGGAGGATTTGGGGGGAATGAATGATACGTTGG
CCAGCTTTAGAAAGAAGCTAAAGGATCCCAAGAAAGATGTTGATCCAGGAGCAAAGAGG
GAAAGGGATTATTCTTTGAATAAGTCTGTGGAGGGTGGTGTAGTTTTGGATGGGAAATCTGT
GTCGAACACTGGTGTGAAAGGTCAGGATATTGGTGAAGACAGGTCTGATGCGGTTACTGAT
ACAGTTTTCGAAAGAAAGCATACAGGGAAAGTAAGGAGAGCCAAGTTTGATTCAAAATCC
AAGCTCATCGAGGTTGATGATGAATCCAGAGCTAAGCTAGAGGAAGATCAGAATGAGGGA
GGTTTGTCGCCTGAGGGTGGTTTGAATCAACATTCTCACGAGGCACAATCTGATTCAGTGAG
GAAATCTTGCCCAATTACGAGTTTGAAACATAATTGCAAGGGTTCCCATCATGCTTCTGCTT
CAAAGAATCCTAGCAGAAATTATGGTGATAGGTCTCATTCAGATTCTAGTTCAAGCTTCTCA
CATTCATTCTCCAAAGAATGCAACACAGCTGAGAATCAAGGATTTGGCCATAGTGTGTGTC
AACAAGAAAGCATTTTGGAACCAGGTGACTTAAATGTTCAAAAGGGTCCTTCAGGGGATCC
ATGTAGGTCACCTAAAGTTTGTGACAAAGAAAAATATGGGCATTCCAACATTGAGCTCAGG
GACAATTGCTCAGCAGTTGACCAGAGGAATAAGCCAGAAAGTGGAGGTTCACGACAAACT
AAACATAACCTGTTACCGTCTGTTGTTGATTCACTGAAGATGGAAGATACTTGCACTGATGT
TCCAAATGCTTGCGCTGAGGAAAACTCTTTAGAAGACTCTGTTCATCCCAATGAATTTGTTG
CCTCCATTCAGAGGTGCAACTCTGCTCTCCGTCAACCTTCTGAAGATGCATGCCATGGTGCG
TGTGGTCCCAGCCATGATACTCTTTTCATCAGCAAAGAGGCCAATGTTGATTCTCCCACATC
AACACCTGATGAAAATGAAAGTTTTCATGAAGATGCAATCTCTCTCCCCAGTTCTGAAATC
AAAGACAGTATGTCATCAGCTGTACAGCGAGGTGGGCGCAGTATTAAAAAGCGTAGACAT
GGAGATATGGCTTATGAAGGGGATGCTGATTGGGAGAATTTGCTAAATGAGCAAGGTTTT
TTGGAAATCAACAGTTTGCAGACAGTGATCGTTCCTTTAGAGCAAAAGAGAAGTTTGATGA
GGCAGCAGTATCATCTGGACTGAAAGCTCGTGCTGTGGGACCAGTTGAGAAGATCAAATTT
AAGGAGGTCTTGAAGGGTAGAGGTGGGCTACAGGAATACTTGGAATGCAGGAATCATATC
TTAGGTCTTTGGAGTAAAGATGTTAACCGCATTTTGCCTCTTGCTGAGTGTGGTGTTACTGAC
ACTCCTTCAGAGGGTGAACCACCCCGAGCTTCCCTAATCAGGGAGATATATGCATTTCTTG
ATCAGGGTGGTTACATAAACTTTGGAATTGCTTCAAAGAAAGAAAAAGCTGAGCTTAGGGT
TAAGGATAACCGCAAGCTTCTAAAGGAAAGAAAAAATTATGGCAATTCTGTGGCCTCTGTT
GCTGATTCAGAGGATGGAGTTGCCTTCATCCTTGGCCAAGTCAAGAATTCTGAAGCCTCAA
TGGATGCAAAGATCAGTGTTAGAGTTGATGATGAAAACCAGGCATCTGAAGCCACAATAC
CTGAAGTGTTGGTTGATTCGATCACATCAGAATTACCTTGCAGAAAAGAACAAAAGGAAC
ACCCAAGTGATAATTGCCAGCAAAATGGCAGCATCAGTGCAAAACTAAACCCTTTATTGAT
TAGTTCGCAGGTTCCAAGTGCAGATCTATCTTGTGATGCTATTGACATGGGAATAGCCCCTG
TAGTAACTCCAGAAGAAAGAAATGACTCTCATTATGTTCAGTCTGCAACTTATGATAAACC
TGATGGGAATCATCAACTGCAGGGTGATTCAGAGGTCAGAAAGAACATCATAATTGTTGGA
GCTGGTCCTGCTGGATTGACTGCTGCACGCCACTTGAAACGTCAGGGATTTTCTGTAGTAGT
ACTTGAGGCTAGGGATAGGATAGGAGGTCGTGTTTATACTGATTGCTCCTCTCTTTCAGTAC
CTGTGGATCTTGGGGCTAGCATTATTACTGGAGTTGAGGCTGATGTGTCAACTAATAGAAG
ACCAGATCCATCCTCACTGATTTGTGCACAGTTGGGGCTAGAGTTGACTGTGTTGAATAGTT
CCTGTCCTCTTTATGACATTGTATCTGGTCAAAAGGTTCCTGCTGATCTGGATGATGCTCTGG
AAGCTGAATACAATAGTCTTCTTGATGATATGGTGTTTCTTGTTGCTCAAAAAGGTCAAAAA
GCAATGACAATTTCTCTTGAGGATGGTTTAGAATTTGCCCTAAAAAGGCATCGGATGGAAG
AAATAGGAGCTGATATTGAAGAAATAGAATCACATTCTTCAGTGGATGCTGTCTATGACTT

GAAAGCAAGCAATGGAAAAAAATGTTCTGAAGGGGAGATTTTGAGTCCTCTTGAGAGAAG
GGTTATGAATTGGCACTATGCCCACTTGGAGTATGGCTGTGCTGCTCCGCTTAAGGAAGTGT
CTCTTCCCAATTGGAATCAAGATGATGTTTATGGCGGCTTTGGAGGAGCCCATTGTATGATT
AAAGGAGGTTACAGTAAGGTGGTTGAGTCTCTTGGAGAAGGACTTCTGATCCACTTGAGCC
ATGTAGTCACAAATATTTCATACGGCCCAAAGGACCCTGGGATTGATAATAGTCATCATAG
GCAGGTCAAAGTTTCAACGTCAAATGGCAGTGAATTTTCAGGAGATGCTGTGCTGATCACT
GTGCCACTTGGTTGCTTGAAAGCAGGAGCCATAAAGTTTTCTCCTCCATTGCCCCAATGGAA
ACATTCTTCCATACAGCAACTTGGTTTTGGAGTACTTAATAAAGTTGTTTTGGAATTTCCAGA
AGTTTTTTGGGATGATACTGTGGATTACTTTGGAGTGACTGCTGAGGAAACAGATAGTAGA
GGCCATTGCTTTATGTTTTGGAATGTCCGAAAAACTGTTGGGGCTCCTGTTCTTATAGCCTTA
GTGGCTGGTAAGGCAGCTATTGATGGTCAAACTATGAGCTCATCAGATCATGTAAACCATG
CTGTACTTATTCTCCGAAAACTTTTTGGTGAGGCTTCAGTTCCTGATCCTGTTGCCTCAGTTG
TAACTGATTGGGGAAGGGATCCTTTCAGTTACGGTGCTTACTCCTATGTTGCCATTGGAGCA
TCTGGAGAAGACTATGATATGCTGGGCAGGCCTGTTGAGAACTGCTTGTTTTTTGCTGGAGA
AGCTACCTGCAAGGAGCATCCTGACACAGTTGGTGGTGCAATGTTGAGTGGACTTCGGGAG
GCTGTGCGATTAATTGACATATTTACCACTGGAAATGATTATACAGCTGAAGTAGAGGCAA
TGGAGGGTGCACAGAGACGATCAGAATCAGGAAGGGATGAAGTTAGGGACATAATTAAG
AGACTTGAAGCAGTTGAACTTTCTAATGTCTTGTACAAAAACTCTTTGGATCGTGCTTGGGT
TTTGAGCAGGGAAGCTTTACTACAGGACATGTTCTTTAATGTGAAAACCACTTCAGGACGA
CTGCATCTAGCCAAAAAATTGTTGGGTCTCCCAGTTGAATCCTTGAAATCCTTTGCTGGGAC
AAAGGAAGGGCTTAGCACACTCAACTCATGGATGCTGGATTCGATGGGGAAAGATGGGAC
TCAGTTGTTGCGCCATTGTGTTCGTCTTCTTGTGCTTGTTTCAACTGATCTACTTGCAGTTCGT
TCATCAGGCATAGGGAAAACTGTGAAGGAAAAAATTTGTGTGCATACAAGTCGTGATATAC
GTGCTATAGCAGCCAGCTGGTTAGTGTTTGGCTTGAAGTCTTCCGTAAGGCAAAAGCTTCTT
CAAAGAGAAAACCCCTTAAAGATACTGCTTCAGGAAAGCCGCCTCTACACTCACAACATT
GTGCTTTTGAGAGTAAAGCAAGCTTGCAGGATCCATTTCCTGCTGGAAAGCAGTATCCTTTC
TATGCAAAAGAGAATGGCAAATCGGTTGATATGGAGGTGGAATCTGTCAACCAAGGAATG
TCAGAGGAAGAGCAGGCTGCCTTTGCTGCTGAAGCAGCTGCCCGAGCTGCAGCAAAAGCA
GCTGCAGAGGCACTTGCATCCACAGAAGCCAACTGCAACAAATTGCTGCAGCTTCCTAAAA
TTCCTTCTTTTCACAAATTTGCCAGAAGGGAGCAATATGCACAAATGGATGAAGGGAAATG
GCCTGGTGGTGTTTTAGGAAGACAAGATTGTATATCAGAAATAGATTCTAGGAACTGCAGA
GTCAGAGACTGGTCTGTTGATTTCTCTGCTGCTTGTGTTAACCTTGACAGTTCCAGAATGTCA
GTAGATAACCTGTCTCAGAGGAGCCACTTGAAGCTTAGAGAACACTCTGGAGAAAGTTTGG
CTGTGGACAGTAGTATCTTCACAAAAGCATGGGTTGAGAATGCTGGTAGTGAGGGGATTAA
GGATTGTCATGCCATTGAGAGATGGCAGTCTCAAGCAGCTGCTGCTGATCCAGATTTCTTCC
ATCCTACAAATTTCAAGGATGAGGAAGATTCAAATGCTAGTTCAAGGCAAACAACCTGGA
AGCATGATGGACGAGCAAATGAGAGCTCCATCTCCCAAGTTTCTGTTAACAAGGAGCGATT
TGAAAATCATCCCCATGGTACTGATCGTATTAAGCAGGCTGTCGTTGATTATGTTGCATCAT
TGCTAATGCCCCTTTATAAGGCAAGAAAAATTGATAAGGAGGGATACAAATCGATAATGA
AGAAAACTGCGACAAAGGTAATGGAGCAGGCAACAGATGCAGAGAAAAACATGGCTGTT
TCTGAATTTCTAGATTTCAAGCGCAAAAATAAGATTCGCCCCTTTGTAGACAAATTGATTGA
GAGGCACATGGCAATGAAGCCAATCATGAAACCATGA

>GhPAO6
ATGGCTAAGCCGAGGATTGTGATAATTGGAGCAGGAATGGCTGGTCTTGCAGCAGCCAAC
AAGCTTTACACTAGTTCAAATGACTTGTTTGAACTATTTGTTGTTGAAGGTGGTACTAGAATT
GGGGGAAGGATCAATACATCAGAGTTTTATGGTGATAGAGTTGAAATGGGTGCTACTTGGA
TTCATGGTATAAAAGGTAGCCCAGTTCATCAAATTGCTCAACAAATAAATGCATTACAAGG
GTCTGATAAGCCATGGGAGTGTATGGATGGGTTACTTGATGAACCAAAGACCATTGCTGAA
GGTGGGTTCGAGCTAAATGGCTCTATGATTGAACCCATATCGACACTTTTTAAGAACTTGAT
GGATTTTGCTCAAGGCAATGAAGCATCCAAATGGTGTTCGATGAGTTGTCGCTTACGTAAC

AAAAGTGTTGGTTCTTTTTTAAGAAAAGGCCTTGATGTGTATTGGGATTCTAGTAAAGACCG
TGAAGAGCTTAAAGGGTATGGTAAATGGAGTAGAGAGTTGCTTGAAGAAGCCATTTTTGCA
ATGTATGAGAACACACAAAGGACTTATACATCAGCTGGTGATCTTTTCAGTTTAGATTATGA
AGCAGAAAGTGAGTACCGTATGTTCCCTGGTGAAGAAATCACTATTGGTAACGGATATTCC
AGTATAATCGAATACCTCGCGTCGGTACTCCCACGGGACGTAATCCAATTAGACCGAAAAG
TCGCTAAAATCGAATGGGATCGTTGTGATTCGAGGCCTGTGAAGATACACTTCTTGGATGG
ATCTTTTGTGGTAGCTGATCATGTTATTGTCACAGTTTCGTTAGGGGTTTTAAAAGCTGGTAT
TTGTAACGCTTCAGGTTTGTTTAGTCCTCCACTTCCTTCTTTCAAAACAGATGCTATATCAAG
ACTTGGATATGGTGTTGTTAACAAGCTGTTTCTCCGATTAAACGGTAATCGAAAACCCGAA
GAGCTCCCTTCCTTGCAAATGGTGTTTCATCGTTCGGATTCCGAGTTAAGGCATAAAAAGAT
CCCATGGTGGATGAGAAGGACAGCTACTTTATCCCCTATTTACAACAATGCAAGTGTGTTCC
TATCTTGGTTTGCAGGGAAAGAAGCTCTTGAACTCGAAAGACTTAGCAACGAAGAGATTAT
AAACGCGGTAACAACGACAGTTTCGAGTTTATTATCGAAACCCCATAATGAAATCATGTCT
GATAGCAACTCCAATGGATTTGAAGTGAGTTTTGTTGATGTATTGAAGAGCAAATGGGGGA
GTGATCCATTGTTCTTAGGGTCATACAGTTATGTTGCTGTTGGATCGAGTGGTGCTGATTTTG
ACACCATGGCTGAACCATTACCTACTGATGTGTATCATCATCGTCCCCTTCAAATTTTGTTTG
CTGGGGAAGCTACACATAGAACTCACTATTCCACAACTCATGGAGCGTACTTTAGTGGTAT
TAGGGAAGCCAATAGGCTTCTTCAACATTATCATTGTGTTGGGGTTTAA

>GhPAO7
ATGGATCTGCTCCGCTTCCAAGGCATTGAGAGGCAGGAGGAGGCAGCAGTTCCCTGTGTCA
TTGTGATAGGAGGTGGTATTTCCGGCCTTGCTGCCGCTCGGACTCTGACTGATGCTTCTTTCA
AGGCAATGATTCTACACCATTATTGGCTACACGGAGTATGCAATGAGAATCCCTTAGCTCC
ATTAATATCCTCTCTGGGGCTTAAATTGTACCGTACTAGTGGTGACAATTCTGTGTTGTATGA
CCATGATTTGGAAAGTTATGCACTTTTTGATATGGATGGTCGTAAAGTTCCACAAGAGATTG
TTGTTGAAGTTGGAGATGTATTCAAGAGAATACTCAAAGAGACTGAGAAAGTACGGGATG
AACACAAGAAGGACATGTCAGTCCTTAAAGCAATATCAATTGTGCTAGATAGGAATCCTGA
GTTAAGACAAGAAGGACTTGCCTATGAAGTGATGCAGTGGTACATATGTAGAATGGAAGC
TTGGTTTGCTGCAGATACAGATATGATATCCTTGAAATGCTGGGATCAGGCAAATCAACTC
CACTTCAACTTTAAACTCTACCTACATCTTTCACTATTGCAAATCAGAATCTCGAAGCTTTTC
AACACTGGTTTGCATGAAGTGACGCTGATTAATTTAGAACAAGTCCTTTTGGGTGGTCATGG
ACTTATGGTGCAGGGTTATGACCCCATAATAAAAGAACTTGCTAAAGATATTGATGTTCGCT
TGAATCATAGTAGGGTTTCTAAAATATCCAGAGGATGTGATAAGGTGGTGGTCAAAGTTGA
GAACGGATTGAGCTTCATTGCTGATGCTGCTATAGTAACTGTACCCCTCGGGGTTCTTAAAG
CCAATTTGATTCAGTTTGAACCAAAGTTGCCAGAATGGAAGGTTGCTGCAATTTCAGATATT
GGTGTTGGTAACGAAAACAAGATTGCCTTACTATTTGACCGAGTCTTTTGGCCAAATGTTGA
GCTGTTAGGCATTGTTGCACGCACTTCTTATTCTTGTGGTTATTTTCTCAATCTTCACAAGGC
AACAGGCCATCCTATTCTTGTCTATATGGCTGCTGGAAGATTTGCTGACGATCTGGAGAAGT
TTTCTGATGAATATGCTGTGAACTTTGTGATGTCGCAGTTGAAGAAAATGTTTCCTGATGCA
ACTGAGCCGGTACAATATCTGGTGTCACATTGGGGAACAGATCCAAATTCCCTTGGCTGTT
ATTCGTATGATCCAGTCGGGATGGCAGGAGATGTGTATGATAAGCTTAGAGAACCTTTGGA
TAATCTTTTCTTTGGAGGGGAAGCAGTTACCGAGGAGCACCAAGGGTCGGTGCACGGAGCT
TACTCTTCTGGAGTCCTGGCTGCCAGAAACTGTGAGAGTCATCTCTTAGAGAGATTAGGTGA
CTTTAGAAAGCTCCAGCTAATCTCCTTTAGTGGTGATGCATTATTAGAACCCATATTTCCTCT
CCAGATATCTAGGATGTGA

>GhPAO8
ATGGATTCTTCTTCACGCTCCGCTGTCATCATCATCGGCGCCGGCATCTCTGGTATATCGGC
GGCGAAGGTTTTGGCTGACAACGGAATTGCGGATTTGCTGATTTTGGAAGCTTCCGGTAGA
ATTGGAGGTAGGATCCGGAAAGAAAGTTTCGGTGGGGTGTCGGTGGAGCTTGGAGCGGGTT
GGATCGCGGGTGTAGGTGGCAAAGCGTCCAATCCCGTTTGGGAGATAGCTTCTAAGTTTGG

CCTCCGAACCTGCTTCTCTGACTACAGTAATGCCCGCTATAACATCTACGATCGGAGTGGG
AAGATCTTTCCGAGTGCAATCGCCGCAGACTCATACAAGAAAGCGGTGGACTCGGCGATA
CAGAAACTAAGGGACCTAGAGTCAAACTCTGTCGAGGATGTCGCCAATGGAGCCGAGTTA
CCTTTAACAGCGAAGACACCGATAGAGCTCGCGATTGACTTTATATTACACGATTTCGAGA
TGGCAGGCAAGTTCCACAACTTATTTTTGTTTTCCTCTGTGGAGCCAATATCAACATACGTA
GATTTTGGGGAAAGAGAATTTTTGGTGGCAGATGAAAGGGGTTATGAGTATTTACTGTATA
AAATGGCAGAGGATTTTCTATTGACGTCGGAGGGAAAAATCCTGGATAATCGCCTCAAACT
CAATAAGGTTGTCAGGGAATTACAGCACTCGAGAAACGGCGTCACGGTGAAAACAGAGGA
TGGTTGTGTTTACGAAGCCAACTACGTCATTTTGTCAGCTAGCATCGGTGTTCTCCAAAGCG
ACCTCATTTGCTTCAAGCCGCCCTTGCCCAGGTGGAAAACGGATGCCATAGAGAAATGTGA
TGTGATGGTATATACCAAGATCTTCCTCAAGTTCCCGTACAAGTTTTGGCCTTGTGGGACAG
ACAAAGAGTTCTTCATCTATGCTCACGAGCGGAGAGGCTATTACACGTTTTGGCAGCATAT
GGAAAATGCGTACCCTGGTTCGAATATTTTGGTGGTAACATTGACCAATGGTGAATCAAAA
CGTGTTGAAGCTCAATCTGATGAAGAGACGTTAAAGGAAGCAATGGGTGTGCTGAGGGAC
ATGTTTGGGCCCGACATACCTACTGCTACAGATATACTTGTTCCCCGATGGTGGAATAATAG
GTTCCAGCGTGGCAGCTACAGCAATTACCCCATAATCTCTAATAACCAAGTTGTTAATGAT
ATTAAGGCCCCAGTTGGACGCATTTTTTTTACTGGTGAACACACAAGTGAAAGATTTAATGG
TTATGTGCATGGAGGATACCTTGCAGGTATTGATACAAGTAAAGCTTTACTGGAAGAAATA
AGAAAAGACGAAAGAGAAAATGAGAGTAAAAGTTTCTTGCTGGAGCCATTAATAGCATTG
TCAGGGTCATTAACTTTGGCACAGTCGGATGCAGTCTCAGGTCTCCAAAAATGTGAGGTTC
CAACGCAATTATATCTTAGCGGCAAGCTTGGCATTCCAGAAGCAATCTTATGA

>GhPAO9
ATGGAGCCTTCTCCTCGGTCGTCCGTTATTATCATCGGCGCCGGCGTCTCTGGTTTATCGGCG
GCGAAGGTTTTGGCTGAGAATGGAATTGGGGATTTGTTGATCTTAGAAGCGTCTGATAGAA
TTGGCGGTAGGATCCGGAAAGAGAAGTTCGGAGACGTCTCGGTGGAGCTGGGAGCGGGTT
GGATTGCCGGTGTAGGTGGCAAAGAGTCCAACCCGGTTTGGGAGATTGCCGCGAAGCTTGG
CCTCCGAACCTGTTTCTCTGACTACAGCAATGCCCGCTATAACATCTACGATCGGAGCGGG
AAGATATTTCCAAGTGGAATCGCCGCCGACTCGTACAAGAAGGCGGTGGACTCAGCGATTC
AGAAACTAAAGGGCCTCGAGTCAAACTATGTGGAAGATGCCACCAATAGAACCGACTTTA
CTTTAACACCGAAGACACCAATAGAGCTCGCAATTGACTTTATATTACACGATTTTGAGATG
GCAGAGGTGGAGCCAATATCAACTTACGTAGATTTTGGGGAAAGAGAGTTTTTGGTGGCAG
ATGAAAGAGGTTATGAATATTTACTGTATAAAATGGCCGAGGAATTTCTATTTACCTTGGAG
GGTAAAATCCTGGACAATCGCCTGAAACTGAACAAGGTTGTTAGGGAATTACAGCACTCG
AGAAACGGCGTCACAGTGAGAACAGAGGATGGTTGCGTTTACGAAGCCGACTACGTGATT
TTGTCTGCTAGCATTGGTGTTCTTCAAAGCGACCTCATTTCCTTCAGGCCACCCTTGCCTAGG
TGGAAAACGGAAGCGATAGAGAAATGTGATGTGATGGTGTATACCAAGATCTTCCTCAAGT
TTCCGTATAAGTTCTGGCCTTGTGGGCCTGGAAAAGAGTTCTTTATCTATGCTCACGAGAGG
AGAGGCTATTACACGTTTTGGCAGCACATGGAGAATGCATACCCTGGTTCAAATATTTTGGT
TGTAACGTTAACGAACGATGAATCGAAGCGTGTGGAATCTCAATCCGATGAAGAGACATTG
AAGGAAGCTATGGTTGTGCTTAGGGACATGTTCGGGTCTGACATACCCGATGCCACTGATA
TACTTGTTCCCCGCTGGTGGAATAACAGGTTCCAGCGTTGCAGCTACAGCAACTACCCTATG
ATATCTAATAACCAAGTCATTAATGATATTAAGGCCCCAGTGGGACGCATTTTCTTTACTGG
TGAACACACGAGTGAAAGATTTAATGGCTATGTACATGGTGGACACCTTGCAGGCATTGAT
ACTAGTAAGGCAGTACTGGAAGAAATGAGAAAAGATGAAAGACAGAACGACAAACAGAA
CCAAAATTTCTTGTTAGAGCCCTTGTTAGCATTGACTCTGACTCAGGCGGATGCAGTCTCAG
GTCTCCACAAATGTGATGTTCCCACACAATTGTATCTCAGCGGCAAGCTTGGCATTCCGGA
AGCGATCTTGTGA

>GhPAO10

ATGGAGTCGTCGGACAAGAGTAATCACCAATTGCGTGGAGGTATTTGCTATCCAAATGGGG
CGAGGAGGCCTGTAAGAACACCTTCAGTAATCGTGATTGGGGCTGGAATGGCTGGAATTGC
AGCTGCACGTGCTCTCCATGAAGCCTCATTTCAGGTTACGGTGTTAGAATCCAGGGACAGA
ATTGGAGGTCGAGTTCATACTGATTACTCATTTGGTTTTCCTGTTGACCTTGGTGCTTCATGG
TTGCATGGAGTTTCGAAAGAAAATCCCTTGGCACCATTGATCAGTAGACTTGGACTACCAC
TTTATCGGACTAGTGGTGATAACTCTGTGCTGTATGACCATGACTTGGAGAGTTATGCACTT
TTTGATATGGATGGTCATCAAGTTCCACAGGAATTGGTCACTAAGGTTGGAGAAACATTTG
AGTGCATTTTGGAAGAGGCAATGAGACAAGAGCACAGTGAAGACATGTCCATAAGTAGTG
CTTTCTCAATTGTTTTCGAAAGAAGACCAGCATTAAGGTTGCAAGGGCTTGCACATAAGGT
ACTTCAGTGGTATGTATGCAGAATGGAAGGTTGGTTTGCTTCGGATGCTGATACCATCTCCC
TTAAAAGCTGGGACCAGGCAGAGCTATTACCTGGTGGTCACGGACTCATGGTCAGGGGCTA
TCTTCCTGTCATAAACACTCTGGCTAAAGGTATTGACATCCGCTTGAGCCACAGGGTTACAA
ACATTGTGAGGCGTTACACTGGAGTGAAGGTTACTGTGGAAGACGGTACTACATTTGCAGC
AGATGCTGTTATCGTTGCTGTTCCTCTAGGTGTACTAAAAGCCAAGACAATCAAGTTCGAAC
CAAGGCTTCCTGAATGGAAGGAAGCAGCAATTGATGAACTTGGAGTGGGAATTGAGAATA
AAATTATATTGCACTTTGACAAGGTGTTTTGGCCTAATGTGGAGTTTTTGGGAGTTGTTGCTG
ACACATCTTATCATTGCAGCTACTTTCTAAACCTTCATAAGGCGACAGGTCATTCTGTCCTC
GTTTATATGCCTGCTGGGCAGCTGGCCAGAGACATTGAGAAAATGTCTGATGAAGCTGCTG
TGGAGTTTGCTTTTATGCAACTCAAGAAGATCCTTCCAGAGGCATGTGCCCCGATTCAGTAT
CTTGTTTCTCGATGGGGCACAGATGTGAACACACTAGGCTCCTATAGCTATGATGCAGTAG
GCATGTCCCATGACCTGTATGAGAGGCTAAGGGTGCCAGTGGATAACATATTCTTTGCGGG
GGAGGCAACCAGTATGAGCTACCCAGGGTCCATTCATGGTGCATTTTCAACTGGGCAGATG
GCTGCTGAGGACTGTAGGATGCGTGTACTGGAGCGATATGGAGAGTTGAACTTGCTCCAAC
CAGTTATGGGCGAGGAAGCAGGGTTGTGCGTCCCGCTTTTAATAACGCGCTTGTAA

>GhPAO11
ATGGAGACGCCAGTTTCAGAAGGGTTGGTTTCAAAGAGGTCGTTGAGGAAGAAATCCGCA
GTGAAGAATTATGATGAGAATTTAATGGATGAGTTCATAGAGAAGCATATAGGTGGTTCGT
TTAGGAAGATTAGAACAAAGGAGGAGTTGGAGAAAGAGACTGAAACTGAGGCAATGATA
GCATTATCTTTGGGTTTCCCTATTGATGCACTGATTGAGGATGAAATTAAAGCAGGAGTGGT
AAGAGATATAGGTGGAAAAGAGCAGAATGATTACATTGTTCTCAGGAATCATATCCTCTCT
CGATGGAGGAGTAATGTACGGATATGGTTATCTAAAGGACATATAAGAGAAACCGTGAGT
AATGAATATGAACATCTGTTGTCTGCTGCTTATGATTTTCTTCTGTATAATGGGTATATTAAT
TTTGGAGTTTCACCATCCTTTTCCTCTTACATCCCAGCGGAGGCAACTGAGGGTTCTGTGAT
AATAGTTGGAGCTGGACTTGCTGGCTTGGCAGCAGCAAGGCAACTTATATCTTTTGGTTTCA
AGGTTGTTGTTATAGAAGGGAGGAATCGACCTGGGGGAAGAGTTTATACTCAACTGATGGG
TAAGAAGGATAAGCGTGGTGCTGTGGATCTTGGTGGTAGTGTAATCACCGGCATCCATGCC
AATCCTCTTGGAGTTCTGGCCCGGCAACTTTCTATTCCACTTCATAAGGTCCGAGATAATTG
CCCTTTATATAAACCTGATGGGGTGCCTGTCAATAAGGTAATCGATTCCAAGACTGAAATG
ATCTTTAACAAGTTGCTTGACAAAGTCAATGAACTGAGAAAAATAATGGGTGGATTTGCTA
ATTATATTTCTCTTGGATCAGTTCTGGAAAAGCTAAGACAGTTGTATGGTGTGGCTAGAAGC
CCAGAGGAGAGACAACTTTTGGAATGGCATCTTGCTAATTTGGAATATGCAAATGCAGGAT
GTCTTTCTGACTTGTCGGCTGCCTACTGGGACCAGGATGATCCTTATGAGATGGGTGGAGAC
CATTGTTTTCTTGCTGGAGGGAACTGGAGATTGATAAAAGCATTATGTGATGGAGTTCCCAT
AATCTATGGGAAAACAGTTGATGCTATTAGATATGGTGTTGAAGGAGTTGAGGTTGTTACC
GGTAAGCAAGCATTCCAAGCAGATATGGTTCTTTGTACTGTGCCTCTTGGAGTCTTGAAGAG
AAGGACCATTAGATTTGAGCCAGAGTTACCTCAAAGAAAGCTAGCTGCAATTGACAGACT
AGGTTTTGGGCTCCTGAATAAAGTTGCCATGATTTTCTCTCATGTTTTTTGGGGAGAAGAGTT
GGACACATTTGGATGTCTCAATGATACCAGTGATAACCGTGGGGAGTTCTTTTTATTCTACA
GCTACCACACTGTTTCCGGGGGTCCAGTGTTGATTGCGCTGGTGGCTGGTAAAGCTGCACA
AACATTTGAGCGCACAGATCCTTCACTCTTGCTCCATCGTGTTCTAAGCAAACTTAGAGGTA

TATATGGTCCAAAAGGTGTAGATGTACCCGACCCTATACAGACAATTTGTACAAGATGGGG
AAATGATCCCTTTTCATATGGTTCATACTCTCATGTTAGGGTACAGTCATCCGGCAGGGATT
ATGATATACTTGCAGAAAGTATAGGCAATAGGTTGTTTTTTGCTGGTGAAGCCACAACTCG
GCAATATCCAGCCACCATGCATGGTGCCTATTTGAGTGGGTTAAGGGAAGCTTCGCGTATTC
TCCGTGCCACAAGAGGTCGCCAAAACTACTTTAGGAGGTCTGTGCAGCGGAATGTTGGACC
AAGCAGTGATCAATTGGGTGATCTGTTCAAGATGCCTGATCTAGTATTTGGGAAGTTCTCTT
TTGTGTTCAATCCATTAACAGAGGACCCTAAATCATTGGGGCTTTTGAGAATTACTTTTGAT
AATTGCACAGAAGATACGAGGAAGGTGCTGGAAAAAAGCTGTGACCCCCAATCGAATCAA
TCATTGCAGCTGTACACAGCATTGTCCCGTGAACAGGCACATGAGCTACAGATGGTAACTG
GAGAAGATGAAAGTAAGTTGGTTTATTTGATAAATAATATTGGATTAAAGCTTATGGGAGC
TAATGCTCTTGGAATCACATATAACTCCTTGGTTACTAGCATATCTAGTGCAAGAAAAGGTA
GGAGCAGGTACTGTATATCTGCGCCACTGCTAAATACAGTTTAG

>GhPAO12
ATGGATGGTGGTGGAGGCTCTAAGAAAAGATTAAAGGTCACAGCGGTGGAGGTTGGTGTT
GATTCAGATGATGATGAGCCCATTTTGTCTTTGTTGAAGTTAAGGAGACCTAAGAATCCTAA
AAAGGATAAGGCTGGATTGGAAGGCAGTGCGGGGAAGTGCCAAAAGGTTGAAGTTAAGG
CAGGTAAAACTGTGGACAAAGATGAGGAGGATTTTGGGGGAATGAATGATACATTGGCCA
GCTTCAGAAAGAAGTTAAAGGATCCTAAAAAAGATATTGATCCAGGGGCAATGAGGGTAA
GGAGTAACTCTTTGAATAAGTCTGTGGAGGGTGGTGGAATTTTGGATGGGAAATCTGTGTC
GAACACTGATGTGAAAGGTCAGGATATTGGTGAAGACAGGTCTGATGTGGCTACTGATAAA
GTTGTTGGAAAAAAGCGTACAGGGAAAGTAAGGAGAACCAAGTCAGATTCAAAAGCCAA
GCCCAGTGAGGTTGATGATGAATCCAGAGCTAAACTTGAGGAAGATCAGAATGAGGGAGG
TTTGTCGCCTGGGGAAGGTTTGTATCAGTGTTCTCACAAGGCACAATCTGGTTCAGTAGGGA
AATCTTGCCCAATTTTTTGTTTGAAACATAATTGCGAGGCTGCTCATCATGCTTCTGATTCAA
AGAATCCTAGCAGAAATTGTGGTGATAGTTCTCATTCAGTTTCTAGTTCAAGCTTCTCACAT
TCATCCTCCAAAGAATGCAACACAGCTGAGAATCAAGGATTTGACCACAGTTTGTATCAAC
AAGAAAGCATTTTGGAACCAGGTGACTTAACTGTTCAAAAGGATCCTACAGAATATCCATG
TAGGTCATCTAAAGTTTGTGACAAGGAAAGATATTGTCATTCCAACATTGAGCTCAGGGAC
AATTTCTCAGCAATTGACCTGAGGAGTAAACCAGGAACTGAAAGTTCACAACAAAATAAA
CTTAATCTGTCGCTGTCTGTTGTTGATTCACTGAAGATGGAAGAAACTTGCACTGACGTTCC
AAATTCTTGCGCTGAGGAATACTCTTTAGAAACTTCCATTCATCCCAATGAACTTGTTGCCT
CCATTCAGAGGTGCAACTCTGCTCTGCATCAACCTTCTGAAGATGCAAGCCCTGGTGCTTGT
GGTCCCATCCATGATACTGTTTTCATCAGCAAAAAGGCCAATGTTGACTCTCCCATTTCAAC
ACCTGATGAAAATGAAAGTTGCCATGAAGATGCAGTCTCTCTCCCTAGTTCTGAACTAAAA
AACAGCAAGTCATCAGCTTTCCAGCGAGGTGGGCGCAATATTAAAAAGCGTAGACATGGA
GATATGGCTTATGAAGGGGATGCTGATTGGGAAAATTTGCTAACTGAGCAAGGTTTTTTGG
AAATCAACAATTTGCAGACAGTGATCATTCCTTTAGAGCAAGAGAGAAGTTTGATGAGGCA
GCAATATCATCTGGACTGAAAGCTTGTGCTGTGGGGCCAGTTGAGAAGATCAAATTTAAGG
AGGTGTTGAAGTGTAGAGGTGGTCTACAGGAATACTTGGAATGCAGGAATCATATCTTAGG
TCTTTGGAGTAAAGATGTTAACCGCATTTTGCCTCTTGTTGACTGCGGTGTTAGTGACACTCC
TTCAGAGGGTGAACCATCCCGAGCTTCCCTAATTAGGGAGATATATGCATTTCTTGATCAGG
GTGGTTACATAAACTTTGGAATTTCTTCAAAGAAAGAGAAGGCTGAGCTTAGTGTTAAGGA
CAACTACAAACTTCTCGAGGGAAGAAAAAGTGATGGTAATTCTGTTGCCTCTGTTGCTGATT
CAGAGGATGGAGTTGCCTTTATCCTTGGTCAGGTCAAGAATTCTAAAGCTTCTATGGATGCA
AAGACTGGTCTTAGAGTTGTTGATGAAAACCAGGCATCTGAAGCGACAATAGCAATAGCTG
AAGTATTGGTTGATTCAATCACACCAGAATTACCTTATAAATGCCAGCAAAATGGCAGCTT
TAGTGCAAAATTGAACACTGGATTGATTAGTTCGCAGGTTTCTAGTACTGATCTATCTTGTG
ATGCAACTGATGTTGGAGTAGCCCCTGTAGTAACTCCAGAAGAAAGGAATGACTCACAGT
ATGTTCAATCTGCAACTTATGATAAACCTGACGGGAATCATCAGCTGCTGAATGATTCAGA
GGTCAGAAAGAACATCATAGTTATTGGAGCTGGTCCTGCTGGATTGACTGCTGCACGCCAC

TTGAAACGTCAGGGATTTTCTGTAGTTGTACTTGAGGCTAGGGATAGGATAGGAGGTCGTGT
TTATACTGATTGCTCCTCTCTTTCAGTACCTGTGGATCTTGGGGCTAGCATTATTACTGGAGT
TGAGGCTGATGTGTCAACTAATAGAAGACCAGATCCATCCTCATTGATTTGTGCACAGTTGG
GGCTAGAGTTAACTGTGTTGAATAGTTCCTGTCCTCTTTATGACATTGTATCTGGTCAAAAG
GTTCCTGCTGACCTGGATGATGCTCTTGAAGCTGAATACAATAGTCTTCTTGACGATATGGT
GTTTCTTGTTGCTCAAAAAGGTAAAAAAGCAATGACAATGTCTCTTGAGGATGGTTTAGAA
TATGCCCTAAAAAGGCATCGGATGGAAGAAATAGGAGCAGATATTGAAGAAACAGAATC
ACATTCTTCAGTGGATGCTTTCTATGACTCAAAAGCTAGCAATATCTTTGGATTTCCTCGAA
AAAAATGTTCCNATGATGCTCTGGAAGCTGAATACAATAAGGAGATTTTGAGTCCTCTTGA
GAGAAGGGTCATGAATTGGCACTATGCTCACTTGGAGTATGGCTGTGCTGCTTCGCTTAAGG
AAGTTTCTCTTCCCAACTGGAATCAGGATGATGTTTATGGCGGCTTTGGAGGAGCCCATTGT
ATGATTAAAGGGGGTTACAGTACGGTGGTTGAGTCTCTTGGAGAAGGGCTTCTGATCCACTT
GAACCACGTAGTCACAAATATTTCATACAGCCCAAAGGGCCCGGGGGTTGATAATAGTCAT
CATAGGCAGGTCAAAGTTTCCACATCAAATGGCAGTGAGTTTTCAGGAGATGCTGTGCTTA
TCACTGTGCCACTTGGTTGCTTGAAAGCAGGAGCCATAAAGTTTTCTCCTCCGTTGCCCCAA
TGGAAACATTCTTCCATACAGCAGCTTGGTTTTGGAGTACTTAATAAAGTCGTCTTGGAATT
CCCAGAAGTTTTTTGGGATGATACTGTGGATTACTTTGGAGTGACTGCTGAGGAAACAGAT
AGTAGAGGCCATTGTTTTATGTTTTGGAATGTCCGAAAAACTGTTGGGGCTCCTGTTCTTATA
GCCTTAGTGGCTGGTAAGGCAGCTATTGATGGTCAAAATATGAGCGCATCAGATCATGTAA
ACCATGCTGTAATTATTCTCCGTAAACTTTTTGGAGAGGCTTCAGTTCCTGATCCTGTTGCCT
CAGTTGTTACTGATTGGGGAAGGGATCCTTTTAGTTATGGCGCTTACTCCTATGTTGCCATAG
GAGCATCTGGAGAAGACTATGATATGTTGGCCAGGCCTGTTGAGAACTGCTTGTTTTTTGCT
GGAGAAGCTACCTGCAAGGAGCATCCTGACACAGTTGGTGGTGCAATGTTGAGTGGGCTTC
GGGAGGCTGTGCGATTAATTGACATATTTACCACTGGAAATGATTATACAGCCGAAGTAGA
GGCAATGGAGGCTGCCCAGAGACATTCAGAATCAGGAAGGGATGAAGTTAGGGACATAAT
TAAGAGACTTGAAGCAGTTGAACTTTCTAATGTCTTGTACAAAAACTCTTTGGATCGTGCTC
GGATTTTAAGCAGGGAAGCTTTACTGCGGGACATGTTCTTTAATGTGAAAACCACTGCAGG
ACGATTGCATCTAGCCAAGAAGTTGTTAGGTCTCCCAGTTGAATCCTTGAAATCCTTTGCTG
GGACAAAGGAAGGGCTTAGCACACTCAACTCATGGATGCTGGACTCAATGGGGAAAGATG
GGACGCAACTGTTGCGCCATTGTGTTCGTCTTCTTGTGCTTGTTTCAACTGATCTACTTGCAG
TTCGTTCATCAGGCATAGGGAAAACTGTGAAGGAAAAAATTTGTGTGCATACAAGTCGTGA
TATACGTGCTATTGCAAGCCAGCTGGTTAATGTTTGGCTTGAAGTCTTCCGTAAGGCAAAAG
CTTCTTCAAAGAGAAAATCCCTTAAAGATCCAGCTTCAGGAAAGCCACCTCTGCACTCACA
GCATGGTGCTTTTGAGAGTAAAGAAAGCTTGCAGGATCCATTTTCTGCTGGAAAGCAGTAT
CCTTTAAACATAAAGGAGAATGGCAAATCACTTGATATTGAGGTGGAAGCTGTCAACCAA
GGAATGTCAGAGGAAGAGCAGGCTGCCTTTGCTGCTGAAGCAGCTGCCCGAGCTGCAGCA
AAAGCAGCTGCAGAGGCACTTGCATCCACAGAAGCTAACTGCAACAAATTACTGCAGCTT
CCTAAGATTCCTTCTTTTCACAAATTTGCCAGAAGGGAGCAATACGCACAAATGGATGAAG
GGAAATGGCCTGGTAGTGTTTTTGGAAGACAAGATTGTATATCAGAAATTGACTCTAGGAA
CTGCAGAGTCAGGGACTGGTCTGTTGATTTCTCTGCTGCTTGTGTTAACCTTGACAATTCCGG
AATGTCAGTAGATAACCTGTCTCAGAGGAGCCACTTGAAACTCAGAGAACACTCTGGAGA
AAGTTTGGCTGTGGATAGCAGTATCTTCATGAAAGCATGGGTTGATACTGCTGGTAATGGG
GGGATCAAGGATTATCATGCCATTGAGAGATGGCAGTCTCAAGCAGCTGCTGCTGATCCAG
ATTTCTTCCATCCTACAAATTTCAAGGATGAGGAAGATTCAAATACTAGTTCAAGGCAACC
AACCTGGAAGAATGATGGGCGAGCAAATGAGAGCTCCGTCTCCCAAGTTTCTGTAAACAA
GGAGCGATTTGAAAATCATCCCCATGGAGCTGATTGTATTAAACAGGCTGTCGTTGATTATG
TTGCATCATTGCTAATGCCCCTTTATAAGGCAAGAAAAATTGATAAGGAGGGATACAAATC
GATAATGAAAAAAACTGCAACAAAGGTAATGGAGCAGGCAACAGATGCAGAGAAAAACA
TGGCTGTTTTTGAATTTCTTGATTTCAAGCGCAAAAACAAGATTCGTCCCTTTGTAGACAAA
TTGATTGAGAGGCACATGGCAATGAAGCCAACCATGAATCTATGA

>GhPAO13
ATGGAAAGTGATGACGGTTCAAACTCCAAGGCAGGTCCACAATTGCAATTTGATTTGGGGA
ATAATGTTGAGCTAGGGCTAGAAAAGGTTACATTACAGAATTCTTTGAAACTTAGGAACAA
GAAAATGGATGGTGGTGGGGGCTCTAAGAAAAGACTAAAAGTAACAGCGGTGGAGGTTGA
TGTTGATTCAGATGATGATGAGCCCATTTTGTCCTTGTTGAAGTTAAGAAAATCTAAGAATC
CTAAAAAGGATAAGGCTGGATTGGAAGGCAGCGCTGGGAAGTGCAAGAAGGTTGAAGTTA
AAGCAGTTAAAACTGAGGGCAAGAATGAGGAGGATTTGGGGGGAATGAATGATACGTTGG
CCAGCTTTAGAAAGAAGCTAAAGGATCCCAAGAAAGATGTTGATCTAGGAGCAAAGAGGG
AAAGGGATTATTCTTTGAATAAGTCTGTGGAGGGTGGTGGAGTTTTGGATGGGAAATCTGTG
TTGAACACTGGTGTGAAAGGTCAGGATATTGGTGAAGACAGGTCTGATGTGGTTACTGATA
CAGTTGTCGAAAGAAAGCATACAGGGAAAGTAAGGAGAGCCAAGTTTGATTCAAAATCCA
AGCTCATCGAGGTTGATGATGAATCCAGAGCTAAGCTAGAGGAAGATCAGAATGAGGGAG
GTTTGTCGCCTGAGAGTGGTTTGAATCAACATTCTCACGAGGCACAATCTGATTCAGTGAGG
AAATCTTGCCCAATTTCGAGTTTGAAACGTAATTGCAAGGGTTCCTATCATGCTTCTGCTTC
AAAGAATCCTAGCAGAAATTATGGTGATAGGTCTCATTCAGATTCTAGTTCAAGCTTCTCAC
ATTCATCCTCCAAAGAATGCAACACAGCTGAGAATCAAGGATTTGACCATAGTGTGTGTCA
ACAAGAAAGCATTTTGGAACCAGTTGACTTAAATGTTGAAAAGGGTCCTACAGAGGACCC
ATGTAGGTCACCTAAAGTTTGTGACAAAGAAAAATATGGGCATTCCAACATTGAGCTCAGG
GACAATTGCTCAGCAGTTGACCAGAGGAATAAGCCAGAAAGTGAAGGTTCACCACAAACT
AAACATAATCTGTTACCGTCTGTTGTTGATTCACTGAAGATGGAAGAGACTTGCACTGATGT
TCCAAATGCTTGCGCTGAGGAAAACTCTTTAGAAAACTCTGTTCATCCCAATGAATTTGTTG
CCTCCATTCAGAGGTGCAACTCTGCTCTCCGTCAACCTTCTGAAGATGCATGCCATGGTGCT
TGTGGTCCCACCCATGATACCCTTTTCATCAGCAAAGAGGCCAATGTTGATTCTCCCACATC
AACACCTGATGAAAATGAAAGTTTTCATGAAGATGCAGTCTCTCTCCCCAGTTCTGAAATC
AAAGACAGTATGTCATCAGCTGTCCAGCGAGGTGGGCGCAGTATTAAAAAGCGTAGACAT
GGAGATATGGCTTATGAAGGGGATGCTGATTGGGAGAATTTGCTAAATGAGCAAGGGTTTT
TTGGAAATCAACAGTTTGCAGACAGTGATCGTTCCTTTAGAGCAAAAGAGAAGTTTGATGA
GGCAGCAGTATCATCTGGACTGAAAGCTCGTGCTGTGGGACCAGTTGAGAAGATCAAATTT
AAGGAGGTCTTGAAGGGTAGAGGTGGGCTACAGGAATACTTGGAATGCAGGAATCATATC
TTAGGTCTTTGGAGTAAAGATGTTAACCGCATTTTGCCTCTTGCTGAGTGCGGTGTTAGTGA
CACTCCTTCAGAGGGTGAACCACCCCGAGCTTCCCTAATCAGGGAGATATATGCATTTCTTG
ATCAGGGTGGTTACATAAACTTTGGAATTGCTTCAAAGAAAGAAAAAGCTGAGCTTAGGGT
TAAGGATAACCACAAGCTTCTCAAGGAAAGAAAAAATTATGGCAATTCAGTGGCCTCTGTT
GCTGATTCAGAGGATGGAGTTGCCTTCATCCTTGGCCAGGTCAAGAATTCTGAAGCCTCTTT
GGATGCAAAGGTCGGTGTTAGAGTTGATGATGAAAACCAGGCATCTGAAGCCACAATACC
TGAAGTGTTGGTTGATTCGATCACATCAGAATTACCTTGCGGAAAAGAACAAAAGGAACA
CCCAAGTGATAATTGCCAGCAAAATGGCAGCATCAGTGCAAAACTAAACCCTTTATTGATT
AGTTCGCAGGTTCCAAGTGCAGATCTATCTTGTGATGCTATTGACATGGGAATAGCCCCTGT
AATAACTCCAGAAGAAAGAAATGACTCACATTATGTTCAGTCTGCAACTTATGATAAACCT
GATGGGAATCATCAACTGCAGGGTGATGCAGAGGTCAGAAAGAACATCATAATTGTTGGA
GCTGGTCCTGCTGGATTGACTGCTGCACGCCACTTGAAACGTCAGGGATTTTCTGTAGTTGT
ACTTGAGGCTAGGGATAGGATAGGAGGTCGTGTTTATACTGATTGCTTCTCTCTTTCAGTAC
CCGTGGACCTTGGGGCTAGCATTATTACTGGAGTTGAGGCCGATGTGTCAACTAATAGAAG
ACCAGATCCATCCTCATTGATTTGTGCACAGTTGGGGCTAGAGTTGACCGTGTTGAATAGTT
CCTGTCCTCTTTATGACATTGTATCTGGTCAAAAGGTTCCTGCTGATCTGGATGATGCTCTGG
AAGCTGAATACAATAGTCTTCTTGATGATATGGTGTTTCTTGTTGCTCAAAAAGGTCAAAAA
GCAATGACAATTTCTCTTGAGGATGGTTTAGAATATGCCCTAAAAACGCATCGGATGGAAG
AAATAGGAGCTGATATTGAAGAAATAGAATCACATTCTTCAGTGGAAGCTGTCTATGACTT
GAAAGCAAGCAATGGAAAAAAATGTTCTGAAGGGGAGATTTTGAGTCCTCTTGAGAGAAG
GGTTATGAATTGGCACTATGCCCACTTGGAGTATGGCTGTGCTGCTCCGCTTAAGGAAGTGT
CTCTTCCCAATTGGAATCAAGATGATGTTTATGGCGGCTTTGGAGGAGCCCATTGTATGATT

AAAGGAGGTTACAGTAAGGTGGTTGAGTCTCTTGGAGAAGGACTTCTGATCCACTTGAACC
ATGTAGTCTCAAATATTTCATACGGCCCAAAGGACCCTGGGATTGATAATAGTCATCATAG
GCAGGTCAAAGTTTCCACATCAAATGGCAGTGAGTTTTCAGGAGATGCTGTGCTGATCACT
GTGCCACTTGGTTGCTTGAAAGCAGGAGCCATAAAGTTTTCTCCTCCATTGCCCCAATGGAA
ACATTCTTCCATACAGCAACTTGGTTTTGGAGTACTTAATAAAGTTGTTTTGGAATTTCCAGA
AGTTTTTTGGGATGATACTGTGGATTACTTTGGAGTGACTGCTGAGGAAACAGATAGTAGA
GGCCATTGCTTTATGTTTTGGAATGTCCGAAAAACTGTTGGGGCTCCTGTTCTTATAGCCTTA
GTGGCTGGTAAGGCAGCTATTGATGGTCAAACTATGAGCTCATCAGATCATGTAAACCATG
CTGTACTTATTCTCCGAAAACTTTTTGGTGAGGCTTCAGTTCCTGATCCTGTTGCCTCAGTTG
TGACTGATTGGGGGAGGGATCCTTTCAGTTACGGTGCTTACTCCTATGTTGCCATTGGAGCA
TCTGGAGAAGACTATGATATGTTGGGCAGGCCTGTTGAGAACTGCTTGTTTTTCGCTGGAGA
AGCTACTTGCAAGGAGCATCCTGACACAGTTGGTGGTGCAATGTTGAGTGGACTTCGGGAG
GCTGTGCGATTAATTGACATATTTACCACTGGAAATGATTATACAGCTGAAGTAGAGGCAA
TGGAGGGTGCACAGAGACGATCAGAATCAGGAAGGGATGAAGTTAGGGACATAATTAAG
AGACTTGAAGCAGTTGAACTTTCTAATGTCTTGTACAAAAACTCTTTGGATCGTGCTTGGGT
TTTGAGCAGGGAAGCTTTACTACGGGACATGTTCTTTAATGTGAAAACCACTTCAGGACGA
TTGCATCTAGCCAAAAAGTTGTTGGGTCTCCCAGTTGAATCCTTGAAATCCTTTGCTGGGAC
AAAGGAAGGGCTTAGCACACTCAACTCATGGATGCTGGATTCGATGGGGAAAGATGGGAC
TCAGCTGTTGCGCCATTGTGTTCGTCTTCTTGTGCTTGTTTCAACTGATCTACTTGCAGTTCGT
TCATCAGTGGCTACAATGACTTTTTTATTAATAATTTTTCNCCTGAAACTTAGAGAACACTCT
GGAGAAAGTTTGGCTGTGGACAGTAGTATCTTCACAAAAGCATGGGTTGATAATGCTGGTA
GTGAGGGGATTAAGGATTGTCATGCCATTGAGAGATGGCAGTCTCAAGCAGCTGCTGCTGA
TCCAGATTTCTTCCATCCTACAAATTTCAAGGATGAGGAAGATTCAAATGCTAGTTCAAGGC
AAACAACCTGGAAGCATGATGGACGAGCAAATGAGAGCTCCATCTCCCAAGTTTCTGTTAA
CAAGGAGCGATTTGAAAATCATCCCCATGGTACTGATCGTATTAAGCAGGCTGTCGTTGATT
ATGTTGCATCATTGCTAATGCCCCTTTATAAGGCAAGAAAAATTGATAAGGAGGGATACAA
ATCGATAATGAAGAAAACTGCGACAAAGGTAATGGAGCAGGCAACCGACGCAGAGAAAA
ACATGGCTGTTTCTGAATTTCTTGATTTCAAGCGCAAAAATAAGATTCGCCCCTTTGTAGAC
AAATTGATTGAGAGGCACATGGCAATGAAGCCAATCATGAAACCATGA

>GhPAO14
ATGGCAAAGAAGCCAAGAATTGTGATAATTGGAGCTGGGATGGCCGGTCTTACTGCAGCTA
ACAAGCTCTATACTTCCACTGGCTCTGACCATTTGTTTGAGCTTGTTGTTGTTGAAGGTGGTG
ATAGAATTGGTGGCAGAATCAACACTTCGGAGTTTTGTGGTGACAGAATTGAGATGGGTGC
TACTTGGATCCATGGTATAGGAGGCAGCCCGGTACATCAAATTGCTCGGGAAATCCATGCA
CTTGAGTCTGATAAGCCATGGGAGTGTATGGATGGGTTCTCGGGTGAGCCAAAGACTATTG
CTGAAGGTGGGTTCGAGCTAAATGCCTCCATCGTTGACCCCATATCCACCCTTTTCAAAAAC
CTGATGGATTTCGCTCAAGGGAAGCTGACTGAATACAGTGCAGGCAGCGGAGGAGATGCT
TGTTACTACAATTTTGCAGCTAAAGCAGCCTTGAAAGATTGTACGAGCAATGGTGGCTTTGG
TAACCAGAGTGTCGGTGCGTTTCTTAGACGAGGCCTTGGTGCTTACTGGGATTCTTGCAAGG
ACCGTGAGGAGCTGAACGGATATGGTAAATGGAGCAGAAAATTGCTTGAAGAAGCCGTTT
TTGCCATGCATGAAAACACCCAGAGAACTTATACTTCTGCCGGTGATCTGTTCAATCTAGAT
TACGAGGCAGAAAGCGAGTACCGTATGTTTCCTGGTGAAGAAATCACCATTTCTAAAGGCT
ATTTGAGCATAATTGAACATCTTGCATCTGTTCTTCCTCCTGGCGTAATCCAATTAGGCCGC
AAAGTCACAAGAATCGAATGGCAACCTGAGGGTCATAAATCTATACAAGTTCCAAACGGC
TATGATTCCAGACCAGTGAAGATTGAGTTTTGTGATGGATCTTTTATGTTAGCAGATCATGT
GATAGTCACAGTTTCATTAGGGGTCTTAAAATCTGGAACTGGTCAAGATTCAGGCATGTTCA
ATCCTCCCCTTCCTCCTTTCAAGACAGAGGCTATATCAAGACTTGGATATGGTGTTGTTAAC
AAGCTGTTCCTTCAATGGAGTCCAAATGGTAATCGACCGGCCAATGATAAAGAGAAGTTTC
CTTCCTTGCAAATTGTTTTCCATCCCCCAGAATCCGAGTTAAGGCATGAAAAGATCCCAGG
GTGGATGAGGAGGACAGCTTCACTGTCTCCTATTTATAACAATTCAAGCGTCCTCCTATCCT

GGTTTGCAGGTAAAGAAGCACTTGAGCTTGAAACACTTAGCGATGAAGAGATTATAAATG
GAGTTTCAGCAACAGTATCTGGTTTATTACCAGTATCAAAACACAAGAAGGAAGACCAGTA
TAATTCCCCTGAATTCTGCAATGGGAATGTGGAGAGCTGTGATGACAATGGAGTGAGATTT
GGTAAGGTTTTGAAGAGCAAATGGGGCAGTGATCCATTATTCTTGGGATCTTACAGCTACGT
GGCTGTTGGATCAAGCGGTGCTGATTTAGACACAATGGCTGAACCCTTACCAAAGCTTGGG
AGCACTGACTCAGACCACCATCCACTTCAAATATTGTTTGCTGGGGAGGCTACACACAGAA
CCCACTATTCTACAACCCATGGAGCTTATTTCAGTGGTCTTAGGGAAGCCAATAGGCTTCTC
AAACATTATCGTTGTGTTGGGGTTTAG

>GhPAO15
ATGGAGCCTCCCCAAGATACCTCCGAGAACCCTAACGATGTCCTTTCCGACGATGACTCTT
CACCGGAAAACACCAATCCCGACGATCAAGAAATCCCCAGTACGACACTCGACCCACCTA
TTTCCGATACCCAAGATGAATCCTCCGATCCCGTCCCCGACGAGCAACCCCAAAACACTAA
TTCGAACCCCGCGGAGCCTGGTCCACCTGCACGCAAGCGCCGCCGCAGAAAGCGTTTCTTT
ACTGAACTTATCGCCAATCCATCCTTCTCCAAGAACCGTCGCCCTAGAATATCGGGCCTAG
CTAGAGAAATGGACACCGAAGCTTTAATCGCGATTTCTGTTGGTTTCCCTGTTGATTCTCTTA
CCGAAGAAGAAATCGAAGCCAACGTGGTGTCCAGAATCGGAGGCCAAGAGCAAGCCAAC
TACATCGTTGTAAGAAATCACATTCTGGCTCGCTGGAGATCCAATGTATCCGTCTGGCTGAC
GCGCGAGCACGCCCTCGAATCAATCCGAGCTGAGCACAAGAACCTAGTGAACGCAGCATA
CAATTTCCTTCTCGAACACGGTTACATTAATTTCGGTTTAGCCCCGGCTGTTAAAGAAGCGA
AATTGAAGTCTTTTGATGGTGTAGAAAGAGCCAATGTGGTGATTGTGGGTGCGGGTCTTTCC
GGTTTGGTCGCGGCGAGGCAATTAGTTTCCATGGGGTTTAAAGTTGTCATCTTAGAAGGTAG
GACGCGCCCTGGAGGGCGCGTGAAGACAAGGAAGATGAAAGGTGATGGGGTGGTGGCTG
CAGCGGATCTTGGTGGGAGTGTTCTTACGGGAATAAATGGAAATCCACTTGGGGTTCTTGC
AAGGCAAATGGGATTACCGCTTCATAAGGTGCGAGATATTTGTCCTTTGTATTTGCCAGATG
GAAAGGCCGTAGATGCTGATGTTGATTCTAGGATAGAAGTTTCATTTAATAAGCTATTGGAT
AGGGTTTGTAAGCTTAGGCATTCTATGATTGAGGAAGTTAAATCAGTTGATGTTCCATTAGG
GACAGCATTAGAAGCCTTTAGGAGTGTTTACAAGGTTGCTGAGGATTCACAGGAGAGCATG
TTGTTGAATTGGCATCTTGCTAATCTTGAATATGCTAATGCTTCCTTGATGGCTAATTTGTCT
ATGGCCTATTGGGATCAAGATGATCCATATGAGATGGGCGGCGATCATTGTTTCATACCTG
GTGGCAATGAGAGGTTTGTTCGAGCACTTGCGGAGGACCTTCCCATTTTCTATGGGAGGACT
GTGCAGAGTATCAGGTATGGTATTGATGGTGTTAGGGTTTACGCCGGTGGGCAGGAGTTTTG
TGGGGATATGGCTCTTTGCACTGTTCCATTAGGAGTTCTCAAGAAGGGATCGATAGAATTTG
TTCCTGAGCTTCCGCAAAGAAAGAAGGATGCCATTCAGAGACTGGGATTTGGGTTGCTGAA
TAAGGTTGCTATGTTGTTTTCATACAATTTTTGGGGCGGAGAGATTGATACTTTTGGCCACCT
GACAGAAGACCCAAGTATGAGAGGCGAGTTCTTTTTGTTTTATAGCTATTCTTCTGTGTCAG
GTGGTCCACTCCTTGTTGCTCTAGTTGCCGGAGATGCAGCAATCAAGTTTGAACTGATGTCT
CCTGTTGAGTCTGTGAAAAGGGTTTTAAACATATTGCGAGGCATTTTTCATCCAAAAGGGAT
TGTTGTTCCTGATCCTGTCCAGGCTGTTTGTACCCGGTGGGGAAAGGATCGCTTCACGTATG
GATCCTACTCTCATGTTGCTATTGGTTCATCCGGGGATGATTATGATATTCTAGCTGAGAGTG
TTGGAGATGGGAGAGTCTTCTTTGCTGGTGAGGCAACTAATAAGCAGTATCCTGCCACAAT
GCATGGAGCCTTTTTAAGTGGCATGAGAGAGGCCGCTAACATGCTTAGAGTGGCCAGGAG
GAGGTCATTGGTTCTATCTGACAAAGTTAATAACGACTTGGAGAAATGTGATACTTTGGAT
AAGTTGTTTGAGAACCCTGACCTGACATTCGGGAGCTTCTCAGCTTTGTTTGATCCCCATTCT
AATGATGTTGGATCGCATGCATTAATAAGGGTCAAATTTCATGGGGATAAATTAACCTCGA
GTCACTTGTGTCTTTATGGCTTGATAACGAAGAAGCAAGCCATTCAGTTAAGTGAAATGAA
TGGAGATGGGAACAGGATGAATTCGTTGTATCGTGACTTTGGGGTGAAGTTGGTTGGTGGT
AAAGGGTTATCAAATGTTGCGGAGTTGCTGATATCACGCATCAAAGCAGCTAAACCAACCT
AA

>GhPAO16

ATGAACTCACCAAATGAAACCCCTGATCAATTCTCCCAATTCCCCCTTCCCCATTTCACTCT
CACTCCACCTTTACCAAACCCTAACCCTAATTTCCCTCCAACCCCAAACTCAACTCCAACAC
TAACCCCAGTCCTCGATTCTAACACCAATGCTACACCTTCTCTCGATGATCAACTTCTACCC
TTCCCAGTTCCCAAAAAACGACGACGCGGCAGGCCCCGACGCACTGCCTCAACGTCATCGT
TTCAACTCCTTACCTTCCCCAACGATTCATTCAACCCCAATGTTCCATACTCTGACCCTAACC
CTTATTCGATTCCCTCATCAGTAGCGGCGTCGACACAAACTTCACAACCCAAAATTGCTGA
CGAGATCATTGTTATCAATAAAGAATCGACGGCTGAGGCTCTCACCGCTCTTTCCGCTGGAT
TCCCTGCTGATTCTCTCACTGAGGAAGAAATTGACTTCGGCGTAGTTTCCTCTGTTGGTGGC
ATCGAGCAGGTAAATTACATTCTTATTCGAAATCACATTATTGCGAAATGGCGTGAAAATA
TATTCAATTGGGTGACTAAAGAAATGTTTGTTGATTCTATACCACAACATTGTCGTACGCTC
TTAGATTCTGCTTATGATTATTTGGTTACTCATGGATATATAAATTTTGGGGTTGCCCCAGCA
ATCAAGGACAAAATTCCTGTCGGTCTTAGTAAAGGTAATGTGGTTATCATTGGTGCTGGATT
GGCGGGGCTGGCTGCGGCTAGACAGCTAATGAGGTTCGGATTTAAGGTGACGGTTTTGGAA
GGGAGGAAGAGAGCAGGTGGGAGGGTTTATACAAAGAAGATGGAAGGAGGGAATAGGGT
GAGTGCAGCTGCGGATTTAGGTGGGAGTGTATTAACAGGTACATTGGGGAATCCATTAGGG
ATCATGGCAAAACAATTGGGTGCTTCGCTTTTTAAGGTGAGGGATAAGTGTCCACTTTATCG
GATGGATGGGAGTCCGGTGGATCCAGATATGGATATGAAGGTGGAGACGGCTTTTAATCGG
CTTTTGGATAAGGCTAGTAAGCTTAGGCAGTTAATGGGGGAGGTTTCCATGGATGTTTCACT
TGGGGCAGCATTAGAGACGTTTAGACAGGTTTATAGAGATGCAGTAACTGAAGAGGAGAT
TAATCTGTTCAATTGGCATCTTGCAAATTTAGAATATGCAAATGCAGGATTGGTTTCAAAGC
TTTCACTTGCATTTTGGGACCAAGATGATCCATATGACATGGGAGGGGATCATTGTTTCTTG
CCTGGAGGGAATGGAAGGTTGATTCAGGCTCTAGCAGAGAATGTGCCTATTTTATATGAGA
AGACTGTGCATACTATTAGGTATGGAAGTGATGGAGTGCAGGTTACGGCAGGAAATCAGGT
GTTTGAAGGTGATATGGCACTATGTACTGTTCCTCTTGGAGTTTTAAAGAGTGGGTCAATAA
AGTTTGTTCCTGAGTTGCCTCAGAGGAAACTTGATGGGATAAAGAGGTTGGGATTTGGGTTG
TTGAATAAGGTCGCTATGCTTTTCCCTTATGTATTTTGGGGTACAGATCTTGATACCTTTGGG
CATCTTACTGAAGATCCAAGTTGCCGAGGGGAGTTTTTTCTATTTTATAGCTATGCAACAGT
TGCTGGTGGTCCTCTCTTGCTTGCTTTAGTAGCAGGAGAAGCTGCACATAGGTTTGAGACTC
TGCCTCCTACAGATGCAGTAACCCAAGTTCTCCAAATTCTCAAGGGTATATATGAACCGCA
AGGAATCACTGTCCCTGAACCCCTCCAAACCGTCTGTACTAGATGGGGTGGTGATCCCTTTA
GCCTAGGTTCATACTCTAATGTTGCTGTGGGAGCATCTGGAGATGACTATGATATATTAGCA
GAAAGTGTTGGGGATGGAAGACTTTTCTTTGCTGGGGAGGCCACTACACGTCGATACCCTG
CCACCATGCATGGAGCTTTTCTTACTGGGCTCAGGGAAGCTGCAAATATGGCTCAATATGC
CAACGCTCGGACTGCAAAGAAAAAGATAGACAGGAGTCCTTCAAATAATGTTCATTCTTGT
GCTTCCCTCCTTATGGATTTGTTCAGAGAACCTGATTTGGAATTCGGGAACTTTTCTGTTATT
TTTGGTCGAAAGAATGCTGATCCAAAGTCACCAGCAGTTTTGAGGATAACATTCAGTGAGC
CCCGAAAGAAGAATCAGGAAGGTTCAAAGACAGATCAGCAACATTCTAATAAGGTGCTTT
TTCAGCAGCTACAGTCACATTTTAATCAGCAACAACAGCTACATGTTTACACATTGTTATCT
AAGCAACAGGCACTTGAGCTGAGAGAAGTGAGAGGTGGTGATGAGATGAGGTTGAACTAC
CTCTGTGAAAATCTGGGAATTAAGCTGGTGGGACGGAAGGGTTTGGGACCTAATGCTGATT
CTGTCATTGCATCTATAAAAGCACAGAGGGGTGTCCGGAAACCCTCAACAACTCCTGTGGT
TCTAAAATCTGGGGCATCGAAGATGAAACCAGGCACTTTAAAGAAAAAATTCATTAGGAG
GGCTAAAATAGTCCGCAACACTAAAGGGTTGATTCCAGCTCTGGTTCCGAATGCAGCAAAT
GGCAATATGCCAGAGGAAATGAAAGTGATAAAGCTGGCTCCTCCTGACTCCTCTGCTTCGG
GTATGTCTGAAGGCTTCTAG

>GhPAO17
ATGGCTAAGAAGCCGAGAGTTGTGATAATTGGAGCAGGAATGGCTGGTCTTACAGCAGCC
AACAAGCTTTACACTAGTTCAAATGACTTGTTTGAACTATTTGTTGTTGAAGGTGGAACTAG
AATTGGGGGAAGGATCAATACGTCAGAGTTTTATAGTGATAGAGTTGAAATGGGTGCTACT
TGGATTCATGGAATAAAAGGTAGCCCAGTTCATCAAATTGCTCAACAAATCAATGCATTAC

AAGGGTCTGATAAGCCATGGGAGTGTATGGATGGGTTACTTGATGAACCAAAGACCATTGC
TGAAGGTGGGTTCGAGCTAAATGGCTCTATGATTGAACCCATATCGACACTTTTTAAGAACT
TGATGGATTTTGCTCAAGGCAATGAAGCATCCAAATGGTGTTCGATGAGTCGTCGCTTAGGT
AACAAAAGTATTGGTTCTTTTTTAAGAAAAGGCCTTGATGTGTATTGGGATTCTTGTAAAGA
CCATGAAGAGCTTAAAGGGTATGGTAAATGGAGTAGAGAGTTGCTTGAAGAAGCCATTTTT
GCAATGTATGAGAACACACAAAGGACTTATACATCAGCTGGTGATCTTTTCAGTTTAGATT
ATGAAGCAGAAAGTGAGTACCGTATGTTCCCTGGTGAAGAAATCACTATTGGTAACGGATA
TTCCAGTATAATCGAATACCTCGCGTCGGTACTCCCACGGGACGTAATCCAATTAGACCGA
AAAGTCGCTAAAATCGAATGGGATCGTTGTGATTCGAGGCCTGTGAAGATACACTTCTTGG
ATGGATCTTTTGTGTTAGCTGATCATGTTATTGTCACAGTTTCGTTAGGGGTTTTAAAAGCTG
GTATTTGTAATGATCCAGGTTTGTTTAGTCCTCCACTTCCTTCTTTCAAAACGGATGCTATAT
CAAGACTTGGATATGGTGTTGTTAACAAGCTGTTTCTCCGATTAAACGGTAATCGAAAACC
CGAAGAGCTCCCTTCCTTGCAAATGGTGTTTCATCGTTCGGATTCCGAGTTAAGGCATAAAA
AGATCCCATGGTGGATGAGAAGGACAGCTACTTTATCCCCTATTTACAACAATGCAAGTGT
GTTCCTATCTTGGTTTGCAGGGAAAGAAGCTCTTGAACTCGAAAGACTTAGCAACGAAGAG
ATTATAAAGGCGGTAACAACGACAGTTTCGAGTTTATTATCGGAACCCCATAATGAAATCA
TGTCTGATAGCAACTCCAATGGATTTGAAGTGAGCTTTGTTGATGTATTGAAGAGCAAATGG
GGGAGTGATCCATTGTTCTTAGGGTCTTACAGTTATGTTGCTGTTGGATCGTGTGGTGCTGAT
TTTGACACCATGGCTGAACCATTACCTACTGATGTGTATCATCATCATCCACTTCAAATTTTG
TTTGCTGGGGAAGCTACACATAGAACTCACTATTCCACAACTCATGGAGCTTACTTTAGTGG
TATTAGGGAAGCCAATAGGCTTCTTCAACATTATCATTGTGTTGGGGTTTAA

>GhPAO18
ATGGATCTGCTCCGCTTCCAAGGCATTGAGAGGCAGGAGGAGGCAGCAGTTCCCTGTGTCA
TTGTGATAGGAGGTGGTATTTCCGGCCTTGCTGCCGCTCGGACTCTGACTGATGCTTCTTTCA
AGGTAATCCTGTTGGAATCACGAGAAAGACTTGGTGGTCGCATCCATACTGATTTCTCTTTT
GGTTGCCCTGTGGATATGGGAGCTTCATGGAAAGCTTTAGATCTTGATATTTCTCTTACTTTG
CTTCTTTATAAGCTACACGGGGTATGCAATGAGAATCCCTTAGCTCCATTAATATCCTCTCT
GGGCCTTAAATTGTACCGTACTAGTGGTGACAATTCTGTGTTGTATGACCATGATTTGGAAA
GTTATACACTTTTTGATATGGATGGCCGTAAAGTTCCACAAGAGATTGTTGTTGAAGTTGGA
GATGTATTCAAGAGAATACTCAAAGAGACTGAGAAAGTACGGGACGAACACAAGAAGGA
CATGTCAGTCCTTAAAGCAATATCAATTGTGCTAGAAAGGAATCCTGAGTTAAGACAAGAG
GGACTTGCCTATGAAGTGATGCAGTGGTACATATGTAGAATGGAAGCTTGGTTTGCTGCAG
ATACAGATATGATATCCTTGAAATGCTGGGATCAGGCAAATCAACTCCACTTCAACTTTAA
ACTCTACATACATCTTTCACTATTGCAAATCAGAATCTCGAAGCTTTTCAACACTGGTTTGC
ATGAAGTGACGCTGATTAATTTAGAACAAGTCCTTTTGGGTGGTCATGGACTTATGGTGCAG
GGTTATGACCCCATAATAAAAGAACTTGCTAAAGATATTGATGTTCGCTTGAATCATAGTA
GGGTTTCTAAAATATCCAGAGGATGTGATAAGGTGGTGGTCAAAGTTGAGAACGGATTGAG
CTTCATTGCTGATGCTGCTATAGTAACTGTACCCCTCGGGGTTCTTAAAGCCAATTTGATTCA
GTTTGAACCAAAGTTGCCAGAGTGGAAGGTTGCTGCAATTTCAGATATTGGTGTTGGTAAC
GAAAACAAGATTGCCTTACTATTCGACCGAGTCTTTTGGCCAAATGTTGAGCTGTTAGGCAT
TGTTGCACGCACTTCTTATTCTTGTGGTTATTTTCTCAATCTTCACAAGGCAACAGGCCATCC
TATTCTTGTCTATATGGCTGCTGGAAGATTTGCTGACGATCTCGAGAAGTTTTCTGATGAATA
TGCTGTGAAATTTGTGATGTCGCAGTTGAAGAAAATGTTTCCTGATGCAACTGAGCCGGTAC
AATATCTGGTGTCACATTGGGGAACAGATCCAAATTCCCTTGGCTGTTATTCGTATGATCCA
GTCGGGATGGCAGGAGATGTGTATGATAAGCTTAGAGAACCTTTGGATAATCTTTTCTTTGG
AGGGGAAGCAGTTACCGAGGAGCACCAAGGGTCGGTGCACGGAGCTTACTCTTCTGGAGT
CCTGGCTGCCAGAAACTGTGAGAACCATCTCTTAGAGAGATTAGGTGACTTTAGAAAGCTC
CAGCTGATCTCCTTTAGTGGTGATGCATTATTAGAACCCATATTTCCTCTCCAGATATCTAGG
ATGTGA

>GhPAO19
ATGGATTCTTCTTCAAGCTCCGCTGTCATCATCATCGGCGCCGGCATCTCTGGTATATCGGC
GGCGAAGGTTTTGGCTGACAACGGAATTGCGGATTTGCTGATTTTGGAAGCTTCCGGTAGA
ATTGGAGGTAGGATCCTGAAAGAAAGTTTCGGAGGGGTGTCGGTGGAGCTTGGAGCGGGTT
GGATCGCTGGTGTAGGTGGCAAAGCGTCCAATCCCGTTTGGGAGATAGCTTCTAAGTTTGG
CCTCCGAACCTGCTTCTCTGACTACAGTAATGCCCGCTATAACATCTACGATCGGAGTGGG
AAGATCTTTCCGAGTGCAATCGCCGCAGACTCATACAAGAAAGCGGTGGACTCGGCGATA
CAGAAACTAAGGGACCTAGAGTCAAACTCTGTCGAGGATGTCGCCAATGGAGCCGAGTTA
CATTTAACAGCGAAGACACCGATAGAGCTCGCGATTGACTTTATATTACACGATTTCGAGA
TGGCAGGCAAGTTGCACAACTCATTTTTGTTTTCCCCTGTGGAGCCAATATCAACATACGTA
GATTTTGGGGAAAGAGAATTTTTGGTGGCAGATGAAAGGGGTTATGAGTATTTACTGTATA
AAATGGCAGAGGATTTTCTATTGACGTCGGAGGGAAAAATCCTGGATAATCGCCTCAAACT
CAATAAGGTTGTCAGGGAATTACAGCACTCGAGAAACGGCGTCACGGTGAAAACAGAGGA
TGGTTGTGTTTACGAAGCCAACTACGTCATTTTGTCAGCTAGCATCGGTGTTCTCCAAAGCG
ACCTCATTTGCTTCAGGCCGCCCTTGCCCAGGTGGAAAACGGATGCCATAGGGAAATGTGA
TGTGATGGTATATACCAAGATCTTCCTCAAGTTCCCGTATAAGTTTTGGCCTTGTGGGACTG
ACAAAGAGTTCTTCATCTATGCTCACGAGCGGAGAGGCTATTACACGTTTTGGCAGCACAT
GGAAAATGCATACCCTGGTTCGAATATTTTGGTGGTAACATTGACCAATGGTGAATCAAAA
CGTGTTGAAGCTCAATCTGATGAAGAGACGTTAAAGGAAGCAATGGGTGTGCTGAGGGAC
ATGTTTGGGCCCGACATACCGACTGCTACAGATATACTTGTTCCCCGATGGTGGAATAATA
GGTTCCAGCGTGGCAGCTACAGCAATTACCCCATAATCTGTAATAACCAAGTTGTTAATGA
TATTAAGGCCCCAGTTGGACGCATTTTTTTTACTGGTGAACACACAAGTGAAAGATTTAATG
GTTATGTGCATGGTGGATACCTTGCAGGTATTGATACAAGTAAAGCTTTACTGGAAGAAAT
AAGAAAAGACGAAAGAGAAAATGAGAGTAAAAGTTTCTTGCTGGAGCCATTAATAGCATT
GTCAGGGTCATTAACTTTGGCACAGTCGGATGCAGTCTCAGGTCTCCAAAAATGTGAGGTT
CCAACGCAATTATATCTTAGCGGCAAGCTTGGCATTCCAGAAGCAATCTTATGA

>GhPAO20
ATGGAGCCTTCTCCTCCGTCGTCCGTTATCATCATCGGCGCCGGCGTCTCCGGTTTATCGGC
GGCGAAGGTTTTGGCTGAGAATGGAATTGGGGATTTGTTGATCTTAGAAGCGTCTGATAGA
ATTGGCGGTAGGATCCGGAAAGAGAAGTTCGGAGACGTCTCGGTGGAGCTGGGAGCGGGT
TGGATTGCCGGTGTAGGTGGCAAAGAGTCCAACCCGGTTTGGGAGATTGCCGCGAAGCTTG
GCCTCCGAACCTGTTTCTCTGACTACAGCAATGCCCGCTATAACATCTACGATCGGAGCGG
GAAGATATTTCCAAGTGGAATCGCCGCCGACTCGTACAAGAAGGCGGTGGACTCAGCGAT
TCAGAAACTAAAGGGCCTCGAGTCAAACTATGTGGAAGATGCCACCAATAGAACCGACTT
TACTTTAACACCGAAGACACCAATAGAGCTCGCAATTGACTTTATATTACACGATTTTGAG
ATGGCAGAGGTGGAGCCAATATCAACTTACGTAGATTTTGGGGAAAGAGAGTTTTTGGTGG
CAGATGAAAGAGGTTATGAATATTTACTGTATAAAATGGCCGAGGAATTTCTGTTTACCTCG
GAGGGTAAAATCCTGGACAATCGCCTCAAACTGAACAAGGTTGTTAGGGAATTACAGCAC
TCGAGAAACGGCGTCACGGTGAGAACAGAGGATGGTTGCGTTTTCGAAGCCGACTACGTG
ATTTTGTCTGCTAGCATTGGTGTTCTTCAAAGCGACCTCATTTCCTTCAGGCCACCCTTGCCC
AGGTGGAAAACGGAAGCCATAGAGAAATGTGATGTGATGGTGTATACCAAGATCTTCCTC
AAGTTTCCGTATAAGTTCTGGCCCTGTGGGCCTGGAAAAGAGTTCTTTATCTATGCTCACGA
GAGGAGAGGCTATTACACGTTTTGGCAGCACATGGAGAATGCGTACCCTGGTTCGAATATT
TTGGTTGTAACGTTGACGAACGATGAATCGAAGCGTGTGGAATCTCAATCCGATGAAGAGA
CATTGAAGGAAGCTATGGTTGTGCTTAGGGACATGTTCGGGTCTGACATACCCGATGCCAC
TGATATACTTGTTCCCCGCTGGTGGAATAACAGGTTCCAGCGTTGCAGCTACAGCAACTACC
CTATGATATCTAATAACCAAGTCATTAATGATATTAAGGCCCCAGTGGGACGCATTTTCTTT
ACTGGTGAACACACGAGTGAAAGATTTAATGGCTATGTACATGGTGGACACCTTGCAGGCA
TTGACACAAGTAAGGCAGTGCTGGAAGAAATGAGAAAAGATGAAAGACAGAAAGGCAAA
CAGAACCAAAATTTCTTGTTAGAGCCCTTGTTAGCATTGACTCTGACACAGGCGGATGCAG

TCTCAGGTCTCCACAAATGTGATGTTCCTACACAATTGTATCTCAGCGGCAAGCTCGGCATT
CCGGAAGCGATCTTGTGA

>GhPAO21
ATGGAGTCGTCGGACAAGAGTAATCACCAATTGCGTGGAGGTATTTGCTATCCAAATGGGG
CGAGGAGGCCAGTAAGAACACCTTCAGTAATCGTGATTGGGGCTGGAATGGCTGGAATTG
CAGCCGCACGTGCTCTCCATGAAGCCTCATTTCAGGTTACGGTGTTAGAATCCAGGGACAG
AATTGGAGGTCGAGTTCATACTGATTACTCATTTGGTTTTCCTGTTGACCTTGGTGCTTCATG
GTTGCATGGAGTTTCGAAAGAAAATCCCTTGGCACCATTGATCAGTAGACTTGGACTACCA
CTTTATCGGACTAGTGGTGATAACTCTGTGCTGTATGACCATGACTTGGAGAGTTATGCACT
TTTTGATATGGATGGTCATCAAGTTCCACAGGAGTTGGTCACTAAGGTTGGAGAAACATTTG
AGTGCATTTTGGAAGAGGCAATGAGACAAGAGCACAGTGAAGACATGTCCATAAGTAGTG
CTTTCTCAATTGTTTTCGAAAGAAGACCAGAATTAAGGTTGGAAGGGCTTGCACATAAGGT
ACTTCAGTGGTATGTATGCAGAATGGAAGGTTGGTTTGCTTCGGATGCTGATACCATCTCAC
TTAAAAGCTGGGACCAGGCAGAGCTATTACCTGGTGGTCACGGACTCATGGTCAGGGGCTA
TCTTCCTGTCATAAACACTCTGGCCAAAGGTATTGACATCCGCTTGAGCCACAGGGTTACA
AACATAGTGAGGCGTTACACTGGAGTGAAGGTTACTGTGGAAGATGGTACTACATTTGCGG
CGGATGCTGTTATCGTTGCTGTTCCTCTAGGCGTACTAAAAGCCAAGAATATCAAGTTCGAA
CCAAGGCTTCCTGAATGGAAGGAAGCAGCAATTGATGAACTTGGAGTGGGAATTGAGAAT
AAAATTATATTGCACTTTGACAAGGTGTTTTGGCCTAATGTGGAGTTTTTGGGAGTTGTTGCT
GACACATCTTATCATTGCAGCTACTTTCTAAACCTTCATAAGGCGACAGGTCATTCTGTCCT
CGTTTATATGCCTGCTGGGCAGCTGGCCAGAGACATTGAGAAAATGTCTGATGAAGCTGCT
GTGGAGTTTGCTTTTATGCAACTCAAGAAGATCCTTCCAGAGGCATGTGCCCCGATTCAGTA
TCTTGTTTCTCGATGGGGCACAGATGTGAACACACTAGGCTCCTATAGCTATGATGCAGTAG
GCATGTCCCATGATCTGTATGAGAGGCTAAGAGTGCCAGTGGATAACATATTCTTTGCGGG
GGAGGCAACCAGTATGAGCTATCCAGGGTCCATTCATGGTGCATTTTCAACTGGGCAGATG
GCTGCTGAGGACTGTAGGATGCGTGTACTGGAGCGATATGGAGAGTTGAACTTGCTCCAAC
CAGTTATGGGTGAGGAAGCAGGGTTGTGTGTCCCGCTTTTAATAACGCGTTTGTAA

>GhPAO22
ATGGAGACGCCAGTTTCAGAAGGGTTGGTTTCAAAGAGGTCGTTGAGGAAGAAATCCGCA
GTGAAGAATTATGATGAGAATTTAATGGATGAGTTCATAGAGAAGCATATAGGTGGTTCGT
TTAGGAAGATTAGAACAAAGGAGGAGTTGGAGAAAGAGACTGAAACTGAGGCAATGATA
GCATTATCTTTGGGTTTCCCTATTGATGCGCTGATTGAGGATGAAATTAAAGCAGGAGTGGT
AAGAGATATAGGTGGAAAAGAGCAGAATGATTATATTGTTCTTAGGAATCATATCCTTTCT
CGGTGGAGGAGTAATGTACGGATATGGTTATCTAAAGGACATATAAGAGAAACCGTGAGT
AATGAATATGAACATCTGTTGTCTGCTGCTTATGATTTTCTTCTGTATAATGGGTATATTAAT
TTTGGAGTTTCACCATCCTTTTCCTCTTACATCCCAGCGGAGGCAACTGAGGGTTCTGTGAT
AATAGTTGGAGCTGGACTTGCTGGCTTGGCAGCAGCAAGGCAACTTATATCTTTTGGTTTCA
AGGTTGTTGTTATAGAAGGGAGGAATCGACCTGGGGGAAGAGTTTATACTCAACTGATGGG
TAAGAAGGATAAGCGTGGTGCTGTGGATCTTGGTGGTAGTGTAATCACCGGCATCCATGCC
AATCCTCTTGGAGTTCTGGCCCGGCAACTTTCTATTCCACTTCATAAGGTCCGAGATAATTG
CCCTTTATATAAACCTGATGGGGTGCCTGTCAATAAGGTAATCGACTCGAAGACTGAAATG
ATCTTTAACAAGTTGCTTGACAAAGTCAATGAACTGAGAAAAATAATGGGTGGATTTGCTA
ATTATATTTCTCTTGGATCTGTTCTGGAAAAGCTAAGACAGTTGTATGGTGTGGCTAGAAGC
CCAGAGGAGAGACAACTTTTGGAATGGCATCTTGCTAATTTGGAATATGCAAATGCAGGAT
GTCTTTCTGACTTGTCGGCTGCCTACTGGGACCAGGATGATCCTTATGAGATGGGTGGAGAC
CATTGTTTTCTTGCTGGAGGGAACTGGAGATTGATAAAAGCATTATGTGATGGAGTTCCCAT
AATCTATGGGAAAACAGTTGATGCTATTAGATATGGCGTTGAAGGAGTTGAGGTTGTTACC
GGTAAGCAAGCATTCCAAGCAGATATGGTTCTGTGTACTGTGCCTCTTGGAGTCTTGAAGA
GAAGGACCATTAGATTTGAGCCAGAGTTACCTCAAAGAAAGCTAGCTGCAATTGACAGATT

AGGTTTTGGGCTCCTGAATAAAGTTGCCATGATTTTCTCTCATGTTTTTGGGGAGAAGAGTT
GGACACATTTGGATGTCTCAATGATACCAGTGATAACCGTGGGGAGTTCTTTTTATTCTACA
GCTACCACACTGTTTCCGGGGGTCCGGTGTTGATTGCGCTGGTGGCTGGTAAAGCTGCACA
AACATTTGAGCGCACAGATCCTTCACTCTTGCTCCATCGCGTTCTAAGCAAACTTAGAGGTA
TATATGGTCCAAAAGGTGTAGATGTACCTGACCCTATACAGACAATTTGTACAAGATGGGG
AAATGATCCCTTTTCATATGGTTCATACTCTCATGTTAGGGTACAGTCATCCGGCAGGGATT
ATGATATACTTGCAGAAAGTATAGGCAATAGGTTGTTTTTTGCTGGTGAAGCCACAACTCG
GCAATATCCAGCCACCATGCATGGTGCCTATTTGAGTGGGTTAAGGGAAGCTTCGCGTATTC
TCCGTGCCACAAGAGGTCGCCAAAACTACTTTAGGAGGTCTGTGCAGCGGAATGTTGGACC
AAGCAGTGATCAATTGGGTGATCTGTTCAAGATGCCTGATCTAGTATTTGGGAAGTTCTCTT
TTGTGTTCAATCCATTAACAGAGGACCCTAAATCATTGGGGATTTTGAGAATTACTTTTGAT
AATTGCACAGATGATATGAGGAAGGTGCTGGAAAAAAGCTGTGGCCCCCAATCGAATCAA
TCATTGCAGCTGTACGCAGCATTGTCCCGTGAACAGGCACATGAGCTACAGATGGTAACTG
GAGAAGATGAAAGTAAGTTGGTTTATTTGATAAATAATATTGGATTAAAGCTTATGGGAGC
TAATGCTCTTGGAATCACATATAACTCCTTGGTTACTAGCATATCTAGTGCAAGAAAAGGTA
GGAGCAGGTACCGTATATCTGCACCACTGCTAAATACAGTTTAG

>GhPAO23
ATGAACTCACCAAATGAAACCCCTGATCAATTCTCCCAATTCTCCCAATTCCCCCTTCCCCA
TTTCACTCTCACTCCACCTTTACCAAACCCTAACCCTAATTTCCCTCCAACCCCAAACTCAA
CTCCAACACTAACCCCAGTCCTCGATTCTAACACCAATGCTACACCTTCTCTCGATGATCAA
CTTCTACCCTTCCCAGTTCCCAAAAAACGACGACGCGGCAGGCCCCGACGCACTTCCTCAA
CCTCATCGTTTCAACTCCTTAACTTCCCCAACGATTCATTCAACCCCAATGTTCCATACTCTG
ACCCTAACGCTTATTCGATTCCCTCATCAGTAGCGGCGTCGACACAAACTTCACAACCCAA
AATTGCCGACGAGATCATCGTTATCAATAAAGAATCGACGGCTGAGGCTCTCACCGCTCTT
TCCGCTGGATTCCCCGCTGATTCTCTCACTGAGGAAGAAATTGACTTCGGCGTAGTTTCCTC
TGTTGGTGGCATCGAGCAGGTAAATTACATTCTTATTCGAAATCACATTATTGCGAAATGGC
GCGAAAATATATTCAATTGGGTGACTAAAGAAATGTTTGTTGATTCTATACCACAACGTTGT
CGTACGCTCTTAGATTCTGCTTATGATTATTTGGTTACTCATGGATATATAAATTTTGGGGTT
GCCCCAGCAATCAAGGACAAAATTCCTGTCGGTCTTAGTAAAGGTAATGTGGTTATCATTG
GTGCTGGATTGGCGGGGCTGGCTGCGGCTAGACAGCTAATGAGGTTCGGATTTAAGGTGAC
TGTTTTGGAAGGGAGGAAGAGAGCAGGTGGGAGGGTTTATACAAAGAAGATGGAAGGAG
GGAATAGGGTGAGTGCAGCTGCAGATTTAGGCGGGAGTGTATTAACGGGTACATTGGGTAA
TCCATTAGGGATCATGGCAAAACAGTTGGGTGCTTCGCTTTTTAAGGTGAGGGATAAGTGTC
CACTTTATCGGATGGATGGGAGTCCAGTGGATCCAGATATGGATATGAAGGTGGAGACAGC
TTTTAATCGGCTTTTGGATAAGGCTAGTAAGCTTAGGCAGTTAATGGGGGAGGTTTCCATGG
ATGTTTCACTTGGGGCAGCATTAGAGACGTTTAGACAGGTTTATAGAGATGCAGTAACTGA
AGAGGAGATTAATTTGTTCAATTGGCATCTTGCAAATTTAGAATATGCAAATGCAGGATTG
GTTTCAAAGCTTTCACTTGCTTTTTGGGACCAAGATGATCCATATGACATGGGAGGGGATCA
TTGTTTCTTGCCTGGAGGGAATGGAAGGTTGATTCAGGCTCTAGCAGAGAATGTGCCTATTT
TATATGAGAAGACTGTGCATACTATTAGGTATGGAAGTGATGGAGTGCAGGTTACGGCAGG
CAATCAGGTGTTTGAAGGTGATATGGCACTATGTACTGTTCCTCTTGGAGTTTTAAAGAGTG
GGTCAATAAAGTTTGTTCCTGAGTTGCCTCAGAGGAAACTTGATGGGATAAAGAGGTTGGG
ATTTGGGTTGTTGAATAAGGTCGCTATGCTTTTCCCTTATGTATTTTGGGGTACAGATCTTGA
TACCTTTGGGCATCTTACTGAAGATCCAAGTTGCCGAGGGAAGTTTTTTCTATTTTATAGCTA
TGCAACAGTTGCTGGTGGTCCTCTCTTGCTTGCTTTAGTAGCAGGAGAAGCTGCACATAGGT
TTGAGACTCTGCCTCCTACAGATGCAGTAACCCAAGTTCTCCAAATTCTCAAGGGTATATAT
GAACCGCAAGGAATCACTGTCCCTGAACCCCTCCAAACAGTCTGTACTAGATGGGGTGGTG
ATCCCTTTAGCCTAGGTTCGTACTCTAATGTTGCTGTGGGAGCATCTGGAGATGACTATGAT
ATACTAGCAGAAAGTGTGGGGGATGGAAGACTTTTCTTTGCTGGGGAGGCCACTACACGTC
GATACCCTGCCACCATGCATGGAGCTTTTCTTACTGGGCTCAGGGAAGCTGCAAATATGGC

TCAATATGCCAACGCTCGGACTGCAAAGAAAAAGATAGACAGGAGTCCTTCAAATAATGT
TCATTCTTGTGCTTCCCTCCTTATGGATTTGTTCAGAGAACCTGATTTGGAATTCGGGAACTT
TTCTGTTATTTTTGGTCGAAAGAATGCTGATCCAAAGTCACCAGCAGTTTTGAGGATAACAT
TCAGTGAGCCCCGAAAGAAGAATCAGGAAGGTTCAAAGACAGATCAGCAACATTCTAATA
AGGTGCTTTTTCAGCAGCTACAGTCGCATTTTAATCAGCAACAACAGCTACATGTTTACACA
TTGTTGTCTAAGCAACAGGCACTTGAGCTGAGAGAAGTGAGAGGTGGTGATGAGATGAGG
TTGAACTACCTCTGTGAAAATCTGGGAATTAAGCTGGTGGGACGGAAGGGTTTGGGACCTA
ATGCTGATTCTGTCATTGCATCTATAAAAGCACAGAGGGGTGTCCGGAAACCCTCAACAAC
TCCTGTGGTTCTAAAATCTGGGGCATCGAAGATGAAACCAGGCACTTTAAAGCAAAAATTC
ATTAGGAGGGCTAAAATAGTCCGCAACACTAAAGGGTTGATTCCAGCTCTGGTTCCGAATG
CAGCAAATGGCAATATGCCAGAGGAAATGAAAGTGATAAAGCAGGCTCCTCCTGACTCCT
CTGCTTCGGGTATGTCTGAAGGCTTCTAG
